# 2000-year-old pathogen genomes reconstructed from metagenomic analysis of Egyptian mummified individuals

**DOI:** 10.1186/s12915-020-00839-8

**Published:** 2020-08-28

**Authors:** Judith Neukamm, Saskia Pfrengle, Martyna Molak, Alexander Seitz, Michael Francken, Partick Eppenberger, Charlotte Avanzi, Ella Reiter, Christian Urban, Beatrix Welte, Philipp W. Stockhammer, Barbara Teßmann, Alexander Herbig, Katerina Harvati, Kay Nieselt, Johannes Krause, Verena J. Schuenemann

**Affiliations:** 1 Institute of Evolutionary Medicine, University of Zurich, Winterthurerstrasse 190, 8057 Zurich, Switzerland; 2 Institute for Archaeological Sciences, University of Tübingen, Rümelinstrasse 19-23, 72070 Tübingen, Germany; 3 Institute for Bioinformatics and Medical Informatics, University of Tübingen, Sand 14, 72076 Tübingen, Germany; 4 Museum and Institute of Zoology, Polish Academy of Sciences, Wilcza 64, 00-679 Warsaw, Poland; 5 Centre of New Technologies, University of Warsaw, S. Banacha 2c, 02-097 Warsaw, Poland; 6 Senckenberg Centre for Human Evolution and Paleoenvironments, University of Tübingen, Rümelinstrasse 19-23, 72070 Tübingen, Germany; 7 Paleoanthropology, Dept. of Geosciences, University of Tübingen, Rümelinstrasse 19-23, 72070 Tübingen, Germany; 8 Mycobacteria Research Laboratories, Department of Microbiology, Immunology and Pathology, Colorado State University, Fort Collins, USA; 9 Institute of Pre- and Protohistory and Medieval Archaeology, Department of Early Prehistory and Quaternary Ecology, University of Tübingen, Rümelinstrasse 19-23, 72070 Tübingen, Germany; 10 Institute for Pre- and Protohistoric Archaeology and Archaeology of the Roman Provinces, Ludwig Maximilian University Munich, 80799 Munich, Germany; 11 Max Planck Institute for the Science of Human History, Kahlaische Str. 10, 07745 Jena, Germany; 12 Berlin Society of Anthropology, Ethnology and Prehistory, 10117 Berlin, Germany; 13 Museum of Prehistory and Early History, SMPK Berlin, 10117 Berlin, Germany; 14 DFG Centre for Advanced Studies Words, Bones, Genes, Tools: Tracking Linguistic, Cultural and Biological Trajectories of the Human Past, University of Tübingen, Rümelinstrasse 19-23, 72070 Tübingen, Germany

**Keywords:** Ancient DNA, Egyptian mummified individuals, Metagenomics, Leprosy, Hepatitis B virus

## Abstract

**Background:**

Recent advances in sequencing have facilitated large-scale analyses of the metagenomic composition of different samples, including the environmental microbiome of air, water, and soil, as well as the microbiome of living humans and other animals. Analyses of the microbiome of ancient human samples may provide insights into human health and disease, as well as pathogen evolution, but the field is still in its very early stages and considered highly challenging.

**Results:**

The metagenomic and pathogen content of Egyptian mummified individuals from different time periods was investigated via genetic analysis of the microbial composition of various tissues. The analysis of the dental calculus’ microbiome identified Red Complex bacteria, which are correlated with periodontal diseases. From bone and soft tissue, genomes of two ancient pathogens, a 2200-year-old *Mycobacterium leprae* strain and a 2000-year-old human hepatitis B virus, were successfully reconstructed.

**Conclusions:**

The results show the reliability of metagenomic studies on Egyptian mummified individuals and the potential to use them as a source for the extraction of ancient pathogen DNA.

## Background

Over the last decade, advances in next-generation sequencing technologies and the reduction of the associated costs have enabled DNA shotgun sequencing data to be analyzed on a larger scale [1]. These developments fostered the field of metagenomics to provide insights into the composition of the human microbiome and the identification of diseases from which an individual suffers [2, 3]. While most studies focus on the microbiome of the human gut [4], the oral cavity [5], or skin [6] in healthy or diseased modern individuals, the analysis of microbiomes could also provide insights into the health status of individuals in the past. Although the analysis is limited by the preservation of the samples and environmental contamination, e.g., by soil bacteria, studies have confirmed exceptionally well-preserved microbial and human mitochondrial DNA in dental calculus [7–9], which enables the simultaneous investigation of pathogen activity and diet [8, 10]. In addition to dental calculus, metagenomic approaches can be applied to other types of tissues [11–13]. Mummified remains present the opportunity to assess the metagenomic content of various soft tissues, detect specific pathogens within the individual, and perform pathogen genome reconstruction, when combined with genome-wide DNA capture approaches [14]. This also provides the associated insights into the evolution of health, disease, and pathogens. One example is the Tyrolean Iceman, whose colon and stomach were investigated in this manner, allowing for the reconstruction of an ancient *Helicobacter pylori* genome [15]. In addition to natural or spontaneous mummification, there are also anthropogenic mummified individuals that have been deliberately created by the living. Ancient Egyptian mummified individuals, for example, created through artificial mummification processes, have long been considered as potential sources of metagenomic data from the past [16]. However, such remains were burdened by questions regarding their general DNA preservation due to the hot climate, the high humidity level in many tombs, and the chemicals used in mummification techniques, and issues concerning potential contamination of the retrieved DNA [17, 18]. First attempts to perform metagenomic analyses and the identification of potential pathogens led to unreliable results due to methods that were not yet adapted to the analysis of ancient samples, such as the direct PCR [16, 19]. Although recent publications have employed next-generation sequencing in combination with rapidly advancing ancient DNA methodologies to obtain reliable data for human mitochondrial and nuclear genomes [20, 21], a large-scale metagenomic analysis of different tissues from Egyptian mummified individuals has yet to be conducted. The purpose of this study is to investigate the microbial and pathogen content of 133 samples from various tissues to provide insights into the metagenomic composition of the samples and the health status of selected individuals from the Abusir el-Meleq site, located in Middle Egypt. Broadly, our results demonstrate the possibility to get insights into pathogen evolution in unique historical contexts using next-generation metagenomic analysis on Egyptian mummified individuals. Specifically, we were able to retrieve oral microbiome signals from calculus and tooth samples, identify several pathogens in different tissues, and reconstruct the genomes of two of them, namely *Mycobacterium leprae* (*M. leprae*) and hepatitis B virus (HBV).

## Results

### Sample information and dating

In total, 133 samples from 119 mummified ancient Egyptians were included in this study (Additional file 1: Table S1). For sample identifiers, the site, individual ID, and the first letter of the tissue were used to describe each sample (see Additional file 1: Table S2).

All the samples were excavated from Abusir el-Meleq, located in Middle Egypt, by Otto Rubensohn between 1902 and 1905 [22]. The site was occupied from at least 3250 B.C.E. until circa 700 C.E. [23]. The samples were stored in two anthropological collections at the University of Tübingen and Felix von Luschan Skull Collection in Berlin. While some samples were in their original state, others were macerated due to anthropological analysis or conservation problems [24]. Unfortunately, no additional information, such as written documents or the assignment of the mummified individuals to the sarcophagus, could be retrieved for the studied individuals. The human remains have previously been used to study ancient Egyptian embalming resins [25] and ancient human DNA [16, 19, 20], as well as an anthropological examination of the macerated crania [24]. All bone, tooth, and soft tissue samples (Additional file 1: Table S2) were originally sampled by Schuenemann and colleagues [20] and for 90 individuals, the human genetic data has been analyzed [20]. The five dental calculus samples (Additional file 1: Table S2) were newly sampled for this study. Ninety individuals were already radiocarbon dated in previous studies [19, 20], for the remaining 29 individuals, the dates were determined within this study (Additional file 1: Table S2). For thirteen individuals, more than one sample was collected (Additional file 1: Table S2).

The bone, tooth, and soft tissue samples originated from three distinct time periods [20]: 53 from the Pre-Ptolemaic (PPP, 1550–332 B.C.E., including the late New Kingdom, Third Intermediate Period, and Late Period), 47 from the Ptolemaic (PP, 332–30 B.C.E.), and 28 from the Roman Period (RP, 30 B.C.E.–395 C.E.). Newly obtained dental calculus samples date to the First Intermediate (FIP, one sample, dated to 2196–2045 B.C.E.), the Ptolemaic (two samples, 369–40 B.C.E.), and the Roman (two samples, 138–395 C.E.) Period, thus expanding the timeline of the previous study [20].

### General metagenomic assessment

After exclusion of the human genome reads, on average, 24.6% of the reads from the entire data set, corresponding to about 2 million reads per sample on average, could be assigned to the GenBank database [26] with a sequence identity of at least 85%. The unassigned reads likely derived from environmental bacteria that are currently not represented in genome databases. In general, the samples are dominated by bacteria (91.24–99.31% of the assigned reads), followed by viruses (0.65–8.61%) and archaea (0.04–2.57%) (Additional file 1: Table S3). We found a large amount of Clostridia in all samples (Additional file 1: Fig. S1), on average around 70% of the assigned reads. A large amount of Clostridia (15–30%) was also observed in a previous study analyzing the Tyrolean Iceman using the same approach [27]. Damage profiles of the reads mapping to Clostridia varied from 1.8 to 44.5% (Additional file 1: Fig. S2). Members of the class Clostridia are known to be involved in decomposition processes of human remains [28] and were therefore excluded from further analyses.

The main challenge surrounding ancient metagenomic samples is ensuring the authentic microbiome provenience of retrieved microbial DNA. We first compared the bacterial distribution of the samples and the negative controls (Additional file 1: Fig. S3). The negative controls are dominated by Actinobacteria and Proteobacteria and show a higher amount of Bacteroidetes and Negativicutes than the samples. Other phyla, which are present in the samples, are missing in the negative controls. These different profiles support the authenticity of the samples.

**Fig. 1:**
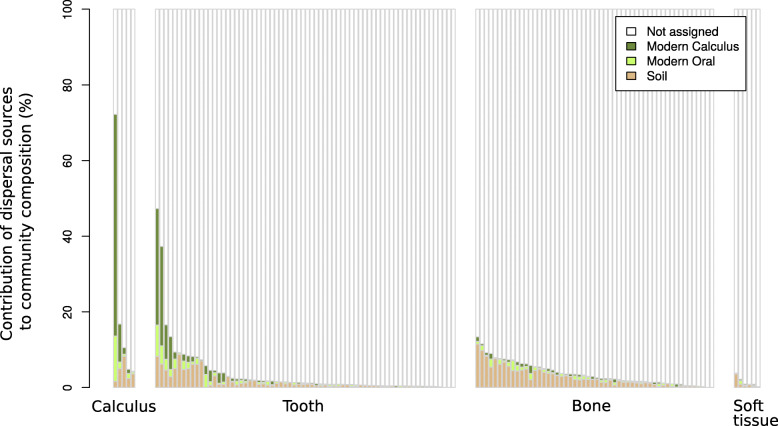
Bacterial community profiles for all bone, soft tissue, and oral samples. SourceTracker2 results, showing the proportion of soil (brown, modern sample taken from a site 250 km northeast of Abusir el-Meleq [68]), modern oral (light green), and modern calculus (dark green) in all oral, bone, and soft tissue samples

However, soon after death, human remains are colonized by microbes responsible for the decay of the body. In addition to microbes living in the soil, these colonizers create a diverse environmental DNA background [10, 29]. We used SourceTracker2 [30] to identify possible sources (soil, modern oral, and modern calculus microbiome) of the bacteria contained in the samples (Fig. 1). In our study, we could not rely on comparative soil samples or metagenomic profiles from different mummified tissues or decaying material except for the oral microbiome [31, 32]. The identification of a tissue-specific microbial signature could not be clearly assessed due to the high amount of Clostridia and the missing authentic soil sample to differentiate between the authentic signal and environmental contamination (Additional file 1: Note 1, Fig. S3) [27, 28, 33, 34]. Therefore, we concentrated on the identification of human pathogens in our assigned metagenomic reads and attempted to authenticate them, using characteristic DNA damage patterns [35] in the bone and soft tissue. Reads mapping to a reference genome without a proper damage profile and with a long fragment length may be originating from recent contamination, bacteria that are a part of the still ongoing decomposition process, or soil bacteria [10]. Lastly, relaxed mapping parameters may also cause misassignments.

We detected authentic ancient DNA signals for five pathogens (Additional file 2: Table S4) in bone samples that were previously shown to contain authentic ancient human DNA assessed by the damage patterns and fragment length [20]. *Proteus mirabilis*, a possible causative agent of symptomatic infections of the urinary tract including cystitis and wound infections [36, 37], was detected in four bone samples (Abusir1608b, Abusir1566b, Abusir1609b, and Abusir1645b). The ancient origin of this pathogen is supported by a short fragment length of 46–53 base pairs (bp) on average (Additional file 2: Table S4) as well as a characteristic ancient DNA damage profile [35] of 10.8 to 16.7% C to T and G to A substitutions at the 5′ and 3′ ends (Additional file 1: Fig. S4A), respectively, matching the profile of the reads mapping to the human mitochondrial genome (Additional file 2: Table S4). Furthermore, *Enterococcus faecalis* and *Enterococcus faecium*, which are known bacterial inhabitants of a healthy intestinal tract, but may also be responsible for causing infections in humans, such as endocarditis and septicemia and urinary tract infections [38]. The detected damage profile shows a base misincorporation frequency of 10.2 to 18.1% at the 5′ and 3′ end, respectively (Additional file 1: Fig. S4B and C, Additional File 2: Table S4) and a short fragment length (42–64 bp on average) (Additional file 2: Table S4), again matching the damage profile of the reads mapping to the human mitochondrial genome (Additional file 2: Table S4).

Furthermore, around 80,000 reads mapped to *M. leprae* in a bone sample of Abusir1630, although the anthropological analysis of this individual did not yield evidence of diagnostic bone deformations to indicate a leprosy infection (Additional file 1: Note 2, Fig. S5) [39–51]. The origin of this pathogen DNA is most likely ancient as a short fragment length (42 bp on average) as well as a damage profile of 11.2% was obtained for all reads mapping to *M. leprae* (Additional file 1: Fig. S4C, Additional File 2: Table S4), which also resembled the damage patterns generated by human mitochondrial DNA for the same individual (52 bp average length and 8.0% DNA damage) (Additional file 2: Table S4).

In addition, 1594 reads mapping to HBV genotype A were identified in the soft tissue and bone sample of Abusir1543 with a damage profile of 9.6% (Additional file 1: Fig. S4E) and a fragment length of 42 bp on average. We also observed a 7.7 times higher number of reads from soft tissue than bone sample (Table 1). The human genomic content of this soft tissue sample (Abusir1543s) was previously analyzed by our group [20] and resulted in a complete mitochondrial human genome derived from a single person with the presence of typical DNA damage patterns (47 bp average length and 8.2 to 9.3%, Additional file 2: Table S4), suggesting an authentic ancient origin.

**Table 1:** Overview of mapping results of samples positive for *M. leprae* and HBV

Sample	# reads prior mapping	Reference genome	# reads mapping	Mean coverage	Genome fraction covered by at least 3 non-duplicate reads	Avg. fragment length
Abusir1630b	70,437,731	*M. leprae*	2,762,380	35.35	97.1	41.8
Abusir1543 (combined)	265,942,056	HBV (AY738142)	1594	20.56	89.3	41.5
Abusir1543 (combined)	265,942,056	Human MT	16,513	46.45	100.0	46.7
Abusir1543b (shallow SG)	3,691,986	HBV (AY738142)	10	0.12	0.0	37.0
Abusir1543s (shallow SG)	2,881,554	HBV (AY738142)	83	0.97	10.4	37.4

For HBV, the mapping results of the shallow shotgun (SG) sequencing of the bone and soft tissue samples are shown as well as the results of the merged libraries, including deeper sequencing of bone and soft tissue samples

### *Mycobacterium leprae* (individual Abusir1630)

**Fig. 2:**
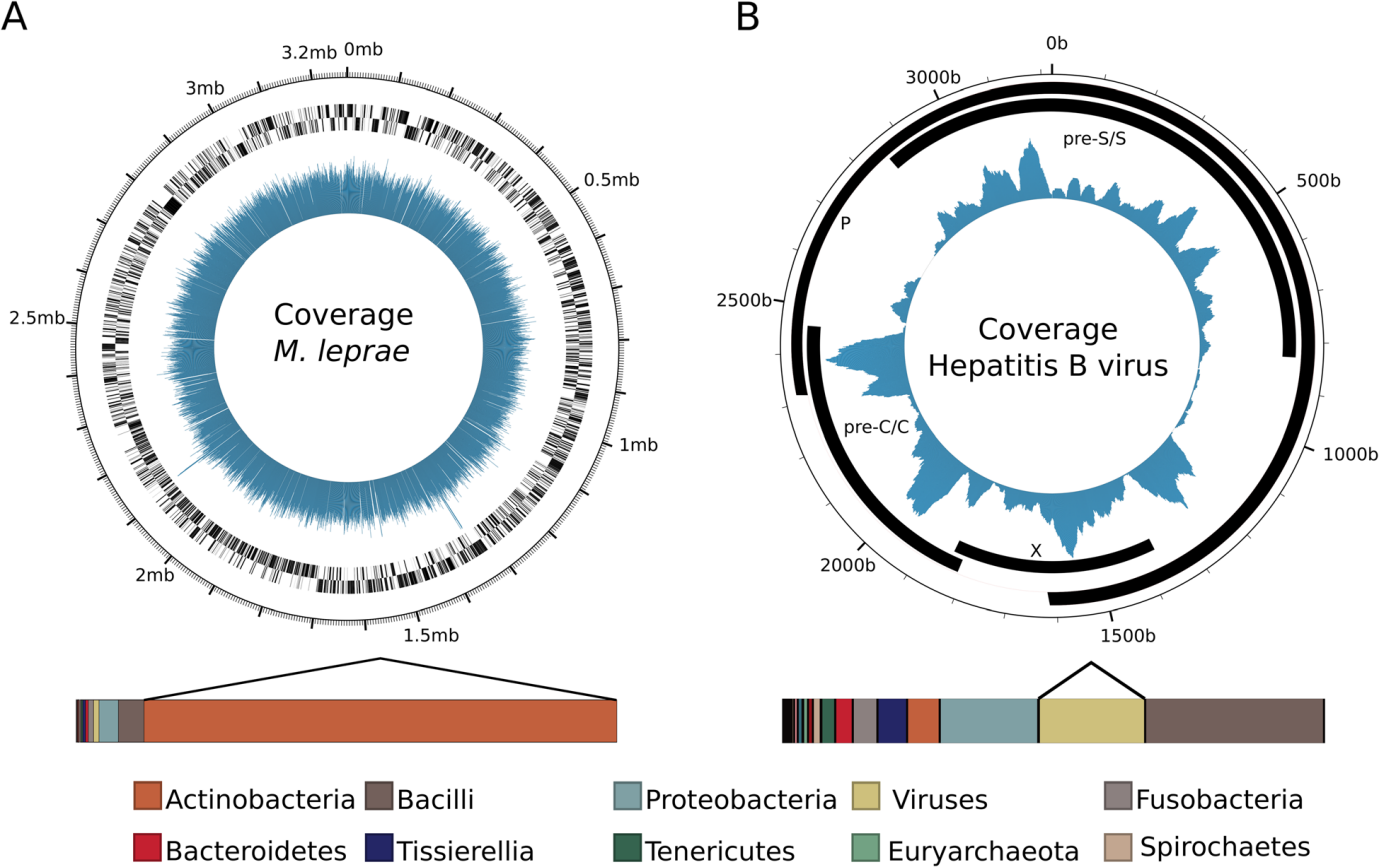
Genome coverage plots. **a** Genome coverage plot of *M. leprae* from the bone sample of individual Abusir1630. The innermost ring represents the coverage per position, the two following rings indicate genes in reverse and forward direction, and the outer ring represents the position in the genome. The stacked bar represents the metagenomic composition of the soft tissue sample Abusir1630b, the lines directing from the stacked bar indicate the phylum to which the pathogen corresponds. **b** Genome coverage plot of hepatitis B virus from individual Abusir1543 based on the combined bone and soft tissue samples. The innermost ring represents the coverage per position, and the following rings indicate the genes in forward direction. As some genes are overlapping, they are distributed over several levels. The outermost ring presents the position in the genome. The stacked bar represents the metagenomic composition of the soft tissue sample Abusir1543s, the lines directing from the stacked bar indicate the phylum to which the pathogen corresponds

Using shotgun sequencing data of individual Abusir1630, 97.1% of the *M. leprae* genome was reconstructed with uniform coverage and a mean coverage of 35.35X (Fig. 2a, Table 1). De novo assembly was not possible due to the very short fragment length (30–73 bp). To the best of our knowledge, Abusir1630 is older than any published *M. leprae* genome to date [52–54], with a radiocarbon age of 342–117 cal B.C.E. [20]. A single nucleotide polymorphism (SNP) analysis of the Abusir1630 *M. leprae* strain was conducted using a total of 3342 informative positions, found in the SNP alignment of all published modern and ancient *M. leprae* genomes [52, 53, 55–61]. This analysis resulted in 47 missense variants, 41 synonymous variants, one stop gain, and one stop loss variant (Additional file 3: Table S5). However, specific phenotypes associated with the SNPs present in Abusir1630 were not identified.

**Fig. 3:**
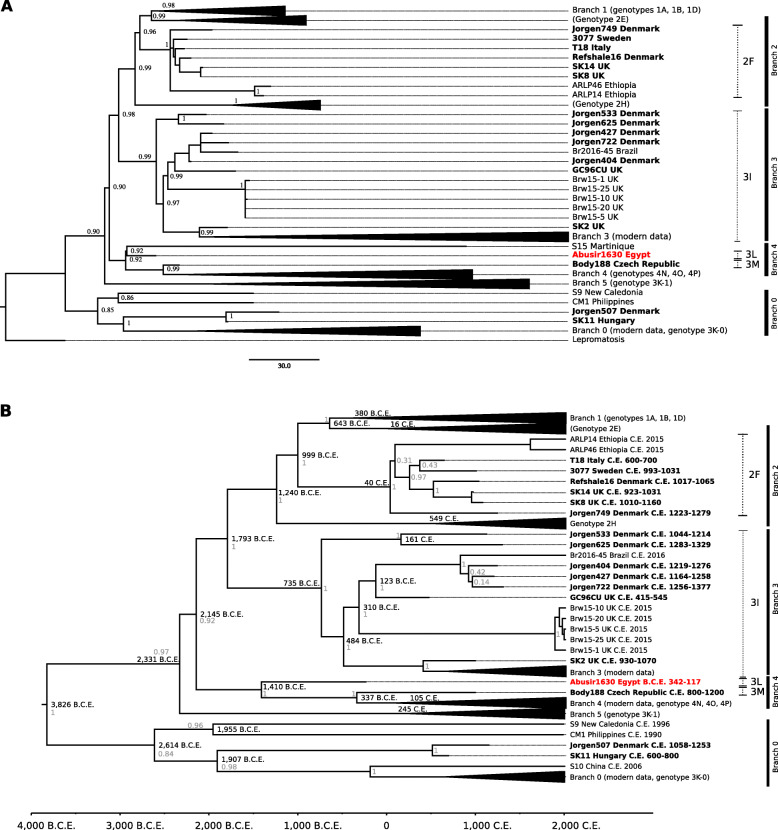
Phylogenetic trees of *M. leprae* genomes. **a** Maximum parsimony tree reconstructed from 3342 informative SNP positions based on 170 *M. leprae* strains [52–61]. The tree is drawn to scale and branch length represents the number of substitutions. *M. lepromatosis* was used as an outgroup. Ancient strains are labeled in bold, and the newly added strain Abusir1630 is labeled in red. Bootstrap values (1000BS) are presented as node labels. Some subtypes are collapsed to simplify the figure. The genotypes are written in brackets or marked with dotted lines. The branches are given on the right side with black bars. **b** Dated Bayesian Maximum Clade Credibility tree reconstructed using 2641 informative SNP positions from 161 *M. leprae* samples [52–61] (without outgroup), strict molecular clock and Bayesian Skyline model. Ancient samples [52–54] are bolded, the newly added genome Abusir1630 in red. The node labels are the median divergence times in years B.C.E. and C.E. The posterior values are given in gray. The genotypes are written in brackets or marked with dotted lines. The branches are given on the right side with black bars

To assess the phylogenetic placement of Abusir1630, maximum likelihood (Additional file 1: Fig. S6A) and maximum parsimony (Fig. 3a, Additional file 1: Fig. S6B) trees were calculated based on all 3342 SNPs. The resulting trees differ slightly; however, the placement of Abusir1630 is consistent. The phylogenetic representation of *M. leprae* is defined by branches (0 to 5) [52, 54] and SNP types, consisting of four SNP types (1–4) and 16 SNP subtypes (A to P) [62]. Although the SNP type nomenclature is constructed using a limited number of SNPs, it is widely used, especially in the field of modern diagnostics [60, 61]. In contrast, the branches are defined based on whole genomes and therefore providing a higher resolution [52]. Both systems are used and popular in the research field of *M. leprae*, which can lead to inconsistencies in some cases. The newly retrieved *M. leprae* genome (Abusir1630) falls in branch 4 together with modern strains from West Africa and Brazil [52, 57, 62] and belongs to the genotype 3L (Additional file 1: Fig. 3a, Table S6), clustering together with S15, which was isolated from a patient from Martinique in 1992 [60]. Besides 3L, branch 4 consists of the SNP types 3M, 4P, 4O, and 4N. Branch 4 contains only one other ancient genome (Body188) from the Czechia (genotype 3M), dated to 800–1200 C.E., which falls together with Abusir1630 and S15. Figure 3a also shows a short branch length of Abusir1630 corresponding to 12 SNPs, indicating that Abusir1630 is genetically very close to the most recent common ancestor (MRCA) of the genotype 3L. Shorter branch lengths are due to a lower number of derived positions, which is often observed in ancient DNA [52]. Additionally, the MRCA of genotype 3L is closely related to the MRCA to the branch 4 (Fig. 3a; with 1 SNP) suggesting that the genotype 3L is basal in the branch 4. As a temporal signal could be detected (methods, Additional file 1: Fig. S7 and S8), the time-aware phylogeny was estimated. The time to the most recent common ancestor (tMRCA) was estimated as 5844 years (y), where years are the years before the most recent sample (4128–8287 y 95% Highest Posterior Density (HPD)) under this model (Fig. 3b, Additional file 1: Fig. S6C), suggesting a divergence time for the leprosy variation that is circa 1300 years older than previously published, however, overlapping with previous assessed confidence intervals [52, 54]. The MRCA of branch 4 was estimated as 3428 y (2633–4494 y 95% HPD) suggesting an introduction of the strain in Egypt between 1410 B.C.E. and 342–117 B.C.E. Since S15 was removed from the divergence time calculation as a hypermutator genotype [60], additional 3L strains are needed to determine the exact chronology of branch 4.

### Hepatitis B virus (individual Abusir1543)

**Fig. 4:**
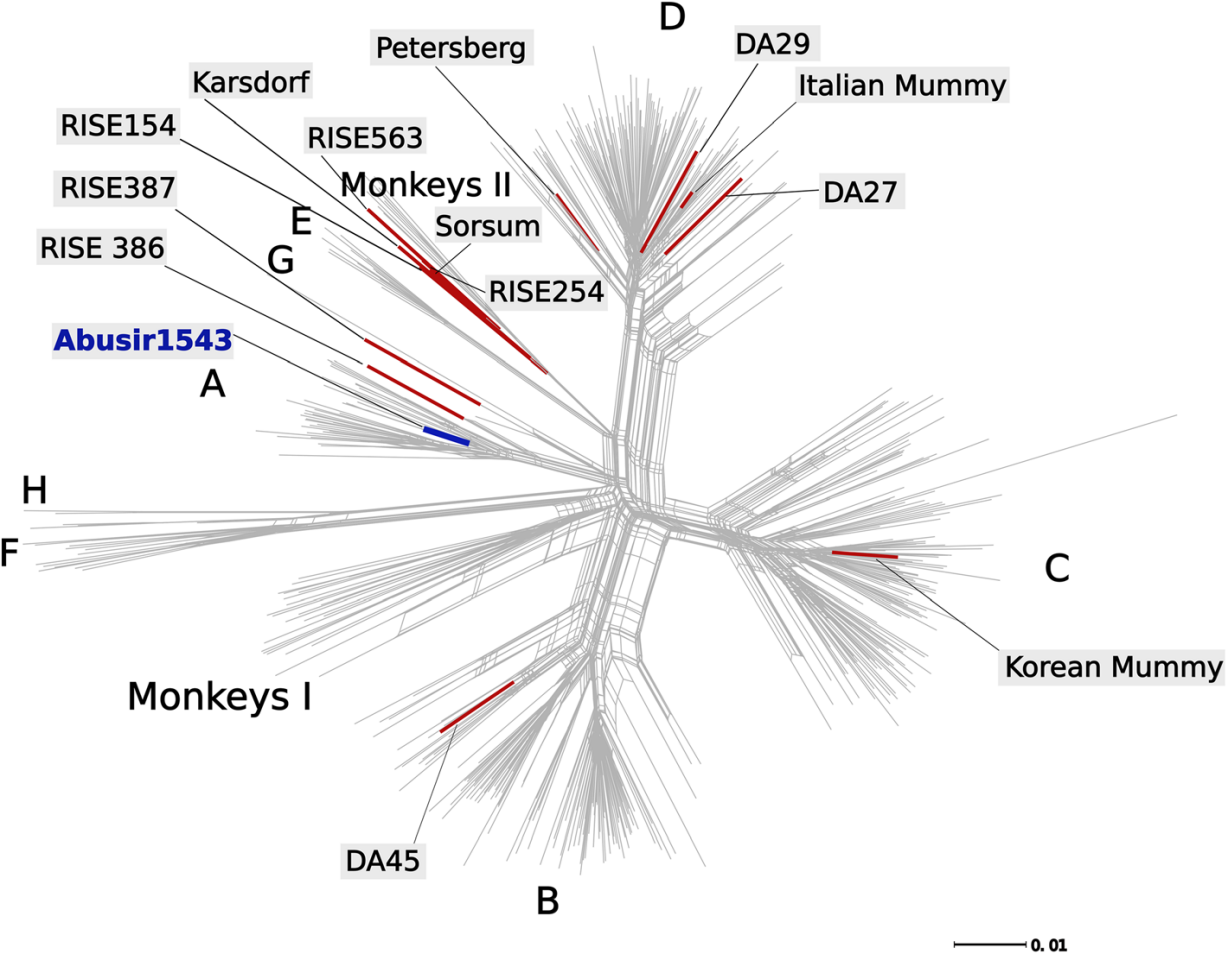
Phylogenetic network for HBV. The network is based on 511 HBV genomes (for references see Additional file 1: Table S7). The published ancient genomes [63–66] are highlighted in red and labeled in black, the newly sequenced genome is highlighted and labeled in blue. The capital letters represent the different clades, the label “Monkeys I” includes all strains from gibbons and orangutans, and “Monkeys II” the strains from gorillas and chimpanzees

Following deeper sequencing of individual Abusir1543 (dated to 54–124 cal C.E. [20]), a total of 1954 reads were found to map to the HBV clone A4 (NCBI accession ID AY738142.1) (genotype A), showing a damage profile of 10.4%. Based on these reads, 96.31% of the genome could be reconstructed with a mean coverage of 20.56X (Fig. 2b, Table 1). To determine the phylogenetic placement of Abusir1543, phylogenetic analyses were carried out based on a data set of 493 modern HBV strains (references see Additional file 1: Table S7), representing the full genetic diversity. Moreover, 18 published ancient strains were added, including Abusir1543 [63–66] (Fig. 4). The phylogenetic network, as well as the maximum likelihood tree (Additional file 1: Fig. S9A), reveals a consistent placement of Abusir1543. It falls within genotype A, which includes modern genomes from Western and Eastern Europe, Western Africa, and Eastern and Southern Asia, as well as ancient genomes from Slovakia, Russia, and Hungary [63, 64]. More precisely, Abusir1543 falls basal to the subclades A1 and A3, representing modern genomes from Asia and Africa, respectively. Moreover, Abusir1543 has a short branch length (Fig. 4), indicating a lower genetic distance to the root, which authenticates an ancient form of this genotype [52].

As recombination is known within the HBV genome [67], we also tested the newly sequenced strain, which resulted in detectable recombination events (Additional file 1: Note 3) [63, 64, 67, 68].

The temporal signal and divergence times were assessed as described in the methods (methods, Additional file 1: Fig. S10 and S11) and the results described in Additional File 1: Note 3, but genetic dating is not expected to yield meaningful results due to recombination and mutation events and may also be influenced by the human-ape barrier crossing [67, 69, 70].

### Oral microbiome assessment

In all tooth and calculus samples, the mitochondrial genomes of the corresponding individuals revealed similar damage profiles, supporting the authenticity of the ancient oral microbiome reads (Additional File 1: Note 4) [71–74]. In contrast to bone and soft tissue samples, the availability of comparative ancient and modern oral microbiomes allowed a more detailed analysis of the oral metagenomic DNA profile recovered from the calculus and teeth.

The assessment of the microbial sources of the five calculus samples shows that the majority of the genetic communities is related to an unknown source (Additional file 1: Fig. S1, Table S8), which is expected for ancient samples [8, 10]. However, we detected a high signal for modern calculus and modern oral communities in two calculus samples (Abusir1519c (70.55%) and Abusir1594c (11.80%), Fig. 1). In general, the calculus samples are dominated by Firmicutes, Actinobacteria, Proteobacteria, Bacteroidetes, Chloroflexi, Fusobacteria, and Spirochetes (Additional file 1: Table S9), which have also been identified as major phyla in ancient calculus in previous studies [8, 75]. Further inspection of these samples showed the presence of typical oral bacteria such as the Red Complex, which consists of *Tannerella forsythia*, *Porphyromonas gingivalis*, and *Treponema denticola* and is related to periodontal diseases [76]. The damage profiles of the reads mapping to the individual bacteria of the Red Complex (13.2–17.6%) are comparable to the profiles of the human mitochondrial DNA from the same individual (10–13%) (Additional file 2: Table S4, Additional File 1: Fig. S12). Two other bacteria (*Filifactor alocis* and *Olsenella uli*) associated with periodontitis and endodontic infections were also identified with damage profiles varying from 16.3 to 28.8% (Additional file 1: Fig. S13A and B, Additional File 2: Table S4) [77, 78].

The analysis of all 68 tooth samples also indicates that the majority of the genetic material contained in the samples could not be assigned to any of the sources (Additional file 1: Fig. S1, Table S8). In four samples (Abusir1580t, Abusir1650t, Abusir1614t, and Abusir1573t), more than 10% of the genetic content could be assigned to oral communities. In the remaining tooth samples, the oral bacterial content varies between 0 and 5.8%. Considering the oral communities of the tooth samples, the dominating phyla are Firmicutes, Actinobacteria, Proteobacteria, Bacteroidetes, Chloroflexi, Fusobacteria, and Spirochetes (Additional file 1: Table S9), as already determined for the calculus samples. Further examination of all tooth samples showed the presence of bacteria corresponding to the Red Complex in 14 samples. The damage profiles of the reads mapping to the individual bacteria of this complex (10.1–21.4%) are comparable to the profiles of the human mitochondrial DNA from the same individual (9.3–13.7%) (Additional file 1: Fig. S12, Additional File 2: Table S4). Moreover, nine tooth samples show the presence of *Filifactor alocis* and *Olsenella uli* with damage profiles varying from 10.5 to 20.2% (Additional file 1: Fig. S13A and B, Additional File 2: Table S4). In comparison to the calculus samples, *Streptococcus mutans*, a significant contributor to tooth decay, was identified in three tooth samples (Abusir1564t, Abusir1655t, and Abusir1627t) (Additional File 2: Table S4) with reliable damage profiles (9.1 to 15.3%) and an average fragment length of 37 to 50 bp (Additional file 1: Fig. S13C, Additional File 2: Table S4).

When comparing the results of the genetic and paleopathological examinations [24], the paleopathological observations were confirmed in 13 of 18 cases (Additional file 2: Table S4). In two samples, oral pathogens could be genetically identified without showing paleopathological evidence. In three samples, oral infections were detected by the paleopathological examination, but no oral pathogen could be genetically identified.

## Discussion

Here, various tissues from mummified individuals spanning 2000 years from the First Intermediate to the Roman Period were analyzed to get insights into the metagenomic composition of the samples, the oral microbial communities, and pathogens associated with ancient Egyptians.

Considering all samples investigated in this study, we observed a high number of DNA reads mapping to Clostridia with varying damage profiles. This can be explained by their role in the decomposition of human biological material [79, 80]. They occur in different stages of the degradation and therefore in different time periods, which leads to varying damage profiles. Moreover, Clostridia also belong to the phylum Firmicutes, which build endospores and can survive in extreme conditions for an extended period of time [81, 82]. Furthermore, it is possible that some of the Clostridia bacteria are still alive or colonized the mummified individuals after they were exhumed 100 years ago [80, 83]. Ongoing studies investigate the necrobiome, describing the community of organisms associated with a decaying corpse [84–86]. It should also be considered that ancient metagenomic samples not only contain the microbes that inhabit humans during life and shortly after death, but also environmental bacteria, which can be investigated using SourceTracker2 [30]. In our case, the analysis was restricted by the lack of soil samples collected from the archeological site associated with the human remains or, more likely, even if collected, no samples were kept because the excavation took place at the beginning of the twentieth century [22]. We therefore used a modern soil sample from a region close to the site, which, however, probably provides very limited insights into the genetic composition of the soil at Abusir el-Meleq. It also has to be considered that the methods and available data hardly can be applied to ancient data to the same extent as to modern data. Reasons are the lack of ancient comparative data, the different sample origin, as well as the lack of extended studies about the necrobiome in mummified individuals. The on average 93.63% of unassigned bacteria (Fig. 1), which is expected for ancient data [10], can therefore be explained by either the absence of a comparative soil sample from this particular archeological site or the presence of bacteria involved in the decomposition of biological material that has yet to be identified in any of the comparative sources. This issue is being investigated in particular for ancient oral samples [8, 87] and paleofeces [88], concluding that the proportion of reads that could not be assigned most likely originates from differences in the studied populations and health status of the individuals [8], as well as bacteria not contained in any of the sources [87].

In addition, we also investigated the different bacterial composition of bone, soft tissue, and oral samples. For bone and soft tissue, the authentication of the tissue-specific bacterial composition was restricted by missing comparative samples and the identification of environmental bacteria, as described above. However, for the oral samples, comparative published calculus and plaque samples were available [31, 32, 87], which allowed us to perform a more detailed analysis of the oral microbiome of the oral samples investigated in this study. Considering only the oral samples, we also detect a high number of bacteria that cannot be assigned to any of the comparative bacterial communities. To a certain extent, this can be explained by the fact that we are comparing ancient samples with modern comparative data sets, which probably does not cover the whole diversity contained in the samples. To address this, more samples are needed to widen our understanding of the evolution of the human oral microbiome in the past. Moreover, as it was demonstrated by previous studies [8, 70], ancient calculus microbiomes are not always directly comparable to modern calculus samples, as the data originate from different populations, different environments, and different health status, which results in different microbial profiles. Apart from the challenges assessing the oral bacterial community, specific oral ancient pathogens were identified in 15 of 68 teeth (22.1%) and two of five calculus samples (40.0%) with authentic damage profiles. The comparison of the genetic identification of the pathogens with the anthropological examination of the samples towards oral infections [24] (Additional file 2: Table S4) shows that the genetic examination confirms the paleopathological results in 13 out of 18 cases. There are also cases, in which a pathogen could be genetically identified, but the individual does not show any visual indication of an infection, and vice versa. This is to be expected, as some disease-associated oral bacteria, such as Red Complex and *S. mutans*, are also present in healthy oral microbiomes [8]. Therefore, the genetic identification of the pathogens does not necessarily mean that the individual had an acute infection, which complicates the paleopathological identification. Our results show that the Red Complex bacteria *T. forsythia*, *P. gingivalis*, and *T. denticola* have long been associated with periodontal disease, despite changes in lifestyle, hygiene, and diet since the First Intermediate Period in Egypt. We also confirm the long-term carriage of opportunistic pathogens in the human oral cavity, including the causative agents of oral diseases.

From bone and soft tissue samples, five pathogens were identified in the studied individuals, and two whole genomes were reconstructed. Regarding the fact that both *P. mirabilis* and Enterococcus species are predominantly present in the urinary tract [38, 89, 90], the detection of these bacteria may be misclassified. However, five of 12 individuals show a clear damage profile, supporting the authenticity of the reads (Additional file 1: Fig. S4). The unclear damage profiles and varying percentages of the damage in the remaining seven samples can be explained by the choice of relaxed mapping parameters, which have been chosen to include DNA modifications that occur over time and are characteristic of ancient DNA [91], or have been caused by environmental contamination.

In contrast to these bacteria, we could firmly reconstruct two full pathogen genomes. The first of them was a *M. leprae* strain dated to 342–117 cal B.C.E. [20]. No physical signs of leprosy infection were identified on the mummified head suggesting an early stage of the infection when characteristic deformations of the facial bones have not yet developed. Little is known about the *M. leprae* genetic diversity in Egypt in ancient and modern times. The only record in Egypt is in an individual dated from the fourth to fifth century [92, 93]. However, because of the poor DNA quality, the exact SNP subtype of this case was not determined and the strain was described as genotype 3K/L/M. The SNP type of the Egyptian genome Abusir1630 is 3L confirming that this SNP type was already present at least 2200 years ago in Egypt. Abusir1630 is located basally with sample S15 from Martinique to the branch 4 and has a short branch length, corresponding to 12 nucleotides, suggesting its basal position within the branch 4 clade. However, S15 is characterized as a hypermutator with a mutation in the base excision repair system (*nth*) [60] and cannot be used as a reference to assess the modern diversity of the genotype 3L. Additional modern and ancient samples from this genotype and specifically from North Africa are needed to investigate the spread and origins of this genotype, as well as the entire branch 4. As of today, Abusir1630 represents the first ancient bacterial genome from Africa and outside Europe as well as the oldest sequenced *M. leprae* genome to the best of our knowledge. Therefore, it could play an important role in better understanding the past diversity of *M. leprae*, as previously published models [54] for the origin and spread of *M. leprae* are only based on ancient data from Europe and modern data [52–54, 56–58]. The models published recently [54] suggest an origin of leprosy in Western Eurasia, from where it has spread, (model1) or *M. leprae* strains were introduced to Europe during and before the Middle Ages from different branches in different regions of the world (model2). Based on the phylogenetic location of Abusir1630, which falls within branch 4 together with modern strains from West Africa and Brazil as well as one ancient strain from the Czech Republic, we conclude that the bacterium likely originated in Eurasia (Branch 0), then proceeded to spread across the world through, for example, trade routes. However, at this time, we cannot favor either one model proposed by Schuenemann and colleagues [54] nor further narrow down the potential region of origin, as the newly added strain Abusir1630 does not fall basal enough to provide more details about the origin of leprosy. Dating analysis indicates a mean tMRCA of all *M. leprae* strains to 3800 B.C.E.; therefore, the mean divergence time is around 1300 years older than previously suggested [54], most likely resulting from the addition of the oldest genome to date, Abusir1630. However, the 95% HPD intervals show an overlap of 1700 years with the previous dating [54]. We also observe a shift of several hundred years back in the estimation of the divergence time in all branch splits, which still overlaps with previous dates [54]. In addition to our molecular dates, there are older osteological cases from India dated to 2000 B.C.E. [94] and 600 B.C.E. [95], or cases from Italy dated to the fourth to third century B.C.E. [96], and Hungary dated to 3780–3650 B.C.E. [97]. However, these cases are based exclusively on osteological analyses without molecular support; therefore, they can only be considered as potential cases and could not be included into our analysis. Nonetheless, these widespread records and in particular the sample from Hungary, which fits our tMRCA, also indicate that the origin of leprosy may date further back in time.

The second pathogen reconstructed genome-wide is an ancient HBV, the causative agent of human hepatitis. The oldest reconstructed genomes are dated to the Neolithic era [63]. In addition to Abusir1543, the virus has already been detected in two other mummified individuals [65, 66] and various skeletal material [63, 64]. In our case, we can report that the preservation of HBV DNA in soft tissue is higher than in bone for the investigated individual. However, the quality of the retrieved sequences does not differ between the two tissues. In previous studies, DNA was successfully extracted from different parts of the body as well [63–66], so we cannot recommend any particular tissue as the best source for investigating the presence of HBV as it strongly depends on the complexity of the library. The investigation of the phylogenetic reconstruction shows that the HBV genome Abusir1543 from Egypt belongs to genotype A clade, clustering together with modern and ancient strains from Western and Eastern Europe, Western Africa, and Eastern and Southern Asia. The two oldest genomes of genotype A were found in Russia, dating to 2264 B.C.E. and 2170 B.C.E., and fall basal in the genotype A clade, suggesting an Asian origin of this clade [64]. Slightly older genomes than Abusir1543 have been discovered in Eastern Europe [64], while Abusir1543 and the modern strains within this genotype are found in Africa. Abusir1543, therefore, is consistent with the hypothesis that the ancestors of genotypes A1 and A3 were carried into Africa subsequently via migration from Eurasia [64], as it represents the so far earliest presence of this genotype in Africa. However, it also does not exclude long-term presence of this genotype in Africa and Eurasia.

Although the phylogenetic trees of HBV have low overall support values, the support values of genotype A are relatively high and the placement of Abusir1543 is consistent. To resolve more details about the past of HBV, more ancient genomes from different regions around the world are necessary as well as more research about the recombination occurring in HBV, which is supported by ongoing discussions concerning if it is reasonable to investigate the divergence time of HBV. On the one hand, several studies show a detectable temporal signal [64, 98], while others strongly advise against dating HBV genomes, as no signal is detected and no meaningful results can be expected due to recombination [63, 66]. Furthermore, the dating is often performed on only one or several selected genotypes or sequences not showing any recombination event [98–100]. As our results show a temporal signal, we performed a divergence time estimation and dated the mean tMRCA of the used HBV data set similar as in a previous study [64] (Additional file 1: Note 3). However, due to the recombination occurring in some strains, including Abusir1543, the results should be considered very carefully. Although there are ongoing discussions on the recombination in HVB, especially with the addition of ancient strains [63, 64], the lack of adapted methods, as existing for other bacteria, e.g., *Treponema pallidum* [68], complicate the analysis and question the results of estimations of divergence times.

## Conclusions

Our analysis shows that Egyptian mummified individuals are a viable source for ancient bacterial and viral DNA. Besides the contamination with environmental bacteria as well as bacteria involved in the process of human decay such as *Clostridia* spp., we demonstrated the successful reconstruction of the microbial composition of various tissues spanning over 1800 years. We could demonstrate the presence of pathogens such as *M. leprae*, HBV, *P. mirabilis*, *E. faecalis*, and *E. faecium* and oral pathogens such as Red Complex bacteria, *F. alocis*, *O. uli*, and *S. mutans* in several samples and reconstruct two highly covered pathogen genomes. With our study, we were therefore able to validate the potential of Egyptian mummified individuals as a source for the recovery of ancient pathogen genomes and, in addition, provide a perspective on pathogen evolution and disease history in very specific, unique historical contexts.

## Methods

### Sample information and radiocarbon dating

In total, 133 samples from 119 individuals were collected from bones, teeth, soft tissues, and dental calculus. The samples were obtained from two anthropological collections at the University of Tübingen and Felix von Luschan Skull Collection in Berlin. The human DNA content of 90 individuals was already analyzed in a previous study [20], while this study investigates the metagenomic and pathogenic content of those and additional samples. The sample identifier contains a short version site name (Abusir) and the individual ID as well as the letter “c” for calculus, “s” for soft tissue, “b” for bone, and “t” for the tooth to distinguish between tissue types. Identifiers containing only 'Abusir' and the individual ID describe the individual. For soft tissue samples, only the most superficial skin covering the spinous process of the exposed vertebra was removed to minimize invasive sampling. Ninety individuals were dated in previous studies [19, 20] (Additional file 1: Table S2). All remaining 29 individuals, for which no C14 dates had been obtained in previous studies, were dated in this study at the Curt-Engelhorn Zentrum Archäometrie GmbH, Mannheim, Germany and the AMS laboratory, ETH Zürich, respectively.

### Sample extraction and library preparation

All bone, tooth, and soft tissue samples were extracted and converted to double-stranded libraries with sample-specific dual barcodes as described in previous studies [20] using well-established protocols [101–103] and were sequenced on Illumina HiSeq 2500 platform at the Institute of Medical Genetics at the University Hospital Tübingen. All pre-amplification steps were carried out in the cleanroom facilities of the Department of Archeo- and Paleogenetics at the Institute of Archaeological Sciences at the University of Tübingen [20], which are specifically dedicated to ancient DNA processing [104].

Due to the presence of *Mycobacterium leprae* DNA in the sample Abusir1630, three additional sequencing libraries of this sample (with 20 μl DNA extract per library) were produced and used for deeper sequencing. For these libraries, a pre-treatment step with uracil-DNA glycosylase (UDG) [105] was added before the final conversion into barcoded sequencing libraries to avoid potential sequencing artifacts caused by deamination of cytosine to uracil due to DNA degradation over time. Negative controls were treated accordingly and carried along in the follow-up experiments. The preparation of the barcoded DNA libraries after UDG treatment was carried out as described above. After the addition of the barcodes, the amplification of the UDG pre-treated and indexed libraries was carried out using 1× Herculase II buffer, 0.4 μM IS5 and 0.4 μM IS6 primer [102], Herculase II Fusion DNA Polymerase (Agilent Technologies), 0.25 mM dNTPs (100 mM; 25 mM each dNTP), and 5 μl indexed library in a total reaction volume of 100 μl with four reactions per indexed library. The thermal profile included an initial denaturation for 2 min at 95 °C and 5–17 cycles of denaturation for 30 s at 95 °C, 30 s annealing at 60 °C and 30 s elongation at 72 °C followed by a final elongation step for 5 min at 72 °C. All splits of one amplified indexed library were pooled, subsequently purified using the Qiagen MinElute PCR purification kit and the DNA was quantified on an Agilent 2100 Bioanalyzer. Based on the high content of endogenous *M. leprae* DNA in this library (4.9%, calculated after initial sequencing of the non-UDG treated library), the amplified UDG libraries were subjected to direct deeper sequencing for genome-wide analysis on an Illumina HiSeq 4000 platform with 2 × 75 + 8 + 8 cycles following the manufacturer’s protocols for multiplex sequencing at the Max Planck Institute for the Science of Human History (MPI-SHH) in Jena.

Dental calculus was retrieved from five different individuals from the Anthropological Collection at the University of Tübingen and these samples were processed in the cleanroom facilities of the Department of Archeo- and Paleogenetics at the Institute of Archeological Sciences at the University of Tübingen as described for the other samples with the following modifications. Before the DNA extraction, the samples were irradiated with UV light for 30 min on each side to remove superficial contamination and then directly ground in the DNA-free tube using a mortar. The subsequent steps follow the above-described protocols except for the amplification and sequencing: the amplification of the indexed libraries was carried out using AccuPrime *Pfx* DNA Polymerase (Invitrogen) in 100 μl reactions. Each indexed library was also divided into 4 reactions with 5 μl template (as described above), 4 units AccuPrime *Pfx* DNA Polymerase, 1× AccuPrime buffer (containing dNTPs), and 0.3 μM IS5 and IS6 primers, and the following thermal profile was used: 2 min initial denaturation at 94 °C, depending on the DNA concentration 4–16 cycles consisting of 30 s denaturation at 94 °C, a 30 s annealing at 60 °C and a 2 min elongation at 68 °C, and a 5 min final elongation at 68 °C. The sequencing was carried out on an Illumina NextSeq500 with 2 × 75 + 8 + 8 cycles using the manufacturer’s protocols for multiplex sequencing at the Institute of Medical Genetics at the University Hospital Tübingen.

As sample Abusir1519 and Abusir1594 showed a positive signal for the oral microbiome, the libraries were sequenced deeper at the Functional Genomic Center in Zurich.

Due to positive signals for the presence of viral DNA of the hepatitis B virus in Abusir1543b, two additional double-stranded sequencing libraries (non-UDG treated) of this sample were produced using 20 μl DNA extract as a template as well as one negative control following the same processing steps in the cleanroom facilities as described above. The indexed libraries and the negative control were amplified according to the same amplification process as described above for the sample Abusir1630 using 11 cycles for the libraries and 16 cycles for the blank. After the amplification, the samples were purified and quantified as described previously and also sequenced on an Illumina HiSeq 4000 platform with 2 × 75 + 8 + 8 cycles using the manufacturer’s protocols for multiplex sequencing at the Functional Genomic Center in Zurich.

### Metagenomic screening

Metagenomic screening of the 133 samples was carried out with MALT [106] using all complete bacterial, viral, and archaeal genomes in GenBank [26] as a reference (version May 2018). MALT was executed with the following mapping parameters: Only reads with a minimum 85% identity (−−minPercentIdentity) were considered as a possible match to the reference. Moreover, the minimum support parameter (−−minSupport) was set to 5, i.e., only nodes with minimum support of five reads are kept. BlastN mode and SemiGlobal alignment were applied and a top percent value (−−topPercent) of 1 was set. All other parameters were set to default. MALT results were analyzed and visualized using MEGAN6 [34]. The ancient origin of the read mapping to prevalent genomes was assessed by calculating damage profiles consisting of base misincorporations at the 5′ and 3′ end of the fragments typical for ancient DNA [35] using DamageProfiler [107].

In particular, we compared and analyzed all calculus and tooth samples in order to assess the bacterial composition of the oral flora in different time periods. To control for variation, the counts were normalized by multiplying the ratio |C|/|S| with a minimum size of any of the samples, where |C| is the number of reads assigned to a specific node, and |S| the total number of reads assigned for sample S [33].

### Authentication of ancient DNA

The authenticity of the samples has been assessed by applying a number of methods and criteria. Ancient DNA is characterized by a short fragment length (about 40–60 bp) and increasing base substitution of cytosine by thymine towards the end of the fragment [35]. DamageProfiler [107] has been used to evaluate fragment lengths and nucleotide misincorporation patterns of the described samples, all of which showed levels that are characteristic for ancient DNA.

### Content of endogenous DNA (SourceTracker2)

To assess the content of endogenous DNA in the sample, we used SourceTracker2 [30]. A subset of supra- and subgingival plaque samples from the human microbiome project [31, 32], an Egyptian soil sample [108], and modern calculus samples [87] were used as a source. As this project lacks soil samples from the site, a previously published soil sample was used [108]. This sample was taken from a site 250 km northeast of Abusir el-Meleq from a desert uninfluenced by humans to study the microbial composition. The accession numbers of all samples used for SourceTracker2 analysis are given in Additional file 1: Table S8.

### Data processing of sample Abusir1630b (*M. leprae*)

#### Read processing, mapping, and variant calling

Sample Abusir1630 was further processed using EAGER version 1.92.55 [109]. To summarize, the sequencing quality was inspected with FastQC version 0.11.5 [110], and the reads were adapter trimmed with AdapterRemoval ver. 2.2.1a [111] and subsequently aligned to the *Mycobacterium leprae* TN chromosome (NC_002677.1) using CircularMapper version 1.0 [109] with a minimum quality score of 20 and a maximum edit distance of *n* = 0.2. Duplicates were removed with DeDup version 0.12.1 [109], and DamageProfiler v0.3.12 [107] was used to investigate the damage patterns. The Genome Analysis Toolkit (GATK) version 3.8.0 [112, 113] was used to generate a mapping assembly and SNP calling. The reference base was called if the position was covered by read at least five times and the quality score was at least 30. The base was called as a SNP if the quality score was at least 30 and 90% of the mapped reads contained this variant.

SNP alignment of all modern and ancient *M. leprae* genomes was analyzed with the genomic variant annotations and functional effect prediction toolbox SnpEff [114].

#### SNP typing

The genotyping of the strain was performed using an established method [62]. Briefly, there are 84 informative markers (78 SNPs and six InDels in homopolymeric tracts) used for the classification in 16 SNP subtypes of *M. leprae* [62]: 1A-D, 2E-H, 3I-M, and 4 N-P. For a more straightforward application, the SNP types (SNP type 1–4) and the SNP subtypes (A-N) could be determined using a combination of three and 16 loci, respectively. These loci are described by [56]. The *M. leprae* genome was newly enumerated between the publication of Monot and colleagues in 2009 and Truman and colleagues in 2011 [56, 62]. Therefore, there is a small difference between the SNP positions in the two papers (Additional file 1: Table S6). We used the reference NC002677.1 with the old numeration. Based on both publications, Abusir1630 is SNP subtype 3L. The SNP positions were also checked manually using the Integrative Genomics Viewer [115].

#### Anthropological analysis

For the osteological examination, standard osteological procedures and recording forms [40] were used for the detailed description and study of preservation, age at death, sex, stature, anatomical variants, and pathologies. The age at death determinations were based on methods evaluating dental maturation [42] and the fusion of cranial sutures [43, 44]. We determined sex through an evaluation of sexually dimorphic skeletal traits of the skull. According to standard recommendations [40, 41, 44, 45], we assessed the expression of following cranial characteristics, provided that skeletal preservation was sufficient: the glabella, superciliary arch, zygomatic and mastoid processes, occipital protuberance, nuchal plane, mentum, and gonion.

In an explorative manner, skeletal and dental pathologies were identified and recorded, and skeletal remains examined for bone remodeling, signs of inflammation, trauma, lytic defects, other pathological abnormalities, as well as degenerative joint diseases (DJD) [46, 47].

For further radiological evaluation of the skeletal and cartilaginous structures of the midface, a clinical high-resolution spiral CT scan was performed on a dedicated breast CT scanner (“nu:view”, AB-CT—Advanced Breast-CT GmbH, Erlangen, Germany; field of measurement: Ø 200 mm × 160 mm, tube voltage: 60 kV, tube current: 125 mA, isotropic voxel size: 0.15 mm) at the University Hospital of Zürich.

#### Phylogeny

The phylogenetic tree was created including 16 published ancient [52–54] and 154 modern [52, 55–61] *M. leprae* genomes including the newly sequenced genome of sample Abusir1630. We excluded strain SK27 [53] as this strain does not fulfill our quality criteria of at least 5X coverage of at least 80% of the reference genome. All genomes were equally processed as sample Abusir1630 (described above). A SNP was used when it was called in at least one sample. The SNP alignment was built with MUSIAL [116] and a maximum parsimony tree was calculated with MEGAX [117] using partial deletion on a site (cutoff of 80%) and 1000 bootstraps. In addition, a maximum likelihood tree was generated using PhyML version 3.1 [118] with 100 bootstraps and optimizing tree topology, branch length, and rate parameters.

#### Beast analysis

We used the Bayesian framework BEAST v2.5.0 [119] to estimate divergence times and substitution rates. In total, 161 ancient and modern strains were used. Strains with unusually long branch lengths, but having approximately the same number of InDels, and deleterious mutations in the endonuclease III gene *nth* [60], are called hypermutated strains. These strains (85054, Amami, S15, Br14–3, Br2016–15, Zensho-4, Zensho-5, and Zensho-9) were excluded from the analysis, as described by Benjak and colleagues [60]. Moreover, all positions were excluded that occur in known repeat regions and rRNA and the positions covered by the negative control sample SK12 [52]. The Bayesian substitution model testing (bModelTest, resulting with model 123421 [120]), a relaxed clock, and a Bayesian Skyline model for variable population size were used for the analysis, as it was supported best by our data. The tip dates for the ancient samples were selected as the mean value from the dating intervals. For modern samples, the isolation dates were used. The Markov Chain Monte Carlo was run with 100,000,000 iterations rejecting the first 10,000,000 as burn-in. The stability of the results was assessed using Tracer version 1.7.1 [121]. All trees were visualized with FigTree version 1.4.4 (http://tree.bio.ed.ac.uk/software/figtree/).

#### Temporal signal

We performed a date randomization test in order to test the temporal signal in the data set and validate the reliability of the Bayesian dated phylogenetic inference [122] for the *M. leprae* data set. We reran the BEAST analysis 10 times using tip calibration dates randomly reassigned among the samples and otherwise the same settings as the original BEAST analysis (described in the previous paragraph). The test indicates sufficient temporal signal in the data set for molecular clock calibration, as the rate estimate for the randomized calibration runs is not overlapping with the estimate for the original data (Additional file 1: Fig. S7), hence supporting our BEAST analysis results. Moreover, a BEAST analysis excluding Abusir1630 indicates that this sample is not a major driver of the molecular rate estimate (Additional file 1: Fig. S7). In addition, the temporal signal was investigated using TempEst [123] resulting in *R*^2^ = 0.39 and a correlation coefficient of 0.62 (Additional file 1: Fig. S8). Excluding strain Abusir1630 from both analyses does not change the results.

### Data processing individual Abusir1543 (hepatitis B virus)

#### Read processing, mapping, and variant calling

The paired-end sequencing reads were analyzed with EAGER version 1.92.55 [109]. As paired-end sequencing was performed, overlapping reads were merged, and the adapters were removed with AdapterRemoval version 2.2.1a [111]. All merged and non-merged reads were first mapped against a combined reference consisting of 16 HBV genomes covering all genotypes, including human and monkey strains (AY738142, AB073846.1, M12906, M32138, AB032431, AB036910, AB064310, AY090454, AB032433, AF222323, AJ131567, AY330911.1, AJ131571.1, U46935.1, FM209516.1, AF193863.1), with BWA aln version 0.7.17 [124] and mapping quality 0 to allow multiple mappings. Afterward, the reads were mapped again against the closest reference (AY738142, Genotype A) using CircularMapper version 1.0 [109] to take the circular structure of the HBV genome into account. The consensus sequence was generated using GATK UnifiedGenotyper version 3.8–0 [125] and VCF2Genome version 0.91 [109]. The reference base was called if the position was covered at least three times and the quality score 30 at a minimum. The base was called a SNP if the quality score was at least 30 and 90% of the mapped reads contained this variant.

#### Phylogeny

For the phylogenetic analysis of HBV, different approaches were applied: a network structure using SplitTree version 4.15 [126], a maximum likelihood tree, and a maximum parsimony tree. The tree-like network is based on an alignment of 511 modern and ancient HBV genomes, as already used and established in a previous study [63], using Parameters NeighborNet [127] with uncorrected P distances. The maximum likelihood and maximum parsimony tree are based on a selected subset of the alignment described above, containing all ancient and 111 modern human and nonhuman primate HBV genomes [63–66], as already used in a previous study [64], and including our new genome Abusir1543. The sequences were aligned with MAFFT version 7.407 [128] using the linsi algorithm. Based on this alignment, a maximum likelihood, as well as a maximum parsimony tree, was calculated using PhyML version 3.1 [118] and 100 bootstraps and MegaX [117] using all sites and 100 bootstraps, respectively.

#### Recombination analysis

The recombination analysis was performed based on an alignment of 52 representative sequences (Additional file 1: Table S7), consisting of one representative of each subgenotype, 17 ancient [63–66], and the newly sequenced strain. We used the methods RDP [129], GENECONV [130], Chimaera [131], MaxChi [132], BootScan (secondary scan) [133], SiScan (secondary scan) [134], and 3Seq [135] within the RDP4 software [136]. We used a window size to 100 nucleotides and the parameter circular genome without reference.

#### Beast analysis

We used the Bayesian framework BEAST version 2.5.1 [119] to estimate divergence times and substitution rates using all 129 genomes (Additional file 1: Table S7). The Bayesian substitution model testing (bModelTest, resulting with model 123456 [120]), an uncorrelated log-normal relaxed clock, and Bayesian skyline tree prior for variable population size were used for the analysis, as they were supported best by our data. The tip dates for the ancient samples were selected as the mean value from the dating intervals. For modern samples, the isolation dates were set. The Markov Chain Monte Carlo was run with 100,000,000 iterations rejecting the first 30,000,000 as burn-in.

#### Temporal signal

We performed a date randomization test in order to test the temporal signal in the data set and validate the reliability of the Bayesian dated phylogenetic inference [122] for the HBV data set. We reran the BEAST analysis 10 times using tip calibration dates randomly reassigned among the samples and otherwise the same settings as the original BEAST analysis (described in the previous paragraph). The test indicates sufficient temporal signal in the data set for molecular clock calibration, as the rate estimate for the randomized calibration runs is not overlapping with the estimate for the original data (Additional file 1: Fig. S10). Moreover, a BEAST analysis excluding Abusir1543 indicates that this sample is not a major driver of the molecular rate estimate (Additional file 1: Fig. S10). In addition, the temporal signal was investigated using TempEst [123] resulting in *R*^2^ = 0.31 and a correlation coefficient of 0.55 (Additional file 1: Fig. S11A). An equivalent TempEst analysis excluding strain Abusir1543 does not change the results of the temporal signal (*R*^2^ = 0.30 and a correlation coefficient of 0.55 (Additional file 1: Fig. S11B).

## Supplementary information

**Additional file 1: Fig. S1.** Number of reads mapping to Firmicutes. **Fig. S2.** Combined damage profiles of Clostridia. **Fig. S3.** Metagenomic composition of all samples. **Fig. S4. **Combined damage profiles of pathogens identified in bone samples. **Fig. S5.** Anthropological analysis of individual Abusir1630. **Fig. S6.** Phylogenetic trees of *M. leprae* genomes. **Fig. S7.** Date Randomization test for the *M. leprae* data set. **Fig. S8.** TempEst analysis for the *M. leprae* dataset. **Fig. S9.** Phylogenetic trees of Hepatitis B virus genomes. **Fig. S10.** Date Randomization test for the HBV data set. **Fig. S11.** TempEst analysis for the HBV dataset. **Fig. S12.** Combined damage profiles of Red Complex bacteria. **Fig.** **S13.** Combined damage profiles of oral pathogens. **Table S1.** Overview of the number of samples and sampled tissue. **Table S2.** Sample overview. **Table S3.** Microbial composition of the samples. **Table S6.** Approach to determine the genotype of Abusir1630. **Table S7.** HBV strains used for analysis. **Table S8.** Accession numbers of source samples used for SourceTracker2 analysis. **Table S9.** Bacterial composition on Phylum level. **Table S10.** Mapping result of Abusir1519c to the human mitochondrial genome.

**Additional file 2: Table S4.** Mapping results of the metagenomic analysis.

**Additional file 3 Table S5.** Effects of SNPs on the individual *M. leprae* genomes.

## Data Availability

The sequencing data for all 119 individuals are deposited in the European Nucleotide Archive (http://www.ebi.ac.uk/ena) with the study ID PRJEB33848 [137].

## Abbreviations

*M. leprae*: *Mycobacterium leprae*
HBV: Hepatitis B virus
PPP: Pre-Ptolemaic period
PP: Ptolemaic period
RP: Roman period
FIP: First intermediate period
bp: Base pairs
SNP: Single nucleotide polymorphism
MRCA: Most recent common ancestor
tMRCA: Time to the most recent common ancestor
y: Years
HPD: Highest Posterior Density
