# Supplementary material for: 2000-year-old pathogen genomes reconstructed from metagenomic analysis of Egyptian mummified individuals
Source: BMC Biol. 2020 Aug 28;18:108. doi: 10.1186/s12915-020-00839-8 (PMC7456089; doi:10.1186/s12915-020-00839-8)
Supplement: Supplementary file 1 — Additional file 1: Fig. S1. Number of reads mapping to Firmicutes. Fig. S2. Combined damage profiles of Clostridia. Fig. S3. Metagenomic composition of all samples. Fig. S4. Combined damage profiles of pathogens identified in bone samples. Fig. S5. Anthropological analysis of individual Abusir1630. Fig. S6. Phylogenetic trees of M. leprae genomes. Fig. S7. Date Randomization test for the M. leprae data set. Fig. S8. TempEst analysis for the M. leprae dataset. Fig. S9. Phylogenetic trees of Hepatitis B virus genomes. Fig. S10. Date Randomization test for the HBV data set. Fig. S11. TempEst analysis for the HBV dataset. Fig. S12. Combined damage profiles of Red Complex bacteria. Fig. S13. Combined damage profiles of oral pathogens. Table S1. Overview of the number of samples and sampled tissue. Table S2. Sample overview. Table S3. Microbial composition of the samples. Table S6. Approach to determine the genotype of Abusir1630. Table S7. HBV strains used for analysis. Table S8. Accession numbers of source samples used for SourceTracker2 analysis. Table S9. Bacterial composition on Phylum level. Table S10. Mapping result of Abusir1519c to the human mitochondrial genome. [file 12915_2020_839_MOESM1_ESM.pdf]

Additional File 1

2,000-year-old pathogen genomes reconstructed from metagenomic analysis of Egyptian mummified individuals

Judith Neukamm (a,b,c), Saskia Pfrengle (a,b), Martyna Molak (d,e), Alexander Seitz (c), Michael Francken (f,g), Partick Eppenberger (a), Charlotte Avanzi (h), Ella Reiter (b), Christian Urban (a), Beatrix Welte (i), Philipp W. Stockhammer (j,k), Barbara Teßmann (l,m), Alexander Herbig (k), Katerina Harvati (f,g,n), Kay Nieselt (c), Johannes Krause (k,b,f,*), and Verena J. Schuenemann (a,b,f,*)

a Institute of Evolutionary Medicine, University of Zurich, Winterthurerstrasse 190, 8057 Zurich, Switzerland.

b Institute for Archaeological Sciences, University of Tübingen, Rümelinstrasse 19-23, 72070 Tübingen, Germany.

c Institute for Bioinformatics and Medical Informatics, University of Tübingen, Sand 14, 72076 Tübingen, Germany.

d Museum and Institute of Zoology, Polish Academy of Sciences, Wilcza 64, 00-679 Warsaw, Poland.

e Centre of New Technologies, University of Warsaw, S. Banacha 2c, 02-097 Warsaw, Poland.

f Senckenberg Centre for Human Evolution and Paleoenvironments, University of Tübingen, Rümelinstrasse 19-23, 72070 Tübingen, Germany.

g Paleoanthropology, Dept. of Geosciences, University of Tübingen, Rümelinstrasse 19-23, 72070 Tübingen, Germany.

h Mycobacteria Research Laboratories, Department of Microbiology, Immunology and Pathology, Colorado State University, USA.

i Institute of Pre- and Protohistory and Medieval Archaeology, Department of Early Prehistory and Quaternary Ecology, University of Tübingen, Rümelinstrasse 19-23, 72070 Tübingen, Germany.

j Institute for Pre- and Protohistoric Archaeology and Archaeology of the Roman Provinces, Ludwig Maximilian University Munich, 80799 Munich, Germany.

k Max Planck Institute for the Science of Human History, Kahlaische Str. 10, 07745 Jena, Germany.

l Berlin Society of Anthropology, Ethnology and Prehistory, 10117 Berlin, Germany.

m Museum of Prehistory and Early History, SMPK Berlin, 10117 Berlin, Germany.

n DFG Centre for Advanced Studies 'Words, Bones, Genes, Tools: Tracking Linguistic, Cultural and Biological Trajectories of the Human Past', University of Tübingen, Rümelinstrasse 19-23, 72070 Tübingen, Germany.

*Corresponding authors. E-Mail: Johannes Krause (krause@shh.mpg.de), Verena J. Schuenemann (verena.schuenemann@iem.uzh.ch)

Note 1: General metagenomic assessment

Judith Neukamm

The first bacterial screening showed a high amount of Clostridia in all samples (Figure S1), on average around 70% for each tissue and time period. Similar amounts were also observed in a previous study [27]. Members of the phylum Clostridia are known to be involved in the decomposition of human remains, especially in wounds [28]. As these bacteria are very dominant and bias the composition, all bacteria from the phylum Clostridia were excluded from further analysis. The general amount of genomic microbial content contained in the samples varies from less than 17,089 (1.55% DNA) to 1,843,564 reads (40.86% DNA). The reads are assigned to bacteria (91.24%-99.31%), viruses (0.65%-8.61%), and Archaea (0.04%-2.57%) (Table S3). To address the bias of different sample sizes, the read counts were normalized, meaning the number of reads of each sample is normalized by dividing the number of assigned reads by the current sample size, and then multiplied by the smallest sample size [30] using MEGAN6 [31]. The samples were then grouped by tissue type within the same time periods and the general metagenomic composition between and within all time periods covered by the samples was analyzed. The results of the comparisons are described in the following paragraphs.

Screening of bone samples. In all time periods, the composition of the bone samples is dominated by Firmicutes, followed by Actinobacteria and Proteobacteria (Figure S3A). The proportion of Firmicutes stays consistent (on average: PPP: 58.44%, PP: 48.69%, and RP: 58.66%), while the amount of Actinobacteria (on average: PPP: 17.38%, PP: 27.24%, RP:

27.86%) increases to 27.24% in the PP and RP; the proportion of Proteobacteria is consistent in the PPP (18.18%) and PP (19.19%) but decreases in the RP (9.96%).

Tissue comparison within individuals. The comparison of various tissues from a single individual shows a different distribution of phyla within the individuals and tissues (Figure S3B). However, some similarities can be observed. In all samples, the main phyla detected are Firmicutes, Actinobacteria, and Proteobacteria. On average, the bone samples show a higher percentage of Firmicutes (46.20%) than the soft tissue (41.19%) and tooth (12.80%) samples. With the exception of Abusir1858, all bone samples have a higher percentage of Proteobacteria compared to the soft tissue sample from the same individual. The comparison of the samples (soft tissue, bone, and tooth) taken from individual Abusir1564 shows a percentage of 18.95% to 42.02% of Firmicutes. The soft tissue has a low percentage of Actinobacteria (20.55%) but a high percentage of Proteobacteria (42.02%), whereas the bone sample shows a relatively equal distribution (Actinobacteria: 31.31%, Proteobacteria: 32.30%) of those phyla. However, the data from the tooth sample demonstrate the opposite distribution of the soft tissue sample with a low percentage of Proteobacteria (10.08%) and a high percentage of Actinobacteria (69.19%).

Oral samples. Overall, the microbial composition does not change in the PPP and PP oral samples, the percentage of Firmicutes increases from the FIP to the RP (FIP: 11.81%, PPP: 22.88%, PP: 38.50%, RP, 50.66%) (Figure S3C and D, Table S9). Moreover, ancient samples show a smaller amount of Bacteroidetes compared to modern samples (ancient: 2.13% on average and modern: 19.05%), but both showed the most dominant phyla being Firmicutes, Actinobacteria, Proteobacteria, Bacteroidetes, and Fusobacteria (Table S9).

Note 2: Anthropological analysis of individual Abusir1630

Michael Francken, Patrick Eppenberger

Material

The mummified head consists of a human cranium, the left half of the mandible, and several cervical vertebrae. Desiccated soft tissue, bandages, and the embalming substance are still attached to the lower half of the cranium and the viscerocranium including the nasal cavity and the frontal bone. The cranium appears undamaged and complete. In contrast, the mandible is unarticulated and only the left half, starting from the alveoli of the left canine, is preserved. Only the articulated head of the mandible remained of the right half. The only present complete tooth is the upper left third molar, while many root apices remained in the mostly open alveoles. Based on this observation, it is apparent that most of the teeth were lost post-mortem. In contrast, the lower-left molars were lost antemortem, presenting a resorbed and smooth alveolar margin. The first three cervical vertebrae are still articulated with the cranial base and covered by dried soft tissue, while another two cervical vertebrae (4th and 5th) are separated. All vertebrae are well preserved and complete except for the 5th cervical vertebra. Here, the left articular process has been removed for DNA-sampling.

Methods

For the osteological examination, we used standard osteological procedures and recording forms [99,128] for the detailed description and study of preservation, age at death, sex, stature, anatomical variants, and pathologies.

While Abusir1630 consists of cranial and cervical remains only, we based our age at death estimations on methods evaluating dental maturation [100,104], and the fusion of cranial sutures [101,102] to assess age at death.

We estimated sex through an evaluation of sexually dimorphic skeletal traits of the skull. According to standard recommendations [99,101,103,104], we assessed the expression of following cranial characteristics, provided that skeletal preservation was sufficient: glabella, superciliary arch, zygomatic and mastoid processes, occipital protuberance, nuchal plane, mentum, and gonion.

In an explorative manner, we identified and recorded skeletal and dental pathologies, and examined the skeletal remains for bone remodeling, signs of inflammation, trauma, lytic defects, other pathological abnormalities, as well as degenerative joint diseases (DJD) [105,106,129].

Age at Death and Sex determination

Considering the dental development of Abusir1630, the individual’s skeletal age was older than 23 years [100]. The present third molar was erupted, in occlusion and fully developed. It is not possible to assess the dental wear of the molar, because a substance, presumably some sort of the embalming substance, covers most of the crown. The ectocranial sutures are partially fused, corresponding to age at death between 30-40 years [99]. Summarizing the recorded data, the estimated skeletal age of Abusir1630 ranges between 30 to 40 years.

Good skeletal preservation allowed a sex estimation using morphological features of the skull. Several indicators of the cranium, including the superciliary arch, mastoid processes, the form of the orbita, the absence of the frontal and parietal tuber, the form of the zygomatic bone, and the expression of the marginal tubercle are all consistent with male morphology [99,101]. The

supraorbital margin appears female, and the form of glabella and gonion are indifferent [99]. Thus, we classified Abusir1630 as a possible male.

Degenerative joint diseases

Degenerative alterations of the joints are frequently present in ancient human remains, especially in skeletons of individuals of advanced age. The term osteoarthritis or degenerative joint disease (DJD) refers to alterations to the skeletal structure of a joint arising from an initial damage of the articular cartilage, which can result from a variety of factors over the course of life, including biomechanical stress, trauma or endocrine disorders [105,129]. Since only the skull and some cervical vertebrae were left for the examination, the assessment was limited to the left temporomandibular joint and the joint surfaces of the available vertebrae. However, the assessable joints were free of attrition, remodeling, or eburnation.

Pathologies

The examination of the skeletal and dental remains revealed no distinct pathologies. Minor porosities were identified on the occipital bone close to the lambda. The origin of these porosities is not evident, they generally are indicators for a porotic hyperostosis, a morphological characteristic not connected to specific diseases [129] but has been frequently related to malnutrition, iron deficiency, or anemia [130,131].

In light of potential infection with leprosy, it is essential to examine the cranium for specific alterations. The nasal and oral cavities are of greater importance since frequent rhinomaxillary leprous alterations resulting from direct bacterial involvement can be observed in addition to skeletal mutilations of the hands and feet. At later stages of leprosy, alterations to the appendages result from secondary infections after necrotizing of the overlying soft tissues [132].

The nasal cavity of Abusir1630 is filled with bandages and covered with embalming substances, preventing direct visual examination. A closer look at the remaining soft tissues of the palate revealed a small oval lesion (1 x 2 cm) in the middle of the palate. The cause and time-of-formation of this lesion could not be determined, but the underlying palatine bone seems intact. A clinical CT scan (Figure S5) confirmed that the nasal cavity is filled with bandages, but was otherwise inconclusive. For further radiological evaluation of the skeletal and cartilaginous structures of the midface, a clinical high-resolution spiral CT scan was performed on a dedicated breast CT scanner (“nu:view”, AB-CT - Advanced Breast-CT GmbH, Erlangen, Germany; field of measurement: Ø 200 mm x 160 mm, tube voltage: 60 kV, tube current: 125 mA, isotropic voxel size: 0.15 mm) at the University Hospital of Zürich. This scan showed very well-preserved bony structures of the nose including the nasal septum as well as partly preserved cartilaginous structures of the nose, including the outer superior nasal septum, parts of the lateral procesus, and parts of the cartilagines alaris majores. Inside the nasal cavity, a very fine textile could be identified in addition to a resinous, crumbly substance. Despite this resolution, direct indications for rhinomaxillary leprous alterations could not be found.

Note 3: Analysis of the HBV data set

Judith Neukamm, Martyna Molak

The estimation of the divergence time was performed (Figure S9C), since the analysis of the HBV dataset yielded a temporal signal. Excluding the newly sequenced strains Abusir1543 from the analysis also does not change the results (Figure S10 and S11), therefore Abusir1543 does not seem to be biasing the timescale estimation. The dataset consists of all modern and ancient HBV strains listed in Table S7, including the newly sequenced strain Abusir1543, dated to 54-124 cal C.E. The data favors an uncorrelated log-normal relaxed molecular clock and a Bayesian Skyline model for variable population size, corroborating a previous study on ancient HBV [48]. Our analysis yields that the tMRCA is estimated to 8,923 B.C.E. (10,942 y (8,525 – 15,576 y 95% HPD)) and thus matching previous results [48]. The tMRCA of Abusir1543 and genotypes A1 and A3, to which Abusir1543 is basal, is estimated to 448 B.C.E. (2,467 y (2,076 - 3,021 y 95% HPD)). The mean clock rate is 1.30 x 10^-5 substitutions per site per year (95% HPD interval: 9.99 x 10^-6 - 1.61 x 10^-5) under this model, confirming previous results [48]. Furthermore, the addition of the oldest genomes so far [47] does not seem to have an influence on the dating of the tree. However, due to recombination events occurring in the HBV genome, the results have to be treated with care. Recombination leads to a mosaic sequence in which the different segments are derived from different ancestor sequences (MRCAs), called parents. As recombination is known within the HBV genome [51], we also tested the newly sequenced strain. In this analysis, two parents were detected for Abusir1543, namely the 4,000-year-old strain RISE254 from Hungary as a minor parent and the strain extracted from an Italian mummified individuals (genotype D), dated to 1509-1629 C.E., as a major parent. As the direct

recombination of these strains forming Abusir1543 is very unlikely, it probably has been ancestral strains of the two strains 'Italian Mummy' and RISE254 who were involved in the recombination.

The varying positioning of the clades using different approaches (maximum likelihood, maximum parsimony, and Bayesian, (Figure S9A-C, [48])) and the low support values of the Maximum likelihood and Maximum parsimony tree indicate an uncertainty in the phylogenetic reconstruction of the data set. The highest support values could be reached using a Bayesian approach. So far, there are no methods available to perform a more reliable analysis and mask putative recombining regions, for example, as done for other pathogens showing recombination [83].

Note 4: Assessment of mitochondrial DNA of individual Abusir1519

Judith Neukamm

As no comparative human mitochondrial genome was available for individual Abusir1519, we reconstructed it for an authenticity support and retrieved 87.94% of the human mitochondrial genome based on 3,579 reads with a coverage of at least 5X and a damage profile of 10.1% to 10.8% (Table S10), suggesting the ancient origin of the reads. Next, contamination was calculated using schmutzi [133], resulting in 1% of modern-day human contamination. The haplogroup N1a1a3 was determined using HaploGrep2 v2.1.19 [134,135]. Having the mutations at positions 16266, 16262, and 16172, the macro-haplogroup can be assigned to the African branch [136].

Figures

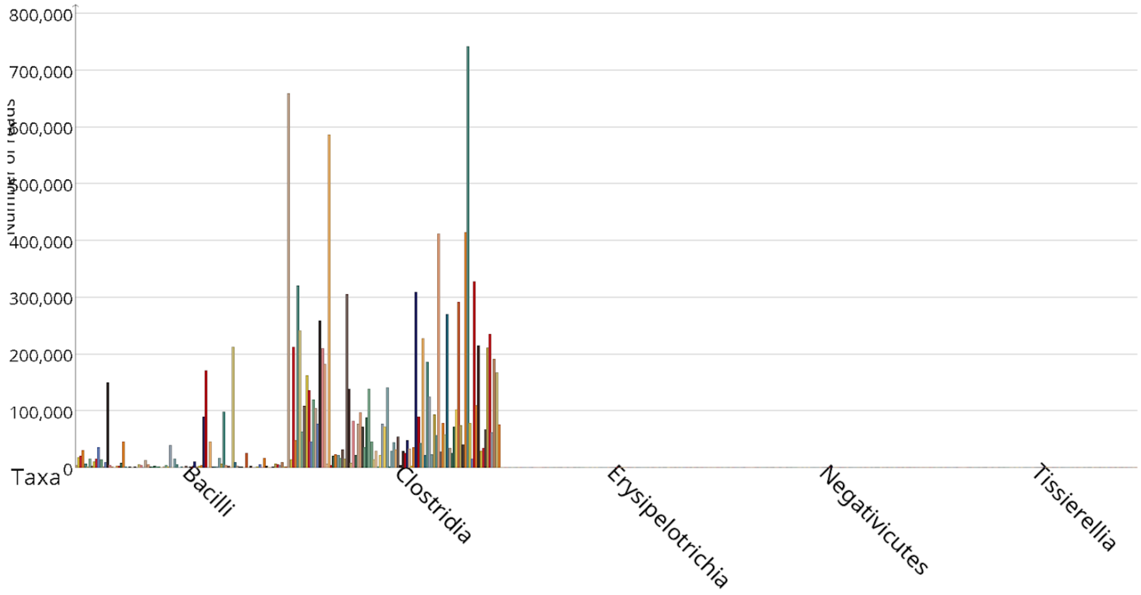

Figure S1: Number of reads mapping to Firmicutes. Most reads are mapped to Clostridia and Bacilli.

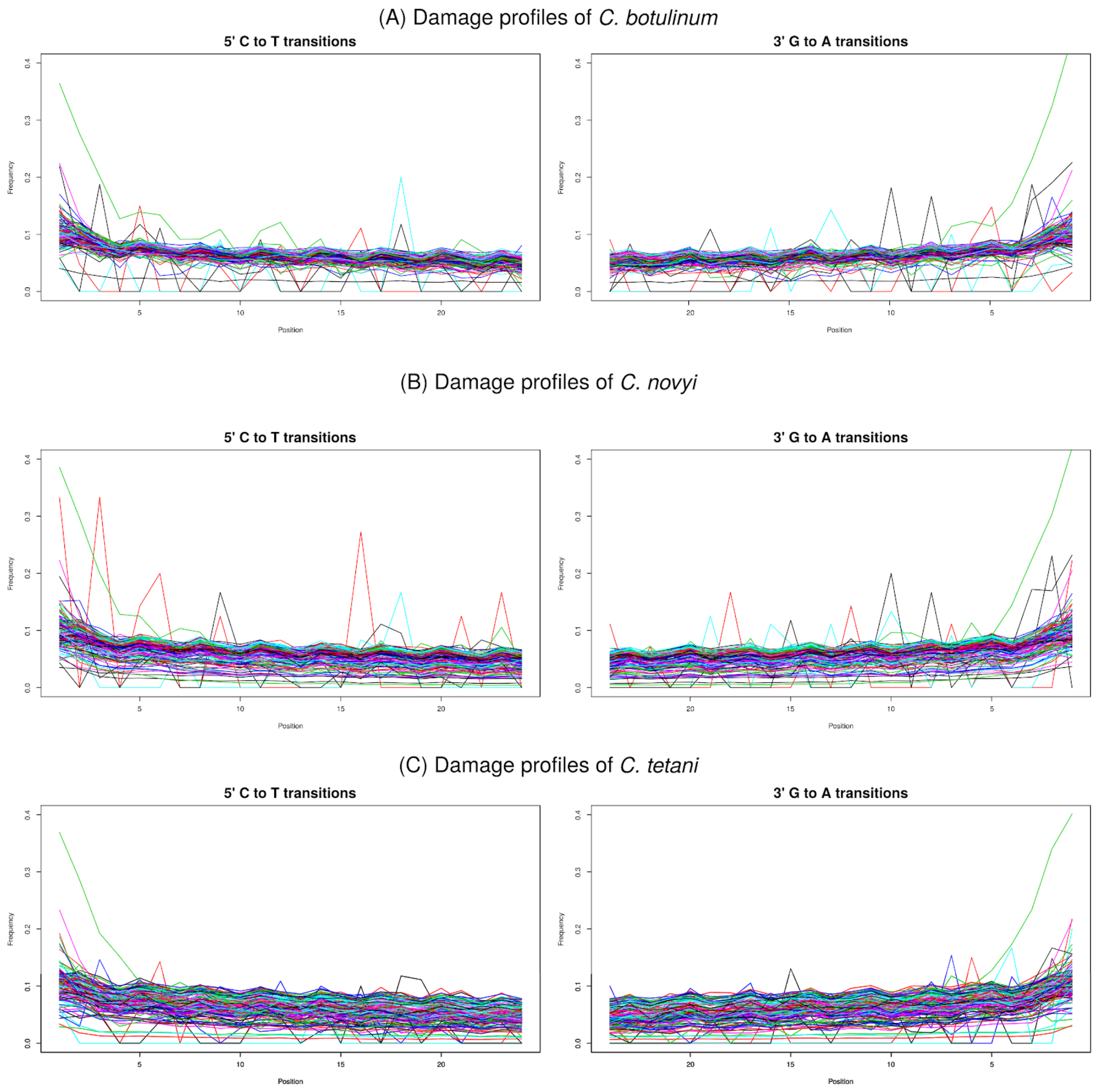

Figure S2: Combined damage profiles for Clostridia. Combined damage profiles of reads mapping to (A) *Clostridium tetani*, (B) *Clostridium botulinum*, and (C) *Clostridium novyi* NT. Each line represents one sample.

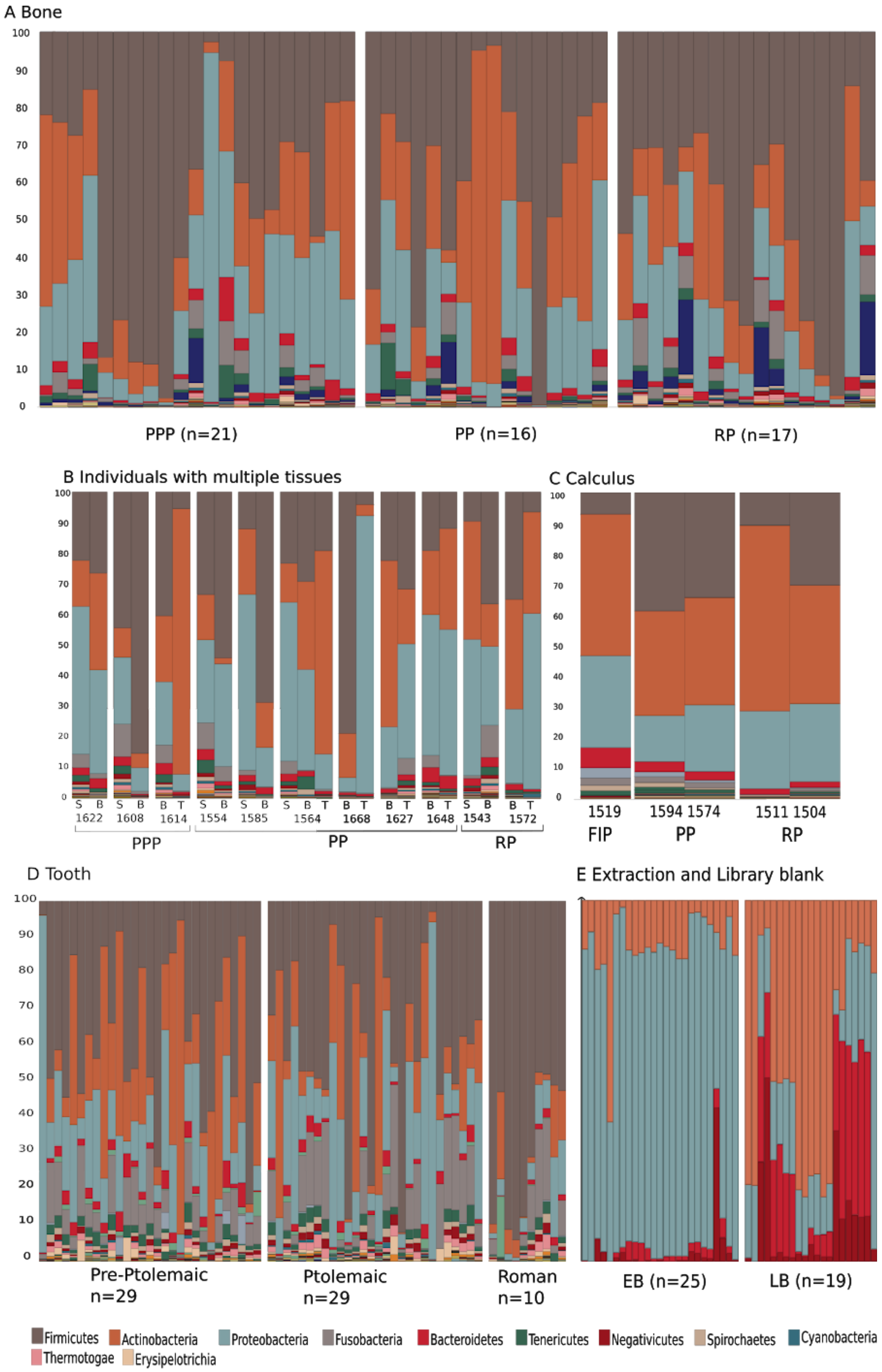
A Bone
PPP (n=21)
PP (n=16)
RP (n=17)
B Individuals with multiple tissues
S B 1622
S B 1608
B T 1614
S B 1554
S B 1585
S B T 1564
B T 1668
B T 1627
B T 1648
S B 1543
B T 1572
PPP
PP
RP
C Calculus
1519
FIP
1594 1574
PP
1511 1504
RP
D Tooth
Pre-Ptolemaic n=29
Ptolemaic n=29
Roman n=10
E Extraction and Library blank
EB (n=25)
LB (n=19)
Firmicutes
Actinobacteria
Proteobacteria
Fusobacteria
Bacteroidetes
Tenericutes
Negativicutes
Spirochaetes
Cyanobacteria
Thermotogae
Erysipelotrichia

Figure S3: Metagenomic composition of all samples. Comparison of bacterial and metagenomic composition of all samples, tissues and time periods: First Intermediate (FIP), pre-Ptolemaic (PPP), Ptolemaic (PP) and Roman period (RP). The individual IDs Abusir<ID> in Figure B and C are shortened to <ID> for better readability. (A) The bacterial composition of all bone samples. The numbers indicate the number of samples per time period. (B) Comparison of metagenomic composition between different tissues from one individual. The letter ‘s’ for soft tissue, ‘b’ for bone, and ‘t’ for the teeth are used to distinguish between the tissue types. (C) Comparison of the metagenomic composition of all calculus samples. (D) Comparison of the metagenomic composition of all tooth samples over all time periods (calculus not included) (E) Metagenomic composition of all library and extraction blanks.

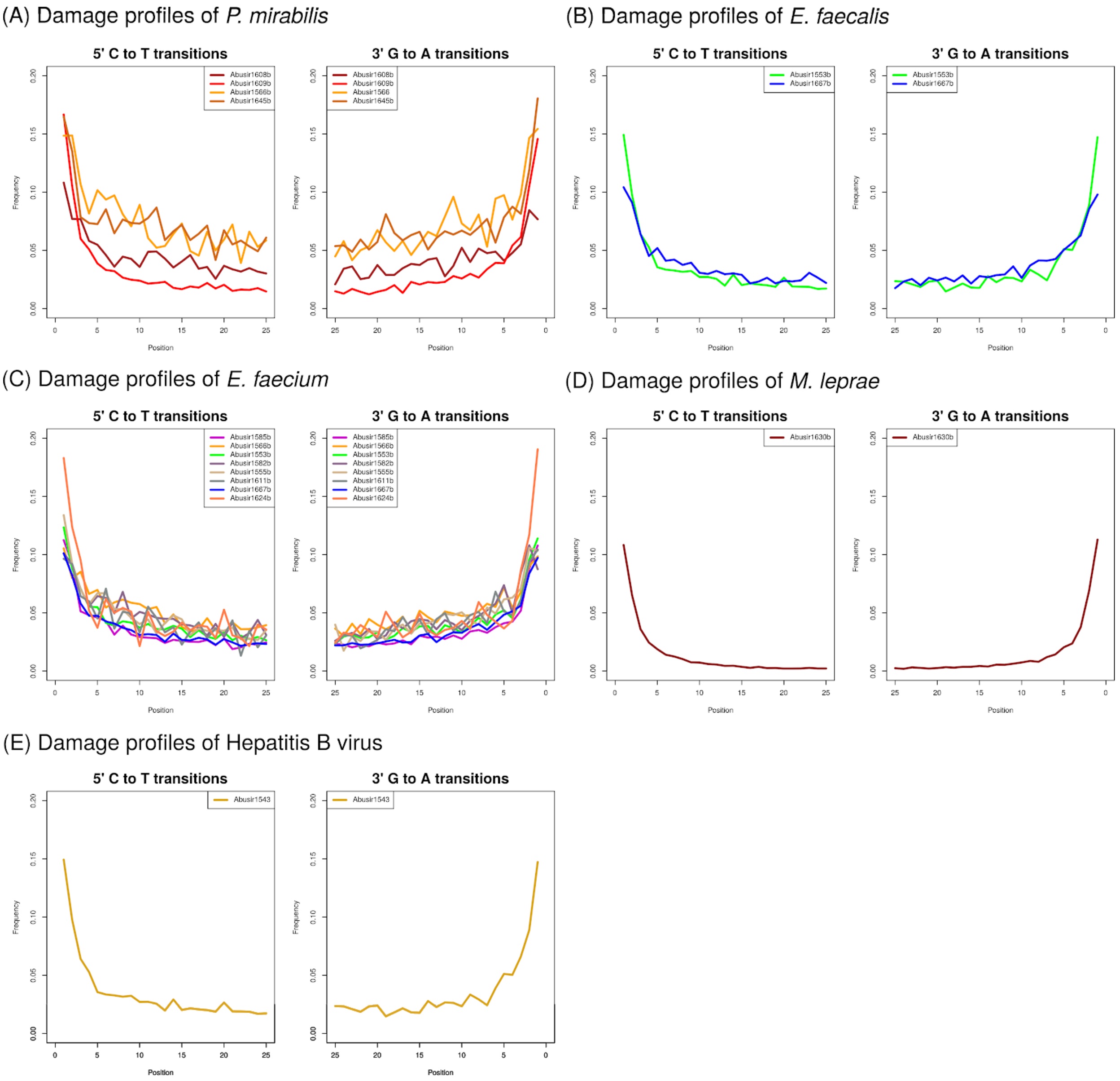

Figure S4: Combined damage profiles of pathogens identified in bone samples. Damage profile of reads mapping to (A) *Proteus mirabilis*, (B) *Enterococcus faecalis*, (C) *Enterococcus faecium*, (D) *Mycobacterium leprae* (sample Abusir1630b), and (E) hepatitis B virus (combined libraries of individual Abusir1543).

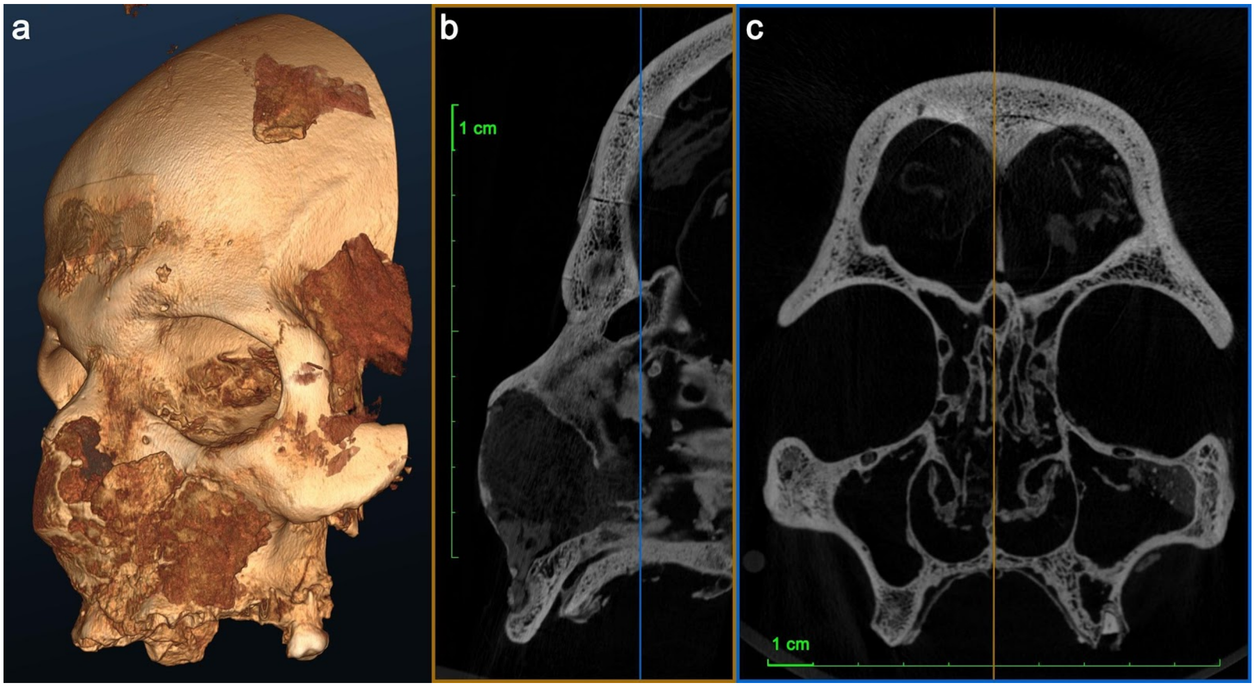

Figure S5: Anthropological analysis of individual Abusir1630. Images of a high-resolution spiral CT of the skeletal and cartilaginous structures of the midface of individual Abusir1630 performed on a dedicated breast CT scanner. (A) Volume rendering of the scanned section of the skull showing skeletal and partially preserved soft tissues. (B) Sagittal section through the nasal septum showing well preserved bony and cartilaginous structures of the nose. (C) Coronary section through the facial skull on which the bony palate, the nasal septum and on both sides the middle and lower turbinates are well distinguishable.

A

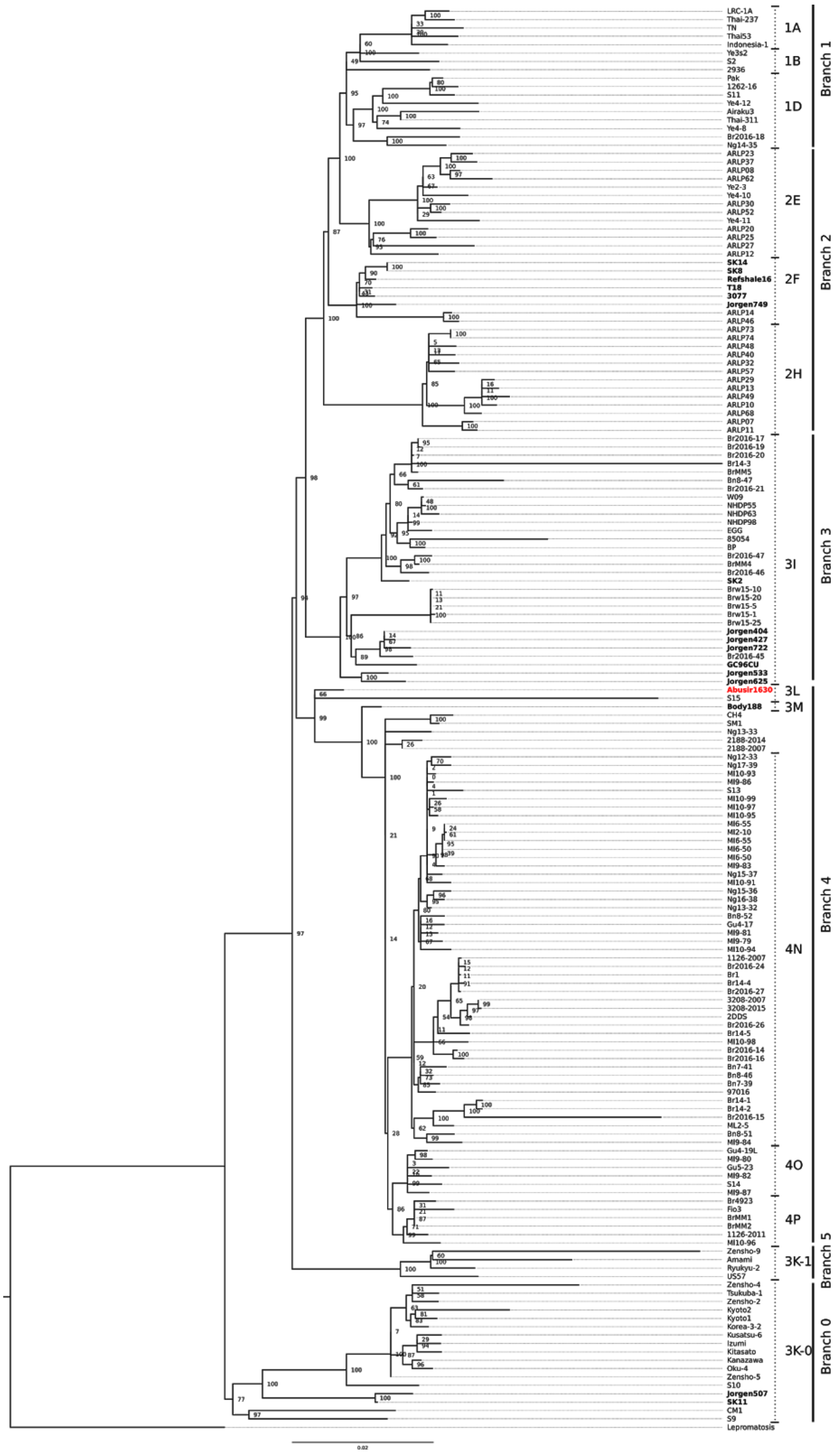

LRC-1A
Thai-237
TN
Thai53
Indonesia-1
Ye3s2
S2
2936
Pak
1262-16
S11
Ye4-12
Airaku3
Thai-311
Ye4-8
Br2016-18
Ng14-35
ARLP23
ARLP37
ARLP08
ARLP62
Ye2-3
Ye4-10
ARLP30
ARLP52
Ye4-11
ARLP20
ARLP25
ARLP27
ARLP12
SK14
SK8
Refshale16
T18
3077
Jorgen749
ARLP14
ARLP46
ARLP73
ARLP74
ARLP48
ARLP40
ARLP32
ARLP57
ARLP29
ARLP13
ARLP49
ARLP10
ARLP68
ARLP07
ARLP11
Br2016-17
Br2016-19
Br2016-20
Br14-3
BrMM5
Bn8-47
Br2016-21
W09
NHDP55
NHDP63
NHDP98
EGG
85054
BP
Br2016-47
BrMM4
Br2016-46
SK2
Brw15-10
Brw15-20
Brw15-5
Brw15-1
Brw15-25
Jorgen404
Jorgen427
Jorgen722
Br2016-45
GC96CU
Jorgen533
Jorgen625
Abusir1630
S15
Body188
CH4
SM1
Ng13-33
2188-2014
2188-2007
Ng12-33
Ng17-39
Ml10-93
Ml9-86
S13
Ml10-99
Ml10-97
Ml10-95
Ml6-55
Ml2-10
Ml6-55
Ml6-50
Ml6-50
Ml9-83
Ng15-37
Ml10-91
Ng15-36
Ng16-38
Ng13-32
Bn8-52
Gu4-17
Ml9-81
Ml9-79
Ml10-94
1126-2007
Br2016-24
Br1
Br14-4
Br2016-27
3208-2007
3208-2015
2DDS
Br2016-26
Br14-5
Ml10-98
Br2016-14
Br2016-16
Bn7-41
Bn8-46
Bn7-39
97016
Br14-1
Br14-2
Br2016-15
ML2-5
Bn8-51
Ml9-84
Gu4-19L
Ml9-80
Gu5-23
Ml9-82
S14
Ml9-87
Br4923
Fio3
BrMM1
BrMM2
1126-2011
Ml10-96
Zensho-9
Amami
Ryukyu-2
US57
Zensho-4
Tsukuba-1
Zensho-2
Kyoto2
Kyoto1
Korea-3-2
Kusatsu-6
Izumi
Kitasato
Kanazawa
Oku-4
Zensho-5
S10
Jorgen507
SK11
CM1
S9
Lepromatosis
1A
1B
1D
2E
2F
2H
3I
3L
3M
4N
4O
4P
3K-1
3K-0
Branch 1
Branch 2
Branch 3
Branch 4
Branch 5
Branch 0
0.02

B

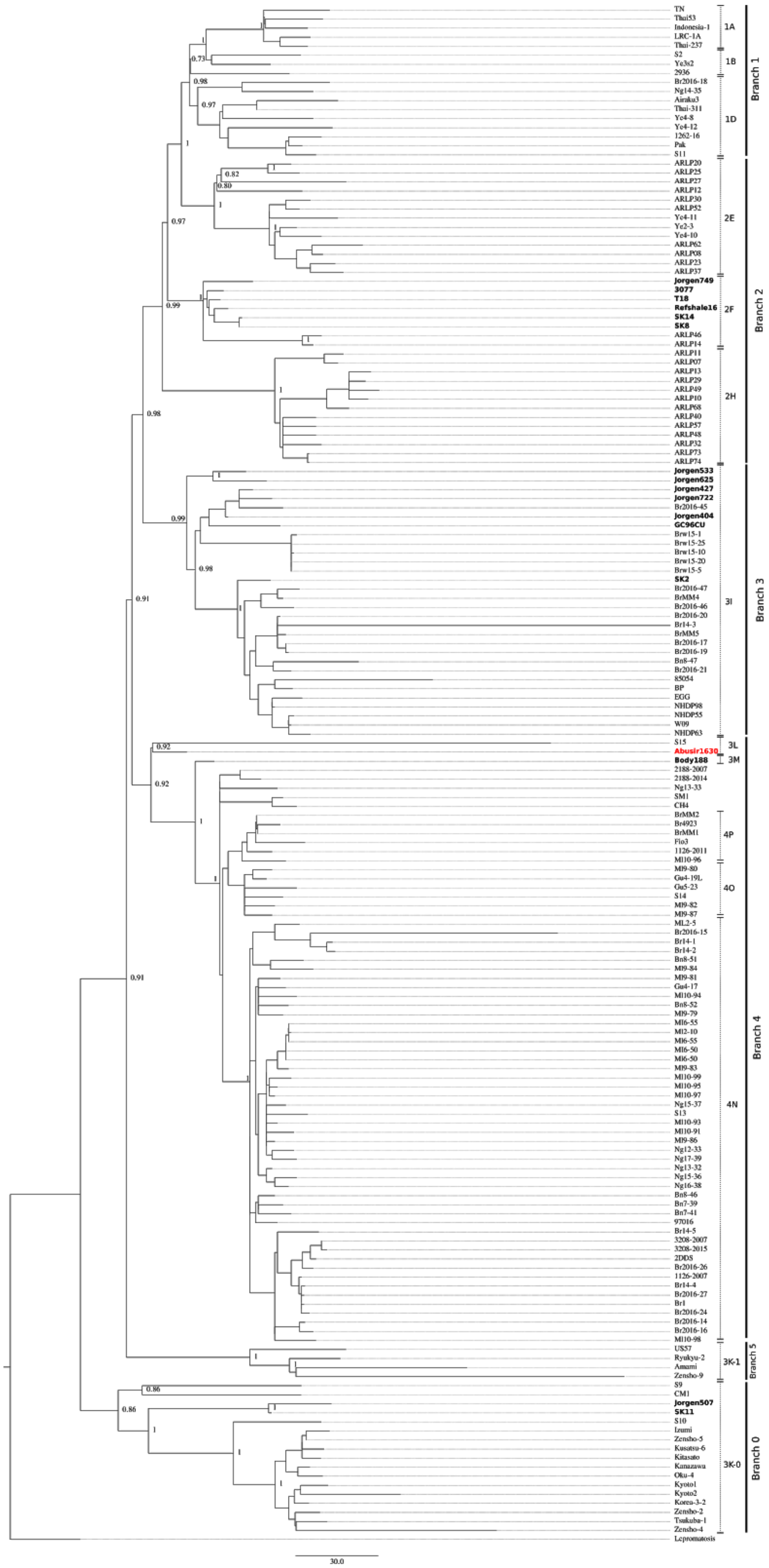
TN
Thai53
Indonesia-1
LRC-1A
Thai-237
S2
Ye3s2
2936
Br2016-18
Ng14-35
Airaku3
Thai-311
Ye4-8
Ye4-12
1262-16
Pak
S11
ARLP20
ARLP25
ARLP27
ARLP12
ARLP30
ARLP52
Ye4-11
Ye2-3
Ye4-10
ARLP62
ARLP08
ARLP23
ARLP37
Jorgen749
3077
T18
Refshale16
SK14
SK8
ARLP46
ARLP14
ARLP11
ARLP07
ARLP13
ARLP29
ARLP49
ARLP10
ARLP68
ARLP40
ARLP57
ARLP48
ARLP32
ARLP73
ARLP74
Jorgen533
Jorgen625
Jorgen427
Jorgen722
Br2016-45
Jorgen404
GC96CU
Brw15-1
Brw15-25
Brw15-10
Brw15-20
Brw15-5
SK2
Br2016-47
BrMM4
Br2016-46
Br2016-20
Br14-3
BrMM5
Br2016-17
Br2016-19
Bn8-47
Br2016-21
85054
BP
EGG
NHDP98
NHDP55
W09
NHDP63
S15
Abusir1630
Body188
2188-2007
2188-2014
Ng13-33
SM1
CH4
BrMM2
Br4923
BrMM1
Fio3
1126-2011
Ml10-96
Ml9-80
Gu4-19L
Gu5-23
S14
Ml9-82
Ml9-87
ML2-5
Br2016-15
Br14-1
Br14-2
Bn8-51
Ml9-84
Ml9-81
Gu4-17
Ml10-94
Bn8-52
Ml9-79
Ml6-55
Ml2-10
Ml6-55
Ml6-50
Ml6-50
Ml9-83
Ml10-99
Ml10-95
Ml10-97
Ng15-37
S13
Ml10-93
Ml10-91
Ml9-86
Ng12-33
Ng17-39
Ng13-32
Ng15-36
Ng16-38
Bn8-46
Bn7-39
Bn7-41
97016
Br14-5
3208-2007
3208-2015
2DDS
Br2016-26
1126-2007
Br14-4
Br2016-27
Br1
Br2016-24
Br2016-14
Br2016-16
Ml10-98
US57
Ryukyu-2
Amami
Zensho-9
S9
CM1
Jorgen507
SK11
S10
Izumi
Zensho-5
Kusatsu-6
Kitasato
Kanazawa
Oku-4
Kyoto1
Kyoto2
Korea-3-2
Zensho-2
Tsukuba-1
Zensho-4
Lepromatosis
1A
1B
1D
2E
2F
2H
3I
3L
3M
4P
4O
4N
3K-1
3K-0
Branch 1
Branch 2
Branch 3
Branch 4
Branch 5
Branch 0
30.0

C

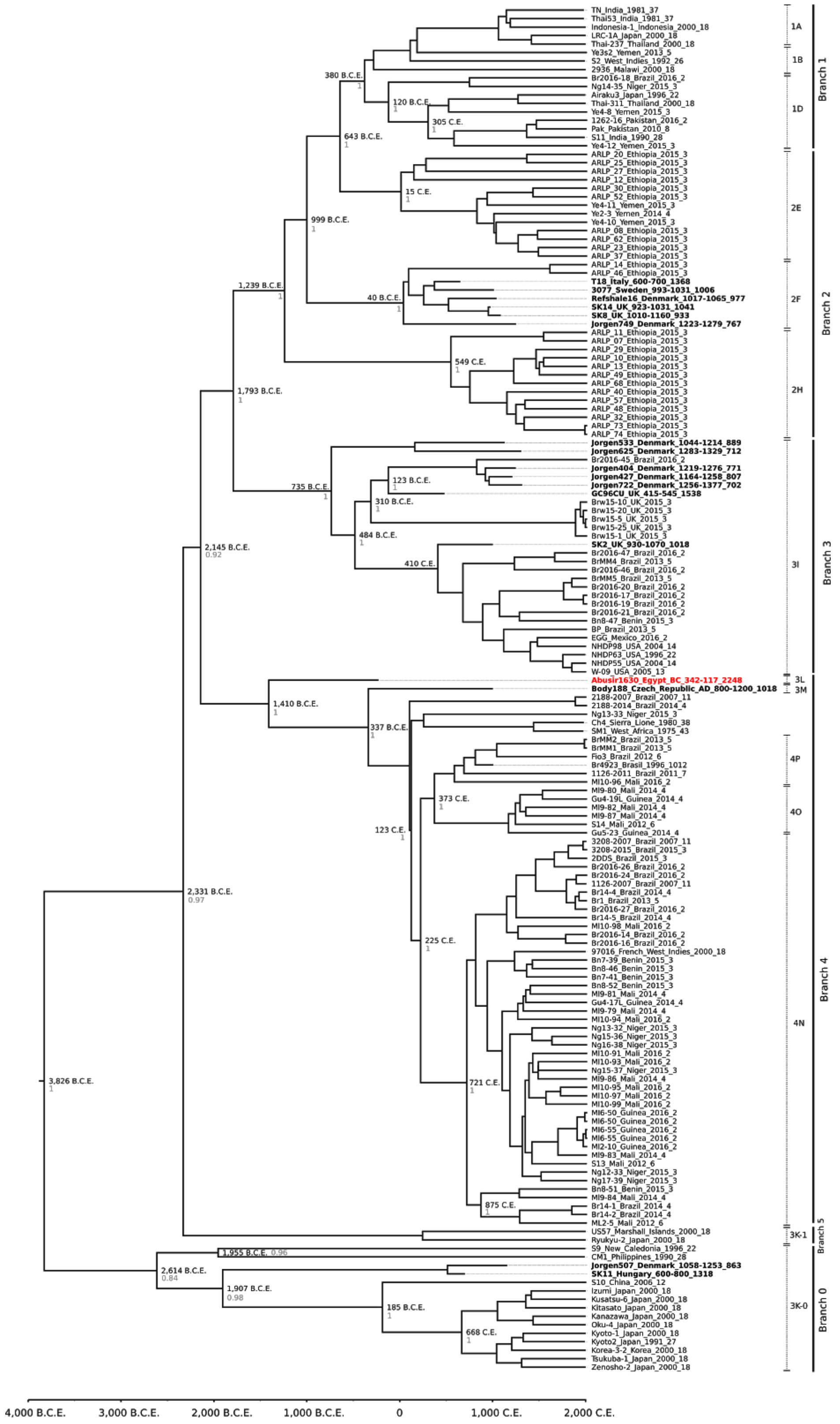

TN_India_1981_37
Thai53_India_1981_37
Indonesia-1_Indonesia_2000_18
LRC-1A_Japan_2000_18
Thai-237_Thailand_2000_18
Ye3s2_Yemen_2013_5
S2_West_Indies_1992_26
2936_Malawi_2000_18
Br2016-18_Brazil_2016_2
Ng14-35_Niger_2015_3
Airaku3_Japan_1996_22
Thai-311_Thailand_2000_18
Ye4-8_Yemen_2015_3
1262-16_Pakistan_2016_2
Pak_Pakistan_2010_8
S11_India_1990_28
Ye4-12_Yemen_2015_3
ARLP_20_Ethiopia_2015_3
ARLP_25_Ethiopia_2015_3
ARLP_27_Ethiopia_2015_3
ARLP_12_Ethiopia_2015_3
ARLP_30_Ethiopia_2015_3
ARLP_52_Ethiopia_2015_3
Ye4-11_Yemen_2015_3
Ye2-3_Yemen_2014_4
Ye4-10_Yemen_2015_3
ARLP_08_Ethiopia_2015_3
ARLP_62_Ethiopia_2015_3
ARLP_23_Ethiopia_2015_3
ARLP_37_Ethiopia_2015_3
ARLP_14_Ethiopia_2015_3
ARLP_46_Ethiopia_2015_3
T18_Italy_600-700_1368
3077_Sweden_993-1031_1006
Refshale16_Denmark_1017-1065_977
SK14_UK_923-1031_1041
SK8_UK_1010-1160_933
Jorgen749_Denmark_1223-1279_767
ARLP_11_Ethiopia_2015_3
ARLP_07_Ethiopia_2015_3
ARLP_29_Ethiopia_2015_3
ARLP_10_Ethiopia_2015_3
ARLP_13_Ethiopia_2015_3
ARLP_49_Ethiopia_2015_3
ARLP_68_Ethiopia_2015_3
ARLP_40_Ethiopia_2015_3
ARLP_57_Ethiopia_2015_3
ARLP_48_Ethiopia_2015_3
ARLP_32_Ethiopia_2015_3
ARLP_73_Ethiopia_2015_3
ARLP_74_Ethiopia_2015_3
Jorgen533_Denmark_1044-1214_889
Jorgen625_Denmark_1283-1329_712
Br2016-45_Brazil_2016_2
Jorgen404_Denmark_1219-1276_771
Jorgen427_Denmark_1164-1258_807
Jorgen722_Denmark_1256-1377_702
GC96CU_UK_415-545_1538
Brw15-10_UK_2015_3
Brw15-20_UK_2015_3
Brw15-5_UK_2015_3
Brw15-25_UK_2015_3
Brw15-1_UK_2015_3
SK2_UK_930-1070_1018
Br2016-47_Brazil_2016_2
BrMM4_Brazil_2013_5
Br2016-46_Brazil_2016_2
BrMM5_Brazil_2013_5
Br2016-20_Brazil_2016_2
Br2016-17_Brazil_2016_2
Br2016-19_Brazil_2016_2
Br2016-21_Brazil_2016_2
Bn8-47_Benin_2015_3
BP_Brazil_2013_5
EGG_Mexico_2016_2
NHDP98_USA_2004_14
NHDP63_USA_1996_22
NHDP55_USA_2004_14
W-09_USA_2005_13
Abusir1630_Egypt_BC_342-117_2248
Body188_Czech_Republic_AD_800-1200_1018
2188-2007_Brazil_2007_11
2188-2014_Brazil_2014_4
Ng13-33_Niger_2015_3
Ch4_Sierra_Lione_1980_38
SM1_West_Africa_1975_43
BrMM2_Brazil_2013_5
BrMM1_Brazil_2013_5
Fio3_Brazil_2012_6
Br4923_Brasil_1996_1012
1126-2011_Brazil_2011_7
Ml10-96_Mali_2016_2
Ml9-80_Mali_2014_4
Gu4-19L_Guinea_2014_4
Ml9-82_Mali_2014_4
Ml9-87_Mali_2014_4
S14_Mali_2012_6
Gu5-23_Guinea_2014_4
3208-2007_Brazil_2007_11
3208-2015_Brazil_2015_3
2DDS_Brazil_2015_3
Br2016-26_Brazil_2016_2
Br2016-24_Brazil_2016_2
1126-2007_Brazil_2007_11
Br14-4_Brazil_2014_4
Br1_Brazil_2013_5
Br2016-27_Brazil_2016_2
Br14-5_Brazil_2014_4
Ml10-98_Mali_2016_2
Br2016-14_Brazil_2016_2
Br2016-16_Brazil_2016_2
97016_French_West_Indies_2000_18
Bn7-39_Benin_2015_3
Bn8-46_Benin_2015_3
Bn7-41_Benin_2015_3
Bn8-52_Benin_2015_3
Ml9-81_Mali_2014_4
Gu4-17L_Guinea_2014_4
Ml9-79_Mali_2014_4
Ml10-94_Mali_2016_2
Ng13-32_Niger_2015_3
Ng15-36_Niger_2015_3
Ng16-38_Niger_2015_3
Ml10-91_Mali_2016_2
Ml10-93_Mali_2016_2
Ng15-37_Niger_2015_3
Ml9-86_Mali_2014_4
Ml10-95_Mali_2016_2
Ml10-97_Mali_2016_2
Ml10-99_Mali_2016_2
Ml6-50_Guinea_2016_2
Ml6-50_Guinea_2016_2
Ml6-55_Guinea_2016_2
Ml6-55_Guinea_2016_2
Ml2-10_Guinea_2016_2
Ml9-83_Mali_2014_4
S13_Mali_2012_6
Ng12-33_Niger_2015_3
Ng17-39_Niger_2015_3
Bn8-51_Benin_2015_3
Ml9-84_Mali_2014_4
Br14-1_Brazil_2014_4
Br14-2_Brazil_2014_4
ML2-5_Mali_2012_6
US57_Marshall_Islands_2000_18
Ryukyu-2_Japan_2000_18
S9_New_Caledonia_1996_22
CM1_Philippines_1990_28
Jorgen507_Denmark_1058-1253_863
SK11_Hungary_600-800_1318
S10_China_2006_12
Izumi_Japan_2000_18
Kusatsu-6_Japan_2000_18
Kitasato_Japan_2000_18
Kanazawa_Japan_2000_18
Oku-4_Japan_2000_18
Kyoto-1_Japan_2000_18
Kyoto2_Japan_1991_27
Korea-3-2_Korea_2000_18
Tsukuba-1_Japan_2000_18
Zenosho-2_Japan_2000_18
380 B.C.E.
120 B.C.E.
305 C.E.
643 B.C.E.
15 C.E.
999 B.C.E.
1,239 B.C.E.
40 B.C.E.
549 C.E.
1,793 B.C.E.
735 B.C.E.
123 B.C.E.
310 B.C.E.
484 B.C.E.
410 C.E.
2,145 B.C.E.
0.92
1,410 B.C.E.
337 B.C.E.
373 C.E.
123 C.E.
2,331 B.C.E.
0.97
225 C.E.
721 C.E.
875 C.E.
3,826 B.C.E.
1,955 B.C.E. 0.96
2,614 B.C.E.
0.84
1,907 B.C.E.
0.98
185 B.C.E.
668 C.E.
1A
1B
1D
2E
2F
2H
3I
3L
3M
4P
4O
4N
3K-1
3K-0
Branch 1
Branch 2
Branch 3
Branch 4
Branch 5
Branch 0
4,000 B.C.E.
3,000 B.C.E.
2,000 B.C.E.
1,000 B.C.E.
0
1,000 C.E.
2,000 C.E.

Figure S6: Phylogenetic trees of *M. leprae* genomes. (A) A Maximum likelihood leprosy tree of all *M. leprae* genomes. Ancient strains are labeled in bold, the newly added strain Abusir1630 is labeled in red. Bootstrap values (1000BS) are given as node labels. (B) Maximum parsimony tree reconstructed from 3,342 informative SNP positions based on the 161 *M. leprae* genomes. The tree is drawn to scale and branch length represents the number of substitutions. *M. lepromatosis* was used as an outgroup. Ancient strains are labeled in bold, the newly added strain Abusir1630 is labeled in red. Bootstrap values (1000BS) are presented as node labels. (C) Dated Bayesian Maximum Clade Credibility tree reconstructed using 3,342 informative SNP positions from 161 *M. leprae* samples (with no outgroup), strict molecular clock and Bayesian Skyline model. Ancient samples are bold, the newly added genome Abusir1630 in red. The node labels are the median divergence times in years B.C.E. and C.E.

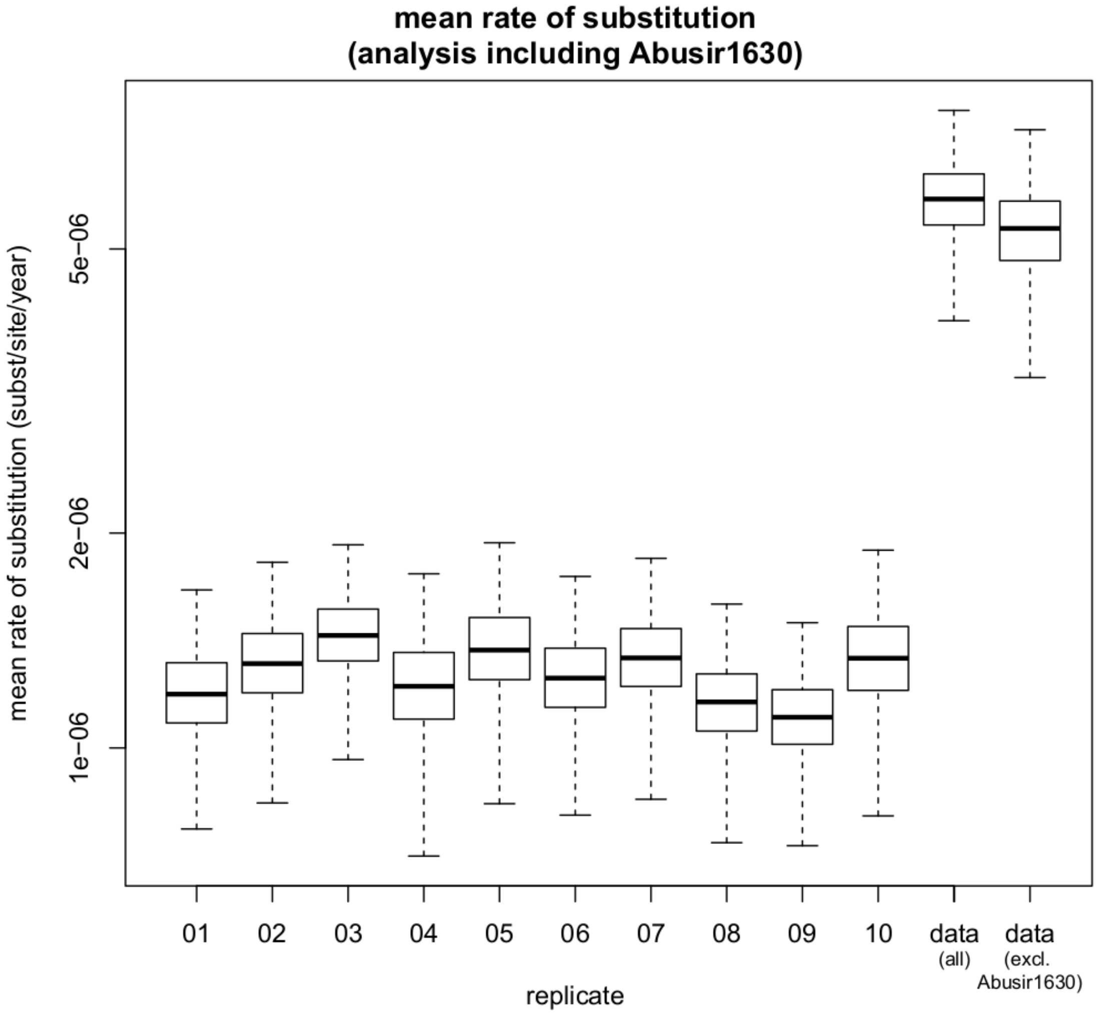

Figure S7: Date Randomization test for the *M. leprae* data set. BEAST analysis was performed for the original data set and ten replicates with randomly reassigned tip calibrations (ages of the samples). The lack of overlap between the timescale parameter estimates (here, the mean rate of nucleotide substitution) indicates a sufficient temporal signal for the molecular clock calibration and time-aware phylogenetic inference. Additionally an analysis excluding strain Abusir1630 was performed to examine its effect on the substitution rate estimate.

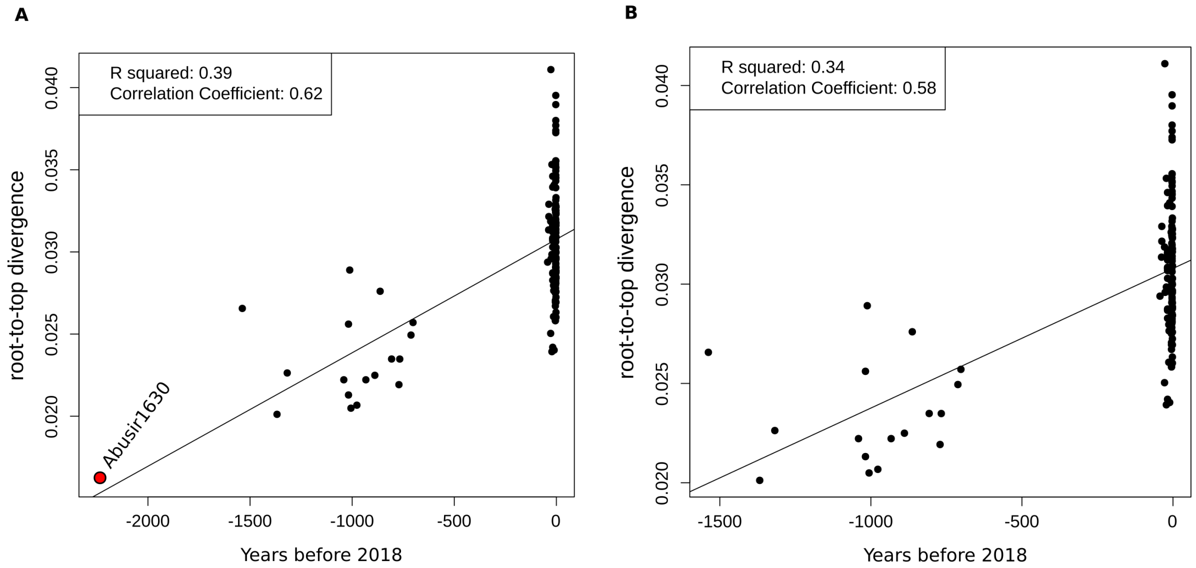

Figure S8: TempEst analysis for the *M. leprae* dataset. Analysis (A) with and (B) without the new strain Abusir1630 (red dot). The plot visualizes the phylogenetic root-to-tip distance relative to sampling time in years before present with the year 2019 as present. Both analyses show an almost identical temporal signal.

A

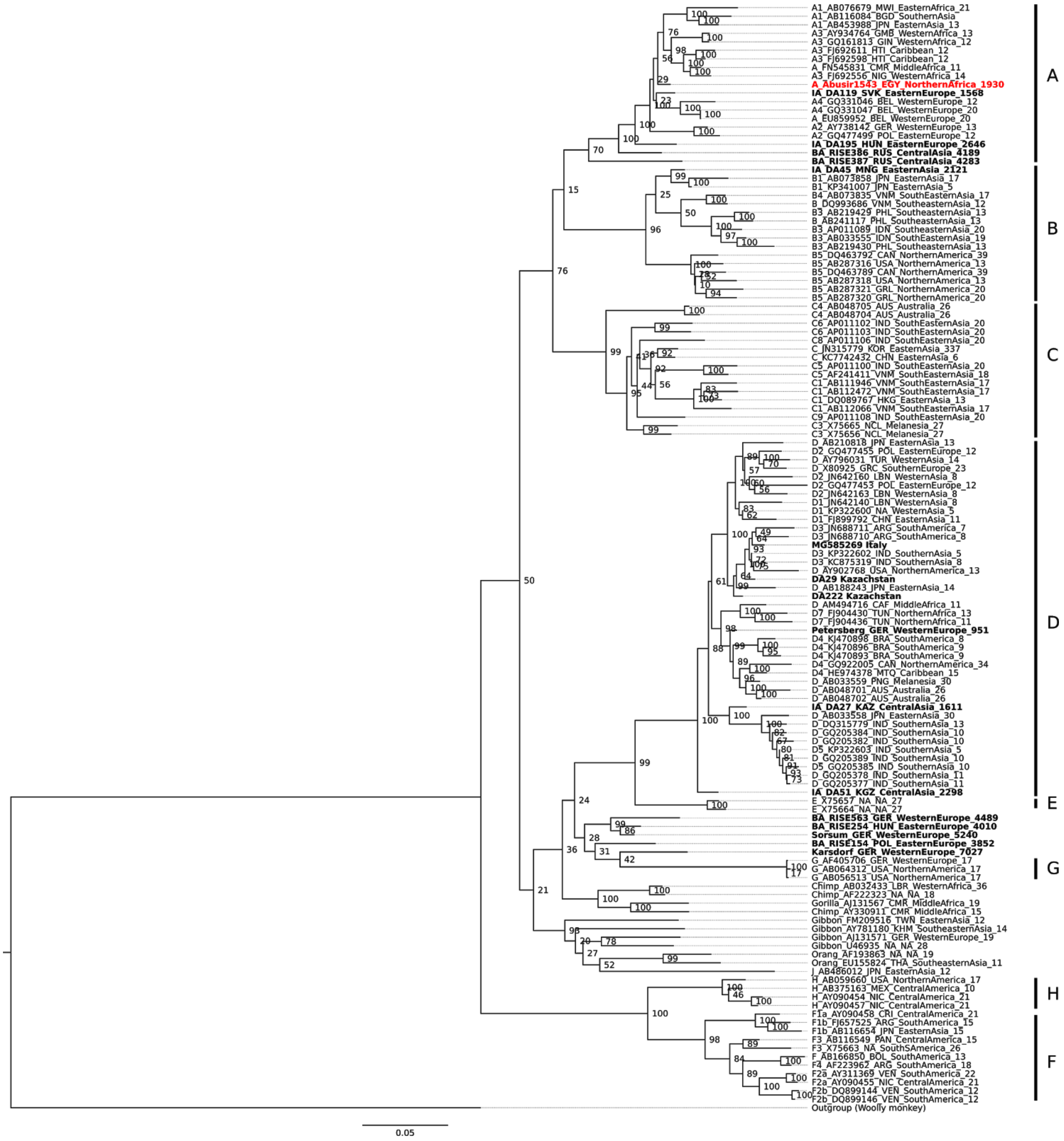

A1_AB076679_MWI_EasternAfrica_21
A1_AB116084_BGD_SouthernAsia
A1_AB453988_JPN_EasternAsia_13
A3_AY934764_GMB_WesternAfrica_13
A3_GQ161813_GIN_WesternAfrica_12
A3_FJ692611_HTI_Caribbean_12
A3_FJ692598_HTI_Caribbean_12
A_FN545831_CMR_MiddleAfrica_11
A3_FJ692556_NIG_WesternAfrica_14
A_Abusir1543_EGY_NorthernAfrica_1930
IA_DA119_SVK_EasternEurope_1568
A4_GQ331046_BEL_WesternEurope_12
A4_GQ331047_BEL_WesternEurope_20
A_EU859952_BEL_WesternEurope_20
A2_AY738142_GER_WesternEurope_13
A2_GQ477499_POL_EasternEurope_12
IA_DA195_HUN_EasternEurope_2646
BA_RISE386_RUS_CentralAsia_4189
BA_RISE387_RUS_CentralAsia_4283
IA_DA45_MNG_EasternAsia_2121
B1_AB073858_JPN_EasternAsia_17
B1_KP341007_JPN_EasternAsia_5
B4_AB073835_VNM_SouthEasternAsia_17
B_DQ993686_VNM_SoutheasternAsia_12
B3_AB219429_PHL_SoutheasternAsia_13
B_AB241117_PHL_SoutheasternAsia_13
B3_AP011089_IDN_SoutheasternAsia_20
B3_AB033555_IDN_SouthEasternAsia_19
B3_AB219430_PHL_SoutheasternAsia_13
B5_DQ463792_CAN_NorthernAmerica_39
B5_AB287316_USA_NorthernAmerica_13
B5_DQ463789_CAN_NorthernAmerica_39
B5_AB287318_USA_NorthernAmerica_13
B5_AB287321_GRL_NorthernAmerica_20
B5_AB287320_GRL_NorthernAmerica_20
C4_AB048705_AUS_Australia_26
C4_AB048704_AUS_Australia_26
C6_AP011102_IND_SouthEasternAsia_20
C6_AP011103_IND_SouthEasternAsia_20
C8_AP011106_IND_SouthEasternAsia_20
C_JN315779_KOR_EasternAsia_337
C_KC7742432_CHN_EasternAsia_6
C5_AP011100_IND_SouthEasternAsia_20
C5_AF241411_VNM_SouthEasternAsia_18
C1_AB111946_VNM_SouthEasternAsia_17
C1_AB112472_VNM_SouthEasternAsia_17
C1_DQ089767_HKG_EasternAsia_13
C1_AB112066_VNM_SouthEasternAsia_17
C9_AP011108_IND_SouthEasternAsia_20
C3_X75665_NCL_Melanesia_27
C3_X75656_NCL_Melanesia_27
D_AB210818_JPN_EasternAsia_13
D2_GQ477455_POL_EasternEurope_12
D_AY796031_TUR_WesternAsia_14
D_X80925_GRC_SouthernEurope_23
D2_JN642160_LBN_WesternAsia_8
D2_GQ477453_POL_EasternEurope_12
D2_JN642163_LBN_WesternAsia_8
D1_JN642140_LBN_WesternAsia_8
D1_KP322600_NA_WesternAsia_5
D1_FJ899792_CHN_EasternAsia_11
D3_JN688711_ARG_SouthAmerica_7
D3_JN688710_ARG_SouthAmerica_8
MG585269 Italy
D3_KP322602_IND_SouthernAsia_5
D3_KC875319_IND_SouthernAsia_8
D_AY902768_USA_NorthernAmerica_13
DA29 Kazachstan
D_AB188243_JPN_EasternAsia_14
DA222 Kazachstan
D_AM494716_CAF_MiddleAfrica_11
D7_FJ904430_TUN_NorthernAfrica_13
D7_FJ904436_TUN_NorthernAfrica_11
Petersberg_GER_WesternEurope_951
D4_KJ470898_BRA_SouthAmerica_8
D4_KJ470896_BRA_SouthAmerica_9
D4_KJ470893_BRA_SouthAmerica_9
D4_GQ922005_CAN_NorthernAmerica_34
D4_HE974378_MTQ_Caribbean_15
D_AB033559_PNG_Melanesia_30
D_AB048701_AUS_Australia_26
D_AB048702_AUS_Australia_26
IA_DA27_KAZ_CentralAsia_1611
D_AB033558_JPN_EasternAsia_30
D_DQ315779_IND_SouthernAsia_13
D_GQ205384_IND_SouthernAsia_10
D_GQ205382_IND_SouthernAsia_10
D5_KP322603_IND_SouthernAsia_5
D_GQ205389_IND_SouthernAsia_10
D5_GQ205385_IND_SouthernAsia_10
D_GQ205378_IND_SouthernAsia_11
D_GQ205377_IND_SouthernAsia_11
IA_DA51_KGZ_CentralAsia_2298
E_X75657_NA_NA_27
E_X75664_NA_NA_27
BA_RISE563_GER_WesternEurope_4489
BA_RISE254_HUN_EasternEurope_4010
Sorsum_GER_WesternEurope_5240
BA_RISE154_POL_EasternEurope_3852
Karsdorf_GER_WesternEurope_7027
G_AF405706_GER_WesternEurope_17
G_AB064312_USA_NorthernAmerica_17
G_AB056513_USA_NorthernAmerica_17
Chimp_AB032433_LBR_WesternAfrica_36
Chimp_AF222323_NA_NA_18
Gorilla_AJ131567_CMR_MiddleAfrica_19
Chimp_AY330911_CMR_MiddleAfrica_15
Gibbon_FM209516_TWN_EasternAsia_12
Gibbon_AY781180_KHM_SoutheasternAsia_14
Gibbon_AJ131571_GER_WesternEurope_19
Gibbon_U46935_NA_NA_28
Orang_AF193863_NA_NA_19
Orang_EU155824_THA_SoutheasternAsia_11
J_AB486012_JPN_EasternAsia_12
H_AB059660_USA_NorthernAmerica_17
H_AB375163_MEX_CentralAmerica_10
H_AY090454_NIC_CentralAmerica_21
H_AY090457_NIC_CentralAmerica_21
F1a_AY090458_CRI_CentralAmerica_21
F1b_FJ657525_ARG_SouthAmerica_15
F1b_AB116654_JPN_EasternAsia_15
F3_AB116549_PAN_CentralAmerica_15
F3_X75663_NA_SouthSAmerica_26
F_AB166850_BOL_SouthAmerica_13
F4_AF223962_ARG_SouthAmerica_18
F2a_AY311369_VEN_SouthAmerica_22
F2a_AY090455_NIC_CentralAmerica_21
F2b_DQ899144_VEN_SouthAmerica_12
F2b_DQ899146_VEN_SouthAmerica_12
Outgroup (Woolly monkey)
A
B
C
D
E
G
H
F
0.05

B

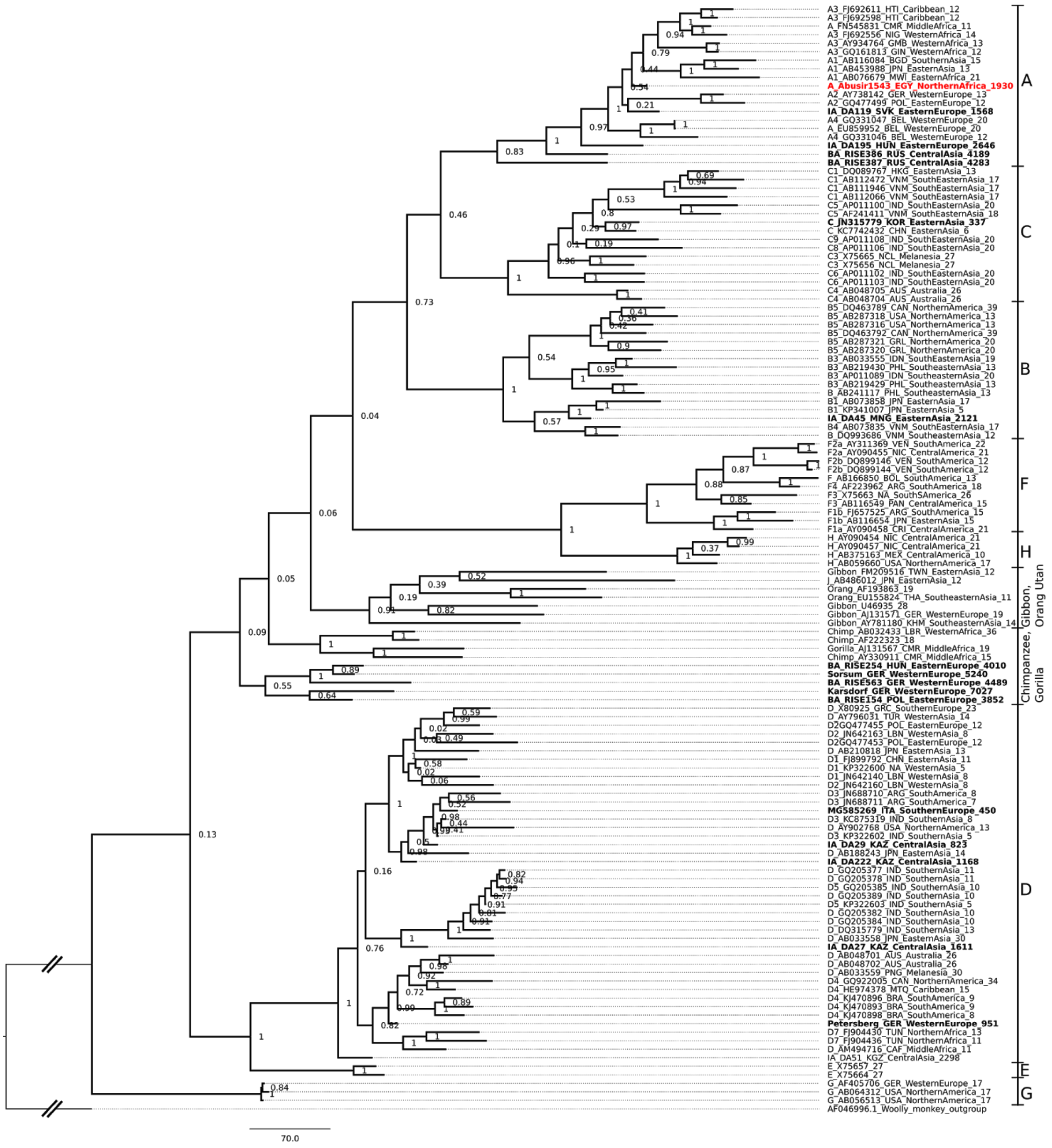
A3_FJ692611_HTI_Caribbean_12
A3_FJ692598_HTI_Caribbean_12
A_FN545831_CMR_MiddleAfrica_11
A3_FJ692556_NIG_WesternAfrica_14
A3_AY934764_GMB_WesternAfrica_13
A3_GQ161813_GIN_WesternAfrica_12
A1_AB116084_BGD_SouthernAsia_15
A1_AB453988_JPN_EasternAsia_13
A1_AB076679_MWI_EasternAfrica_21
A_Abusir1543_EGY_NorthernAfrica_1930
A2_AY738142_GER_WesternEurope_13
A2_GQ477499_POL_EasternEurope_12
IA_DA119_SVK_EasternEurope_1568
A4_GQ331047_BEL_WesternEurope_20
A_EU859952_BEL_WesternEurope_20
A4_GQ331046_BEL_WesternEurope_12
IA_DA195_HUN_EasternEurope_2646
BA_RISE386_RUS_CentralAsia_4189
BA_RISE387_RUS_CentralAsia_4283
C1_DQ089767_HKG_EasternAsia_13
C1_AB112472_VNM_SouthEasternAsia_17
C1_AB111946_VNM_SouthEasternAsia_17
C1_AB112066_VNM_SouthEasternAsia_17
C5_AP011100_IND_SouthEasternAsia_20
C5_AF241411_VNM_SouthEasternAsia_18
C_JN315779_KOR_EasternAsia_337
C_KC7742432_CHN_EasternAsia_6
C9_AP011108_IND_SouthEasternAsia_20
C8_AP011106_IND_SouthEasternAsia_20
C3_X75665_NCL_Melanesia_27
C3_X75656_NCL_Melanesia_27
C6_AP011102_IND_SouthEasternAsia_20
C6_AP011103_IND_SouthEasternAsia_20
C4_AB048705_AUS_Australia_26
C4_AB048704_AUS_Australia_26
B5_DQ463789_CAN_NorthernAmerica_39
B5_AB287318_USA_NorthernAmerica_13
B5_AB287316_USA_NorthernAmerica_13
B5_DQ463792_CAN_NorthernAmerica_39
B5_AB287321_GRL_NorthernAmerica_20
B5_AB287320_GRL_NorthernAmerica_20
B3_AB033555_IDN_SouthEasternAsia_19
B3_AB219430_PHL_SoutheasternAsia_13
B3_AP011089_IDN_SoutheasternAsia_20
B3_AB219429_PHL_SoutheasternAsia_13
B_AB241117_PHL_SoutheasternAsia_13
B1_AB073858_JPN_EasternAsia_17
B1_KP341007_JPN_EasternAsia_5
IA_DA45_MNG_EasternAsia_2121
B4_AB073835_VNM_SouthEasternAsia_17
B_DQ993686_VNM_SoutheasternAsia_12
F2a_AY311369_VEN_SouthAmerica_22
F2a_AY090455_NIC_CentralAmerica_21
F2b_DQ899146_VEN_SouthAmerica_12
F2b_DQ899144_VEN_SouthAmerica_12
F_AB166850_BOL_SouthAmerica_13
F4_AF223962_ARG_SouthAmerica_18
F3_X75663_NA_SouthSAmerica_26
F3_AB116549_PAN_CentralAmerica_15
F1b_FJ657525_ARG_SouthAmerica_15
F1b_AB116654_JPN_EasternAsia_15
F1a_AY090458_CRI_CentralAmerica_21
H_AY090454_NIC_CentralAmerica_21
H_AY090457_NIC_CentralAmerica_21
H_AB375163_MEX_CentralAmerica_10
H_AB059660_USA_NorthernAmerica_17
Gibbon_FM209516_TWN_EasternAsia_12
J_AB486012_JPN_EasternAsia_12
Orang_AF193863_19
Orang_EU155824_THA_SoutheasternAsia_11
Gibbon_U46935_28
Gibbon_AJ131571_GER_WesternEurope_19
Gibbon_AY781180_KHM_SoutheasternAsia_14
Chimp_AB032433_LBR_WesternAfrica_36
Chimp_AF222323_18
Gorilla_AJ131567_CMR_MiddleAfrica_19
Chimp_AY330911_CMR_MiddleAfrica_15
BA_RISE254_HUN_EasternEurope_4010
Sorsum_GER_WesternEurope_5240
BA_RISE563_GER_WesternEurope_4489
Karsdorf_GER_WesternEurope_7027
BA_RISE154_POL_EasternEurope_3852
D_X80925_GRC_SouthernEurope_23
D_AY796031_TUR_WesternAsia_14
D2GQ477455_POL_EasternEurope_12
D2_JN642163_LBN_WesternAsia_8
D2GQ477453_POL_EasternEurope_12
D_AB210818_JPN_EasternAsia_13
D1_FJ899792_CHN_EasternAsia_11
D1_KP322600_NA_WesternAsia_5
D1_JN642140_LBN_WesternAsia_8
D2_JN642160_LBN_WesternAsia_8
D3_JN688710_ARG_SouthAmerica_8
D3_JN688711_ARG_SouthAmerica_7
MG585269_ITA_SouthernEurope_450
D3_KC875319_IND_SouthernAsia_8
D_AY902768_USA_NorthernAmerica_13
D3_KP322602_IND_SouthernAsia_5
IA_DA29_KAZ_CentralAsia_823
D_AB188243_JPN_EasternAsia_14
IA_DA222_KAZ_CentralAsia_1168
D_GQ205377_IND_SouthernAsia_11
D_GQ205378_IND_SouthernAsia_11
D5_GQ205385_IND_SouthernAsia_10
D_GQ205389_IND_SouthernAsia_10
D5_KP322603_IND_SouthernAsia_5
D_GQ205382_IND_SouthernAsia_10
D_GQ205384_IND_SouthernAsia_10
D_DQ315779_IND_SouthernAsia_13
D_AB033558_JPN_EasternAsia_30
IA_DA27_KAZ_CentralAsia_1611
D_AB048701_AUS_Australia_26
D_AB048702_AUS_Australia_26
D_AB033559_PNG_Melanesia_30
D4_GQ922005_CAN_NorthernAmerica_34
D4_HE974378_MTQ_Caribbean_15
D4_KJ470896_BRA_SouthAmerica_9
D4_KJ470893_BRA_SouthAmerica_9
D4_KJ470898_BRA_SouthAmerica_8
Petersberg_GER_WesternEurope_951
D7_FJ904430_TUN_NorthernAfrica_13
D7_FJ904436_TUN_NorthernAfrica_11
D_AM494716_CAF_MiddleAfrica_11
IA_DA51_KGZ_CentralAsia_2298
E_X75657_27
E_X75664_27
G_AF405706_GER_WesternEurope_17
G_AB064312_USA_NorthernAmerica_17
G_AB056513_USA_NorthernAmerica_17
AF046996.1_Woolly_monkey_outgroup
A
C
B
F
H
Chimpanzee, Gibbon, Orang Utan
Gorilla
D
E
G
70.0

C

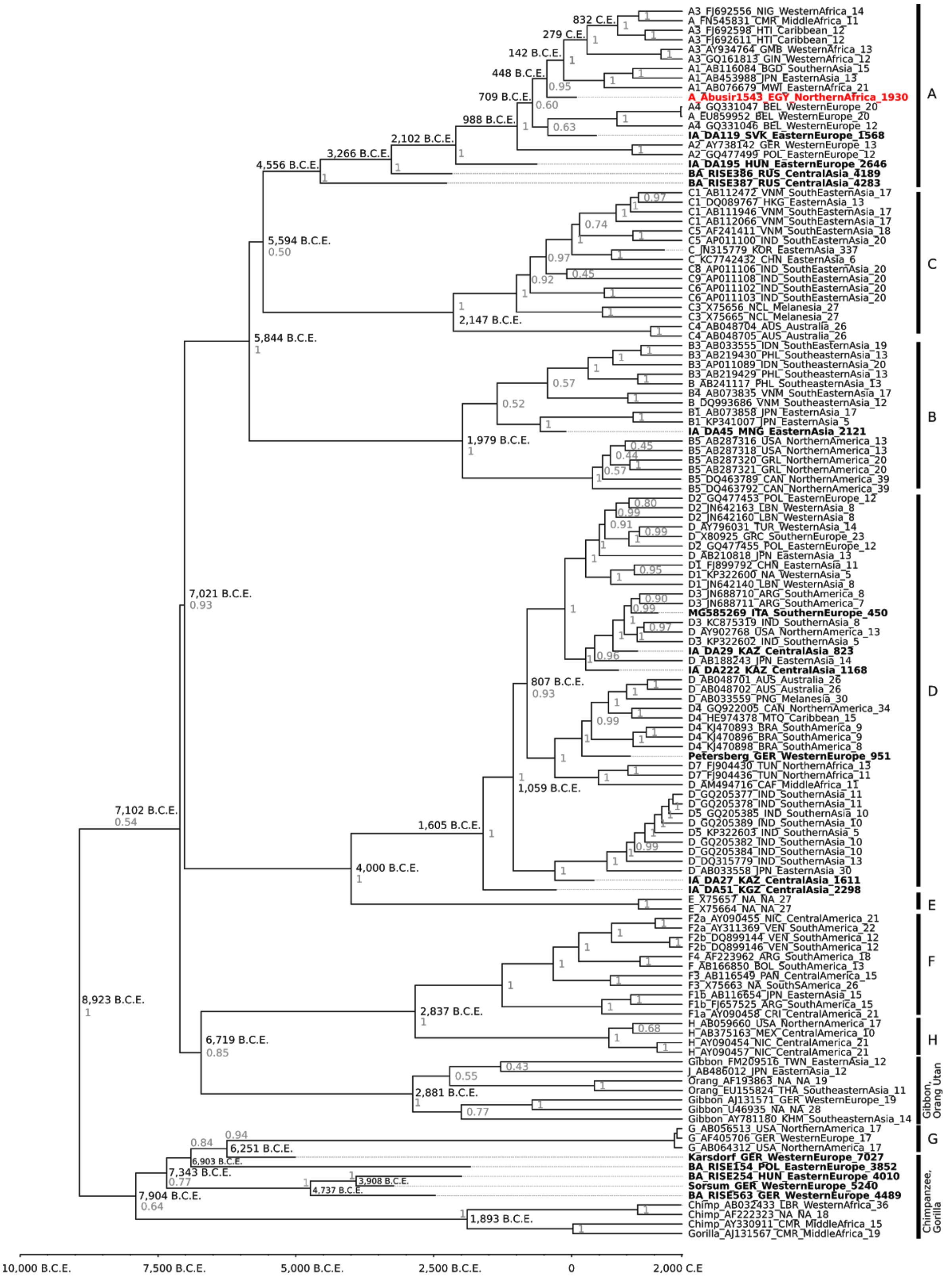

A3_FJ692556_NIG_WesternAfrica_14
A_FN545831_CMR_MiddleAfrica_11
A3_FJ692598_HTI_Caribbean_12
A3_FJ692611_HTI_Caribbean_12
A3_AY934764_GMB_WesternAfrica_13
A3_GQ161813_GIN_WesternAfrica_12
A1_AB116084_BGD_SouthernAsia_15
A1_AB453988_JPN_EasternAsia_13
A1_AB076679_MWI_EasternAfrica_21
A_Abusir1543_EGY_NorthernAfrica_1930
A4_GQ331047_BEL_WesternEurope_20
A_EU859952_BEL_WesternEurope_20
A4_GQ331046_BEL_WesternEurope_12
IA_DA119_SVK_EasternEurope_1568
A2_AY738142_GER_WesternEurope_13
A2_GQ477499_POL_EasternEurope_12
IA_DA195_HUN_EasternEurope_2646
BA_RISE386_RUS_CentralAsia_4189
BA_RISE387_RUS_CentralAsia_4283
C1_AB112472_VNM_SouthEasternAsia_17
C1_DQ089767_HKG_EasternAsia_13
C1_AB111946_VNM_SouthEasternAsia_17
C1_AB112066_VNM_SouthEasternAsia_17
C5_AF241411_VNM_SouthEasternAsia_18
C5_AP011100_IND_SouthEasternAsia_20
C_JN315779_KOR_EasternAsia_337
C_KC7742432_CHN_EasternAsia_6
C8_AP011106_IND_SouthEasternAsia_20
C9_AP011108_IND_SouthEasternAsia_20
C6_AP011102_IND_SouthEasternAsia_20
C6_AP011103_IND_SouthEasternAsia_20
C3_X75656_NCL_Melanesia_27
C3_X75665_NCL_Melanesia_27
C4_AB048704_AUS_Australia_26
C4_AB048705_AUS_Australia_26
B3_AB033555_IDN_SouthEasternAsia_19
B3_AB219430_PHL_SoutheasternAsia_13
B3_AP011089_IDN_SoutheasternAsia_20
B3_AB219429_PHL_SoutheasternAsia_13
B_AB241117_PHL_SoutheasternAsia_13
B4_AB073835_VNM_SouthEasternAsia_17
B_DQ993686_VNM_SoutheasternAsia_12
B1_AB073858_JPN_EasternAsia_17
B1_KP341007_JPN_EasternAsia_5
IA_DA45_MNG_EasternAsia_2121
B5_AB287316_USA_NorthernAmerica_13
B5_AB287318_USA_NorthernAmerica_13
B5_AB287320_GRL_NorthernAmerica_20
B5_AB287321_GRL_NorthernAmerica_20
B5_DQ463789_CAN_NorthernAmerica_39
B5_DQ463792_CAN_NorthernAmerica_39
D2_GQ477453_POL_EasternEurope_12
D2_JN642163_LBN_WesternAsia_8
D2_JN642160_LBN_WesternAsia_8
D_AY796031_TUR_WesternAsia_14
D_X80925_GRC_SouthernEurope_23
D2_GQ477455_POL_EasternEurope_12
D_AB210818_JPN_EasternAsia_13
D1_FJ899792_CHN_EasternAsia_11
D1_KP322600_NA_WesternAsia_5
D1_JN642140_LBN_WesternAsia_8
D3_JN688710_ARG_SouthAmerica_8
D3_JN688711_ARG_SouthAmerica_7
MG585269_ITA_SouthernEurope_450
D3_KC875319_IND_SouthernAsia_8
D_AY902768_USA_NorthernAmerica_13
D3_KP322602_IND_SouthernAsia_5
IA_DA29_KAZ_CentralAsia_823
D_AB188243_JPN_EasternAsia_14
IA_DA222_KAZ_CentralAsia_1168
D_AB048701_AUS_Australia_26
D_AB048702_AUS_Australia_26
D_AB033559_PNG_Melanesia_30
D4_GQ922005_CAN_NorthernAmerica_34
D4_HE974378_MTQ_Caribbean_15
D4_KJ470893_BRA_SouthAmerica_9
D4_KJ470896_BRA_SouthAmerica_9
D4_KJ470898_BRA_SouthAmerica_8
Petersberg_GER_WesternEurope_951
D7_FJ904430_TUN_NorthernAfrica_13
D7_FJ904436_TUN_NorthernAfrica_11
D_AM494716_CAF_MiddleAfrica_11
D_GQ205377_IND_SouthernAsia_11
D_GQ205378_IND_SouthernAsia_11
D5_GQ205385_IND_SouthernAsia_10
D_GQ205389_IND_SouthernAsia_10
D5_KP322603_IND_SouthernAsia_5
D_GQ205382_IND_SouthernAsia_10
D_GQ205384_IND_SouthernAsia_10
D_DQ315779_IND_SouthernAsia_13
D_AB033558_JPN_EasternAsia_30
IA_DA27_KAZ_CentralAsia_1611
IA_DA51_KGZ_CentralAsia_2298
E_X75657_NA_NA_27
E_X75664_NA_NA_27
F2a_AY090455_NIC_CentralAmerica_21
F2a_AY311369_VEN_SouthAmerica_22
F2b_DQ899144_VEN_SouthAmerica_12
F2b_DQ899146_VEN_SouthAmerica_12
F4_AF223962_ARG_SouthAmerica_18
F_AB166850_BOL_SouthAmerica_13
F3_AB116549_PAN_CentralAmerica_15
F3_X75663_NA_SouthSAmerica_26
F1b_AB116654_JPN_EasternAsia_15
F1b_FJ657525_ARG_SouthAmerica_15
F1a_AY090458_CRI_CentralAmerica_21
H_AB059660_USA_NorthernAmerica_17
H_AB375163_MEX_CentralAmerica_10
H_AY090454_NIC_CentralAmerica_21
H_AY090457_NIC_CentralAmerica_21
Gibbon_FM209516_TWN_EasternAsia_12
J_AB486012_JPN_EasternAsia_12
Orang_AF193863_NA_NA_19
Orang_EU155824_THA_SoutheasternAsia_11
Gibbon_AJ131571_GER_WesternEurope_19
Gibbon_U46935_NA_NA_28
Gibbon_AY781180_KHM_SoutheasternAsia_14
G_AB056513_USA_NorthernAmerica_17
G_AF405706_GER_WesternEurope_17
G_AB064312_USA_NorthernAmerica_17
Karsdorf_GER_WesternEurope_7027
BA_RISE154_POL_EasternEurope_3852
BA_RISE254_HUN_EasternEurope_4010
Sorsum_GER_WesternEurope_5240
BA_RISE563_GER_WesternEurope_4489
Chimp_AB032433_LBR_WesternAfrica_36
Chimp_AF222323_NA_NA_18
Chimp_AY330911_CMR_MiddleAfrica_15
Gorilla_AJ131567_CMR_MiddleAfrica_19
A
C
B
D
E
F
H
Gibbon, Orang Utan
G
Chimpanzee, Gorilla
10,000 B.C.E.
7,500 B.C.E.
5,000 B.C.E.
2,500 B.C.E.
0
2,000 C.E

Figure S9: Phylogenetic trees of Hepatitis B virus genomes. (A) Maximum likelihood tree based on 129 HBV genomes (supplementary Table S7). The ancient genomes are bolded, the newly sequenced genome in red and bolded. The bootstrap values are given for the main branches as node labels. (B) Maximum parsimony tree based on 129 HBV genomes (supplementary Table S7). The ancient genomes are bolded, the newly sequenced genome in red and bolded. The bootstrap values are given as node labels. (C) Dated Bayesian Maximum Clade Credibility tree reconstructed using a whole genome alignment of modern and ancient HBV strains (Table S7). Ancient samples are bold, the newly added genome Abusir1543 in red. The node labels are the median divergence times in years B.C.E. and C.E and the posterior values (grey).

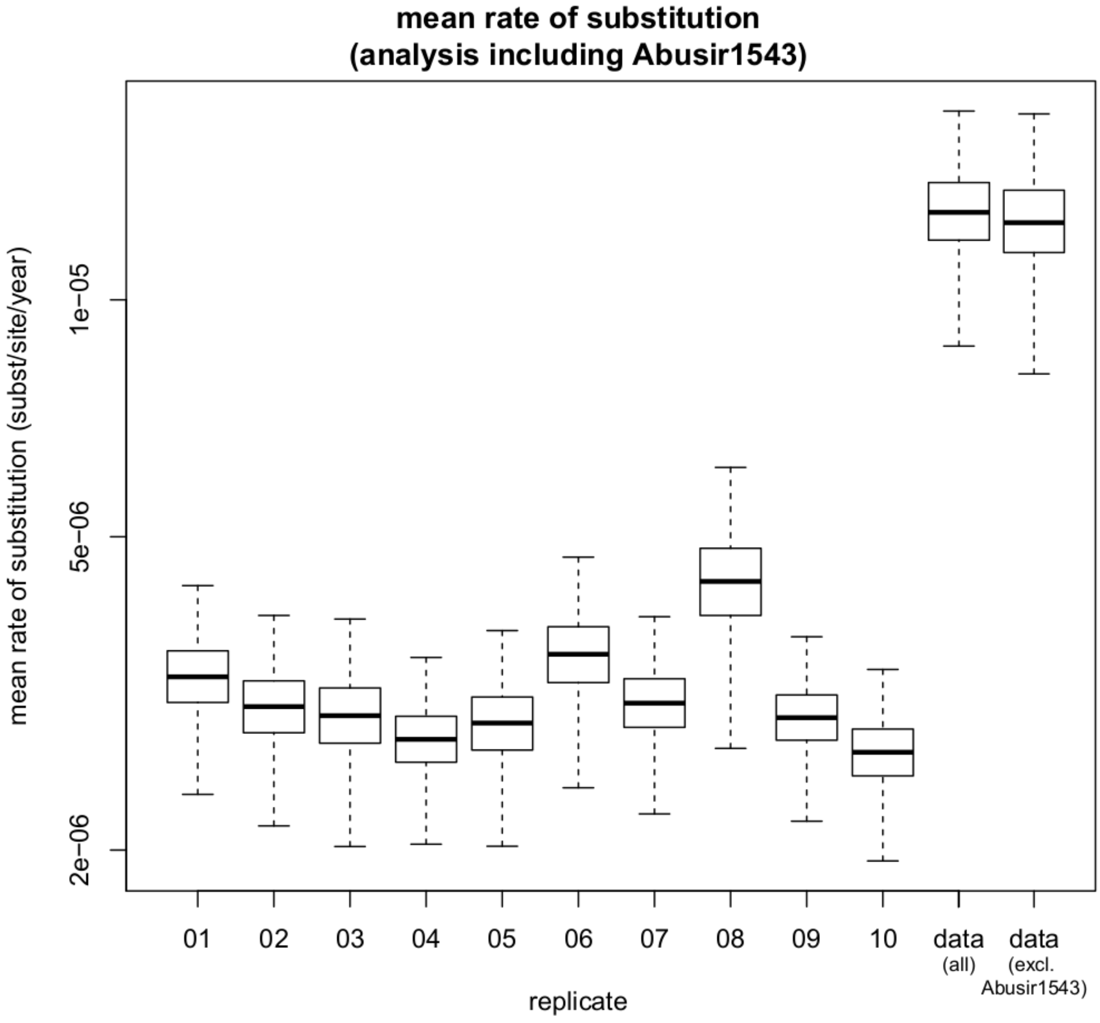

Figure S10: Date Randomization test for the HBV data set. BEAST analysis was performed for the original data set and ten replicates with randomly reassigned tip calibrations (ages of the samples). The lack of overlap between the timescale parameter estimates (here, the mean rate of nucleotide substitution) indicates a sufficient temporal signal for the molecular clock calibration and time-aware phylogenetic inference. Additionally an analysis excluding strain Abusir1543 was performed to examine its effect on the substitution rate estimate.

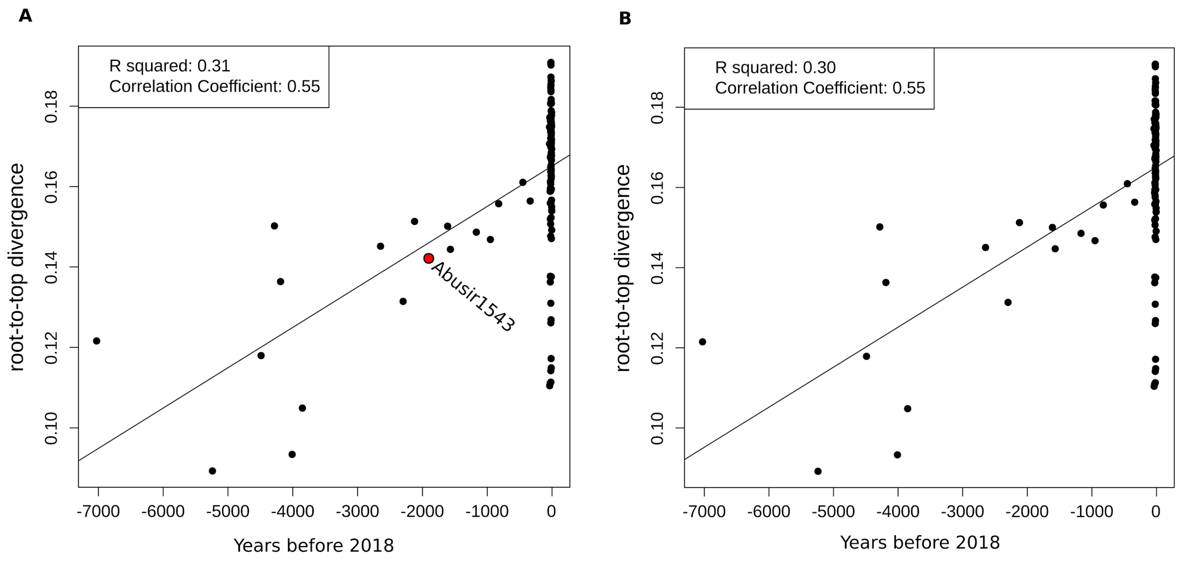

Figure S11: TempEst analysis for the HBV dataset. Analysis (A) with and (B) without the new strain Abusir1543 (red dot). The plot visualizes the phylogenetic root-to-tip distance relative to sampling time in years before present with the year 2018 as present. Both analyses show an almost identical temporal signal.

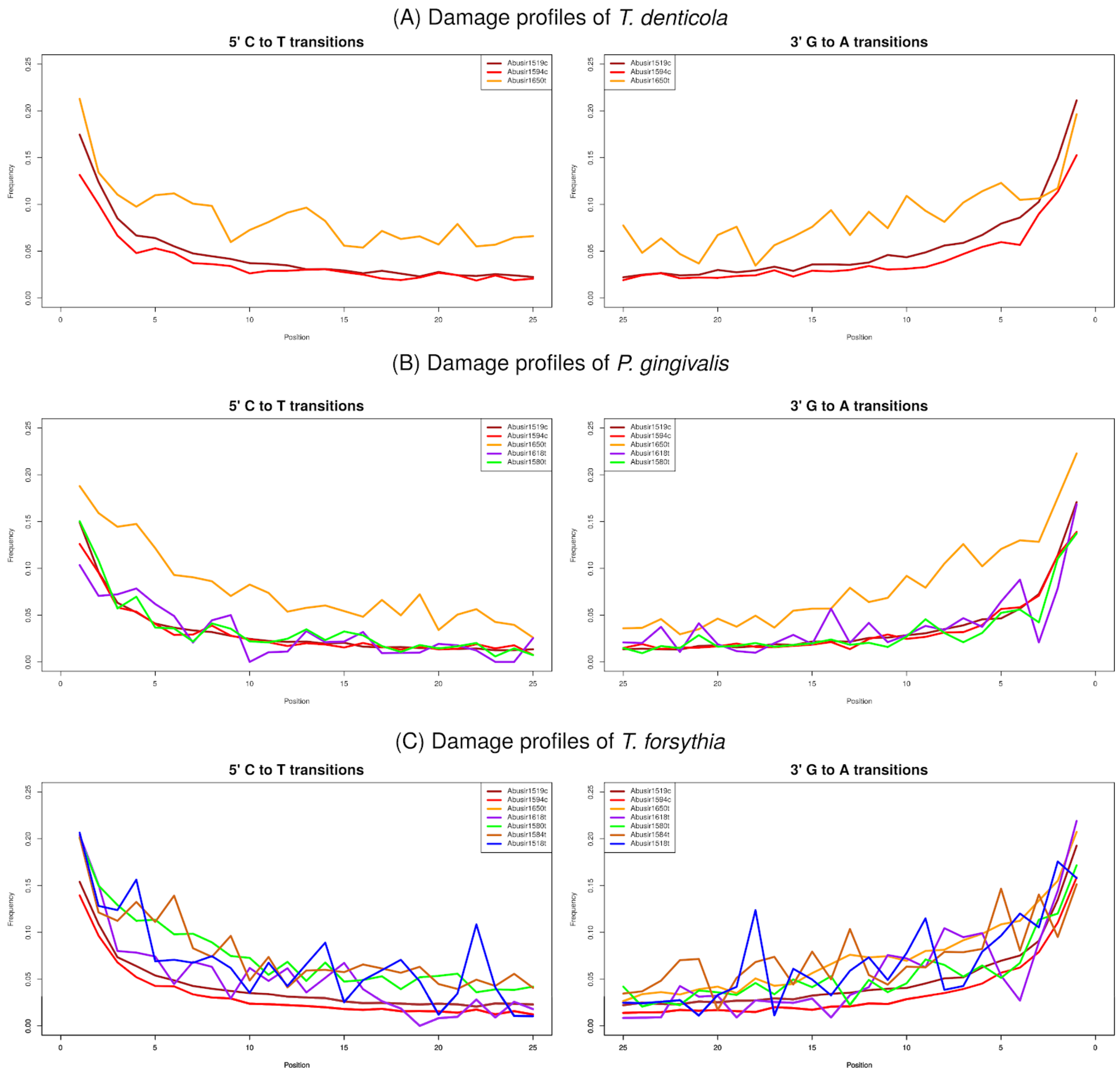

Figure S12: Combined damage profiles of Red Complex bacteria. Damage profiles of Red Complex bacteria (A) *Treponema denticola*, (B) *Porphyromonas gingivalis*, and (C) *Tannerella forsythia*.

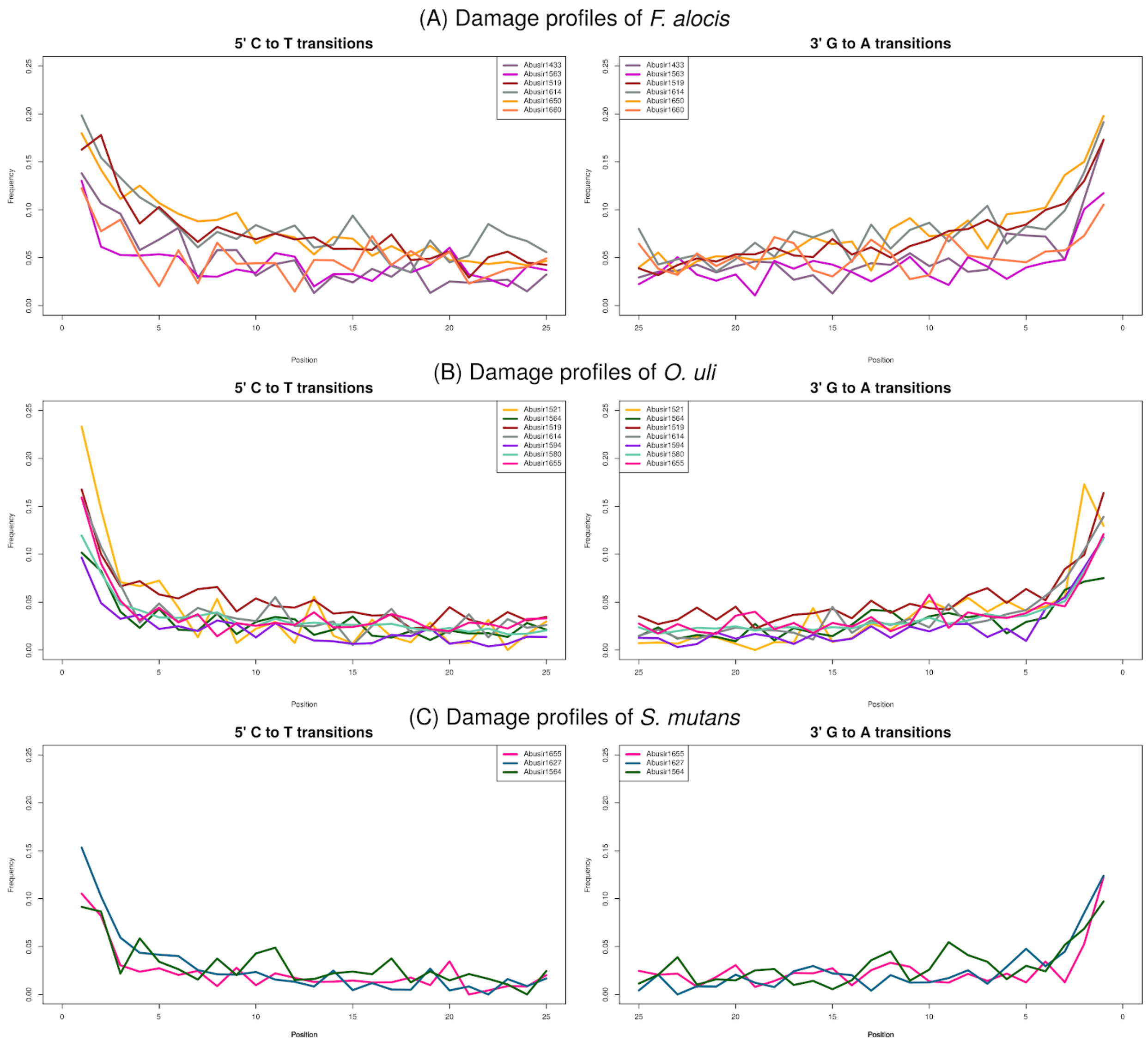

Figure S13: Combined damage profiles of oral pathogens. Damage profiles of reads mapping to (A) *Filifactor alocis,* (B) *Olsenella uli*, and (C) *Streptococcus mutans*.

Tables

Table S1: Overview of the number of samples and sampled tissue. Overview of all 133 samples taken from bone, soft tissue, and teeth. Number of samples are given per period and tissue.

Time period	Number of Samples used in analysis				Sum per time period
	Teeth	Bone	Soft tissue (skin spanning the vertebra)	Calculus	
First Intermediate Period	0	0	0	1	**1**
Pre-Ptolemaic Period	29	21	3	0	**53**
Ptolemaic Period	29	16	2	2	**49**
Roman Period	10	17	1	2	**30**
**Sum per tissue**	**68**	**54**	**6**	**5**	

Table S2: Sample overview. Sample overview with radiocarbon dates, sampled tissues, and accession IDs of all individuals used in this study. Individuals sampled multiple times are marked grey.

Individual	Dating	Time Period	Sampled Tissue	Citation for dating	Collection
Abusir1433	cal BC 793-604	Pre-Ptolemaic Period	Tooth	This study	Collection at University of Tübingen
Abusir1502	cal AD 235-336	Roman Period	Bone	This study	Collection at University of Tübingen
Abusir1504	cal AD 340-395	Roman Period	Bone, Calculus	Schuenemann et al.	Collection at University of Tübingen
Abusir1505	cal AD 264-391	Roman Period	Bone	This study	Collection at University of Tübingen
Abusir1506	cal AD 357-419	Roman Period	Bone	This study	Collection at University of Tübingen
Abusir1511	cal AD 138-200	Roman Period	Calculus	This study	Collection at University of Tübingen
Abusir1513	cal BC 398-373	Pre-Ptolemaic Period	Tooth	Schuenemann et al.	Collection at University of Tübingen
Abusir1515	cal BC 1304-1136	Pre-Ptolemaic Period	Tooth	Schuenemann et al.	Collection at University of Tübingen
Abusir1518	cal BC 979-914	Pre-Ptolemaic Period	Tooth	Schuenemann et al.	Collection at University of Tübingen
Abusir1519	cal BC 2196-2045	First Intermediate Period	Calculus	This study	Collection at University of Tübingen
Abusir1521	cal BC 797-674	Pre-Ptolemaic Period	Tooth	Schuenemann et al.	Collection at University of Tübingen
Abusir1526	cal BC 804-786	Pre-Ptolemaic Period	Tooth	Schuenemann et al.	Collection at University of Tübingen
Abusir1529	cal BC 369-211	Ptolemaic Period	Tooth	This study	Collection at University of Tübingen
Abusir1530	cal BC 770-567	Pre-Ptolemaic Period	Bone	Schuenemann et al.	Collection at University of Tübingen
Abusir1532	cal BC 804-792	Pre-Ptolemaic Period	Bone	Schuenemann et al.	Collection at University of Tübingen
Abusir1533	cal BC 358-208	Ptolemaic Period	Bone	Schuenemann et al.	Collection at University of Tübingen
Abusir1534	cal BC 902-842	Pre-Ptolemaic Period	Tooth	Schuenemann et al.	Collection at University of Tübingen
Abusir1536	cal AD 81-132	Roman Period	Bone	Schuenemann et al.	Collection at University of Tübingen
Abusir1537	cal BC 52-cal AD 16	Roman Period	Tooth	This study	Collection at University of Tübingen
Abusir1538	cal BC 87-cal AD 2	Ptolemaic Period	Tooth	Schuenemann et al.	Collection at University of Tübingen
Abusir1539	cal BC 899-841	Pre-Ptolemaic Period	Bone	Schuenemann et al.	Collection at University of Tübingen
Abusir1541	cal BC 185-107	Ptolemaic Period	Bone	Schuenemann et al.	Collection at University of Tübingen
Abusir1543	cal AD 54- 124	Roman Period	Bone, Soft Tissue	Lalremruata et al.	Collection at University of Tübingen
Abusir1545	cal BC 349-175	Ptolemaic Period	Tooth	This study	Collection at University of Tübingen
Abusir1546	cal BC 362-210	Ptolemaic Period	Tooth	Schuenemann et al.	Collection at University of Tübingen
Abusir1547	cal AD 91-212	Roman Period	Tooth	Schuenemann et al.	Collection at University of Tübingen
Abusir1549	cal BC 45-cal AD 4	Roman Period	Bone	Schuenemann et al.	Collection at University of Tübingen
Abusir1550	cal BC 367-212	Ptolemaic Period	Tooth	Schuenemann et al.	Collection at University of Tübingen
Abusir1553	cal BC 794-671	Pre-Ptolemaic Period	Bone	Schuenemann et al.	Collection at University of Tübingen
Abusir1554	cal BC 402- 385	Pre-Ptolemaic Period	Bone, Soft Tissue	Lalremruata et al.	Collection at University of Tübingen
Abusir1555	cal AD 25-111	Roman Period	Bone	Schuenemann et al.	Collection at University of Tübingen
Abusir1556	cal BC 344-169	Ptolemaic Period	Bone	Schuenemann et al.	Collection at University of Tübingen
Abusir1557	cal BC 196-113	Ptolemaic Period	Bone	This study	Collection at University of Tübingen

Abusir1558	cal BC 200-110	Ptolemaic Period	Bone	This study	Collection at University of Tübingen
Abusir1559	cal BC 19-<br>cal AD 55	Roman Period	Bone	This study	Collection at University of Tübingen
Abusir1560	cal BC 108-3	Ptolemaic Period	Tooth	This study	Collection at University of Tübingen
Abusir1561	cal BC 352-189	Ptolemaic Period	Tooth	This study	Collection at University of Tübingen
Abusir1563	cal BC 158-54	Ptolemaic Period	Tooth	Schuenemann et al.	Collection at University of Tübingen
Abusir1564	cal BC 358- 204	Ptolemaic Period	Bone,<br>Tooth,<br>Soft Tissue	Lalremruata et al.	Collection at University of Tübingen
Abusir1565	cal BC 151-48	Ptolemaic Period	Bone	Schuenemann et al.	Collection at University of Tübingen
Abusir1566	cal BC 749-517	Pre-Ptolemaic Period	Bone	Schuenemann et al.	Collection at University of Tübingen
Abusir1568	cal BC 1388-1311	Pre-Ptolemaic Period	Tooth	Schuenemann et al.	Collection at University of Tübingen
Abusir1569	cal BC 795-674	Pre-Ptolemaic Period	Tooth	Schuenemann et al.	Collection at University of Tübingen
Abusir1570	cal BC 151-46	Ptolemaic Period	Tooth	Schuenemann et al.	Collection at University of Tübingen
Abusir1572	cal AD 83-208	Roman Period	Bone	Schuenemann et al.	Collection at University of Tübingen
Abusir1573	cal BC 97-2	Ptolemaic Period	Bone,Tooth	Schuenemann et al.	Collection at University of Tübingen
Abusir1574	cal BC 109-40	Ptolemaic Period	Calculus	This study	Collection at University of Tübingen
Abusir1576	cal BC 352-200	Ptolemaic Period	Bone	Schuenemann et al.	Collection at University of Tübingen
Abusir1577	cal AD 26-84	Roman Period	Bone	Schuenemann et al.	Collection at University of Tübingen
Abusir1579	cal BC 356-204	Ptolemaic Period	Tooth	This study	Collection at University of Tübingen
Abusir1580	cal BC 394-239	Pre-Ptolemaic Period	Tooth	Schuenemann et al.	Collection at University of Tübingen
Abusir1582	cal AD 35-120	Roman Period	Bone	Schuenemann et al.	Collection at University of Tübingen
Abusir1583	cal BC 358-208	Ptolemaic Period	Tooth	This study	Collection at University of Tübingen
Abusir1584	cal BC 799-781	Pre-Ptolemaic Period	Tooth	Schuenemann et al.	Collection at University of Tübingen
Abusir1585	cal BC 382- 234	Ptolemaic Period	Bone, Soft<br>Tissue	Lalremruata et al.	Collection at University of Tübingen
Abusir1587	cal BC 97-1	Ptolemaic Period	Tooth	This study	Collection at University of Tübingen
Abusir1588	cal BC 344-126	Ptolemaic Period	Bone	Schuenemann et al.	Collection at University of Tübingen
Abusir1590	cal BC 395-263	Pre-Ptolemaic Period	Tooth	Schuenemann et al.	Collection at University of Tübingen
Abusir1591	cal BC 797-771	Pre-Ptolemaic Period	Tooth	Schuenemann et al.	Collection at University of Tübingen
Abusir1594	cal BC 369-211	Ptolemaic Period	Tooth,<br>Calculus	Schuenemann et al.	Collection at University of Tübingen
Abusir1595	cal BC 975-905	Pre-Ptolemaic Period	Tooth	Schuenemann et al.	Collection at University of Tübingen
Abusir1596	cal BC 792-603	Pre-Ptolemaic Period	Tooth	Schuenemann et al.	Collection at University of Tübingen
Abusir1599	cal BC 152-44	Ptolemaic Period	Tooth	This study	Collection at University of Tübingen
Abusir1601	cal AD 5-54	Roman Period	Tooth	Schuenemann et al.	Collection at University of Tübingen
Abusir1602	cal BC 357-204	Ptolemaic Period	Tooth	Schuenemann et al.	Collection at University of Tübingen
Abusir1603	cal BC 399-385	Pre-Ptolemaic Period	Bone	This study	Collection at University of Tübingen
Abusir1604	cal BC 750-525	Pre-Ptolemaic Period	Bone	Schuenemann et al.	Collection at University of Tübingen
Abusir1605	cal BC 776-569	Pre-Ptolemaic Period	Bone	Schuenemann et al.	Collection at University of Tübingen
Abusir1606	cal BC 769-560	Pre-Ptolemaic Period	Bone	This study	Collection at University of Tübingen
Abusir1607	cal AD 26-81	Roman Period	Tooth	This study	Collection at University of Tübingen
Abusir1608	cal BC 801- 777	Pre-Ptolemaic Period	Bone, Soft<br>Tissue	Lalremruata et al.	Collection at University of Tübingen
Abusir1609	cal BC 769-560	Pre-Ptolemaic Period	Bone	Schuenemann et al.	Collection at University of Tübingen

Abusir1611	cal AD 84-129	Roman Period	Bone	Schuenemann et al.	Collection at University of Tübingen
Abusir1612	cal BC 758-552	Pre-Ptolemaic Period	Tooth	Schuenemann et al.	Collection at University of Tübingen
Abusir1614	cal BC 753-544	Pre-Ptolemaic Period	Bone,Tooth	Schuenemann et al.	Collection at University of Tübingen
Abusir1615	cal BC 895-834	Pre-Ptolemaic Period	Tooth	Schuenemann et al.	Collection at University of Tübingen
Abusir1616	cal BC 992-923	Pre-Ptolemaic Period	Tooth	Schuenemann et al.	Collection at University of Tübingen
Abusir1617	cal BC 1111-998	Pre-Ptolemaic Period	Tooth	Schuenemann et al.	Collection at University of Tübingen
Abusir1618	cal BC 510-408	Pre-Ptolemaic Period	Tooth	Schuenemann et al.	Collection at University of Tübingen
Abusir1619	cal BC 790-671	Pre-Ptolemaic Period	Bone	Schuenemann et al.	Collection at University of Tübingen
Abusir1620	cal BC 337-114	Ptolemaic Period	Tooth	This study	Collection at University of Tübingen
Abusir1621	cal BC 167-61	Ptolemaic Period	Tooth	This study	Collection at University of Tübingen
Abusir1622	cal BC 806- 784	Pre-Ptolemaic Period	Bone, Soft Tissue	Lalremruata et al.	Collection at University of Tübingen
Abusir1623	cal BC 353-201	Ptolemaic Period	Tooth	This study	Collection at University of Tübingen
Abusir1624	cal BC 44- cal AD 16	Roman Period	Bone	Schuenemann et al.	Collection at University of Tübingen
Abusir1627	cal BC 92-1	Ptolemaic Period	Bone, Tooth	Schuenemann et al.	Collection at University of Tübingen
Abusir1630	cal BC 342-117	Ptolemaic Period	Bone	Schuenemann et al.	Collection at University of Tübingen
Abusir1631	cal AD 32-122	Roman Period	Tooth	Schuenemann et al.	Collection at University of Tübingen
Abusir1633	cal BC 399-376	Pre-Ptolemaic Period	Tooth	Schuenemann et al.	Collection at University of Tübingen
Abusir1634	cal BC 508-406	Pre-Ptolemaic Period	Bone	Schuenemann et al.	Collection at University of Tübingen
Abusir1635	cal BC 352-195	Ptolemaic Period	Tooth	Schuenemann et al.	Collection at University of Tübingen
Abusir1636	cal BC 389-235	Pre-Ptolemaic Period	Tooth	Schuenemann et al.	Collection at University of Tübingen
Abusir1642	cal BC 391-260	Pre-Ptolemaic Period	Bone	Schuenemann et al.	Collection at University of Tübingen
Abusir1645	cal BC 405-394	Pre-Ptolemaic Period	Bone	Schuenemann et al.	Collection at University of Tübingen
Abusir1647	cal BC 399-386	Pre-Ptolemaic Period	Tooth	This study	Collection at University of Tübingen
Abusir1648	cal BC 87-cal AD 1	Ptolemaic Period	Bone,Tooth	Schuenemann et al.	Collection at University of Tübingen
Abusir1649	cal BC 823-785	Pre-Ptolemaic Period	Bone	Schuenemann et al.	Collection at University of Tübingen
Abusir1650	cal BC 889-803	Pre-Ptolemaic Period	Tooth	Schuenemann et al.	Collection at University of Tübingen
Abusir1651	cal BC 347-168	Ptolemaic Period	Tooth	Schuenemann et al.	Collection at University of Tübingen
Abusir1652	cal AD 27-83	Roman Period	Bone	Schuenemann et al.	Collection at University of Tübingen
Abusir1653	cal BC 788-595	Pre-Ptolemaic Period	Bone	Schuenemann et al.	Collection at University of Tübingen
Abusir1654	cal BC 384-235	Pre-Ptolemaic Period	Tooth	Schuenemann et al.	Collection at University of Tübingen
Abusir1655	cal BC 1211-1126	Pre-Ptolemaic Period	Tooth	Schuenemann et al.	Collection at University of Tübingen
Abusir1656	cal BC 164-60	Ptolemaic Period	Bone	Schuenemann et al.	Collection at University of Tübingen
Abusir1658	cal AD 5-63	Roman Period	Bone	This study	Collection at University of Tübingen
Abusir1660	cal BC 348-170	Ptolemaic Period	Tooth	This study	Collection at University of Tübingen
Abusir1665	cal BC 44-cal AD 2	Roman Period	Tooth	This study	Collection at University of Tübingen
Abusir1666	cal BC 37- cal AD 48	Roman Period	Bone	Schuenemann et al.	Collection at University of Tübingen
Abusir1667	cal BC 790-603	Pre-Ptolemaic Period	Bone	Schuenemann et al.	Collection at University of Tübingen
Abusir1668	cal BC 357-206	Ptolemaic Period	Tooth,Bone	Schuenemann et al.	Collection at University of Tübingen
Abusir1671	cal BC 156-53	Ptolemaic Period	Tooth	Schuenemann et al.	Collection at University of Tübingen
Abusir1672	cal BC 43-	Roman Period	Tooth	Schuenemann et al.	Collection at University of Tübingen

	cal AD 45				
Abusir1673	cal BC 756-545	Pre-Ptolemaic Period	Tooth	Schuenemann et al.	Collection at University of Tübingen
Abusir3533	cal BC 759-551	Pre-Ptolemaic Period	Tooth	Schuenemann et al.	Felix von Luschan Skull Collection
Abusir3536	cal BC 43-<br>cal AD 15	Roman Period	Tooth	Schuenemann et al.	Felix von Luschan Skull Collection
Abusir3544	cal AD 386-426	Roman Period	Tooth	Schuenemann et al.	Felix von Luschan Skull Collection
Abusir3552	cal AD 261-382	Roman Period	Tooth	Schuenemann et al.	Felix von Luschan Skull Collection
Abusir3578	cal BC 364-211	Ptolemaic Period	Tooth	Schuenemann et al.	Felix von Luschan Skull Collection
Abusir3610	cal BC 355-204	Ptolemaic Period	Tooth	Schuenemann et al.	Felix von Luschan Skull Collection

Table S3: Microbial composition of the samples.

Samples	Viruses	Archaea	Bacteria
Bone samples	8.61%	0.14%	91.24%
Tooth samples	3.71%	0.12%	96.16%
Soft tissue samples	0.65%	0.04%	99.31%
Calculus samples	2.43%	2.57%	95.00%

Table S4: See additional file 2.

Table S5: See additional file 3.

Table S6: Approach to determine the genotype of Abusir1630.

	SNP position (NC002677.1)		WT	Abusir1630	Results
	**Old numeration** [46]	**New Numeration** [40]			
SNP type	1642875	1642879	G	T	3
	2935685	2935693	A	C	
	14676	14676	C	C	
SNP sub-type	1295192	1295195	A	G	L
	2312059	2312066	C	G	
	413902	413903	G	A	
	20910	20910	G	G	
	14676	14676	C	C	

Table S7: HBV strains used for analysis.

Accession ID	Alternative Sample Name	Genotype	Host	Used for Network	Used for BEAST	Used for ML/MP	Used for recombination
FN545831		A	Human		x	x	
AB116092		A	Human	x			
AB194951		A	Human	x			
AF297621		A	Human	x			
AM184125		A	Human	x			
AY161138		A	Human	x			
AY233275		A	Human	x			
AY233280		A	Human	x			
AY233290		A	Human	x			
EU366129		A	Human	x			
EU859930		A	Human	x			
FJ692558		A	Human	x			
FJ692592		A	Human	x			
FJ692596		A	Human	x			
FJ692608		A	Human	x			
FM199978		A	Human	x			
FN545826		A	Human	x			
FN545828		A	Human	x			
FN545833		A	Human	x			
JN182323		A	Human	x			
JN182327		A	Human	x			
JQ023661		A	Human	x			
JQ707397		A	Human	x			
KF214660		A	Human	x			
KF922415		A	Human	x			
KF922430		A	Human	x			
KF922434		A	Human	x			
KJ854685		A	Human	x			
KJ854693		A	Human	x			
KM606737		A	Human	x			
KP168428		A	Human	x			
KP168435		A	Human	x			
KT151612		A	Human	x			
KU605533		A	Human	x			
KU605537		A	Human	x			
KX357650		A	Human	x			
EU859952		A	Human	x	x	x	
ERS3636018, ERS3636025, ERS3636093 - ERS3636101	Abusir1543	A	Human	x	x	x	x
AB116084		A1	Human		x	x	

AB453988		A1	Human		x	x	
AB076679		A1	Human		x	x	x
AY738142		A2	Human		x	x	
GQ477499		A2	Human		x	x	x
AY934764		A3	Human		x	x	
FJ692556		A3	Human		x	x	
FJ692598		A3	Human		x	x	
FJ692611		A3	Human		x	x	
GQ161813		A3	Human		x	x	x
GQ331046		A4	Human		x	x	x
GQ331047		A6	Human		x	x	
DQ993686		B	Human		x	x	
KP341007		B	Human		x	x	
AB010289		B	Human	x			
AB014366		B	Human	x			
AB031267		B	Human	x			
AB073821		B	Human	x			
AB073836		B	Human	x			
AB073849		B	Human	x			
AB073853		B	Human	x			
AB100695		B	Human	x			
AB106884		B	Human	x			
AB212625		B	Human	x			
AB231909		B	Human	x			
AB246340		B	Human	x			
AB287315		B	Human	x			
AB287323		B	Human	x			
AB300371		B	Human	x			
AB555498		B	Human	x			
AB642093		B	Human	x			
AB642101		B	Human	x			
AB713528		B	Human	x			
AB828708		B	Human	x			
AB900098		B	Human	x			
AB900107		B	Human	x			
AB931169		B	Human	x			
AY206383		B	Human	x			
AY206391		B	Human	x			
AY800392		B	Human	x			
DQ463791		B	Human	x			
DQ993684		B	Human	x			
DQ995802		B	Human	x			
DQ995804		B	Human	x			

EF494381		B	Human	x			
EU158262		B	Human	x			
EU522072		B	Human	x			
EU939634		B	Human	x			
EU939677		B	Human	x			
EU939678		B	Human	x			
FJ023631		B	Human	x			
FJ032342		B	Human	x			
FJ386582		B	Human	x			
FJ386648		B	Human	x			
FJ562260		B	Human	x			
GQ358137		B	Human	x			
GQ358146		B	Human	x			
GQ358148		B	Human	x			
GQ358151		B	Human	x			
GQ924621		B	Human	x			
GQ924624		B	Human	x			
GQ924626		B	Human	x			
GQ924630		B	Human	x			
GQ924635		B	Human	x			
GQ924637		B	Human	x			
GQ924641		B	Human	x			
GQ924645		B	Human	x			
GQ924656		B	Human	x			
HM011466		B	Human	x			
HM011467		B	Human	x			
HM011471		B	Human	x			
HM011475		B	Human	x			
HM011476		B	Human	x			
HM011478		B	Human	x			
HM011482		B	Human	x			
HM011483		B	Human	x			
HM011487		B	Human	x			
HM011490		B	Human	x			
HM011496		B	Human	x			
HM011499		B	Human	x			
HM011503		B	Human	x			
HQ700546		B	Human	x			
JF436921		B	Human	x			
JN827419		B	Human	x			
JQ027311		B	Human	x			
JQ027312		B	Human	x			
JQ027313		B	Human	x			

JQ027330		B	Human	x			
JQ027334		B	Human	x			
JQ429081		B	Human	x			
JQ707737		B	Human	x			
JX026879		B	Human	x			
JX661471		B	Human	x			
KC774370		B	Human	x			
KJ173297		B	Human	x			
KJ173342		B	Human	x			
KJ173379		B	Human	x			
KJ173401		B	Human	x			
KJ410502		B	Human	x			
KJ790200		B	Human	x			
KJ803795		B	Human	x			
KJ803796		B	Human	x			
KJ803805		B	Human	x			
KJ803808		B	Human	x			
KJ803817		B	Human	x			
KJ803820		B	Human	x			
KM875420		B	Human	x			
KP148414		B	Human	x			
KP148452		B	Human	x			
KP148582		B	Human	x			
KP406278		B	Human	x			
KP659249		B	Human	x			
KP659250		B	Human	x			
KU964274		B	Human	x			
KU964383		B	Human	x			
KX276770		B	Human	x			
KX276772		B	Human	x			
KX276774		B	Human	x			
KX276783		B	Human	x			
KX276785		B	Human	x			
KX276786		B	Human	x			
KX276787		B	Human	x			
KX276791		B	Human	x			
KX276792		B	Human	x			
KX276794		B	Human	x			
KX276795		B	Human	x			
KX276796		B	Human	x			
KX276797		B	Human	x			
KX276798		B	Human	x			
KX276800		B	Human	x			

KX276806		B	Human	x			
KX276807		B	Human	x			
KX276812		B	Human	x			
KX276813		B	Human	x			
KX276815		B	Human	x			
KX276817		B	Human	x			
KX276819		B	Human	x			
KX276821		B	Human	x			
KX276825		B	Human	x			
KX276827		B	Human	x			
KX276830		B	Human	x			
KX276858		B	Human	x			
AB241117		B	Human	x	x	x	
AB073858		B1	Human		x	x	x
AB219430		B3	Human		x	x	
AP011089		B3	Human		x	x	
AB219429		B3	Human	x	x	x	x
AB033555		B3	Human		x	x	
AB073835		B4	Human		x	x	x
AB287316		B5	Human		x	x	
AB287318		B5	Human		x	x	
AB287320		B5	Human		x	x	
DQ463789		B5	Human		x	x	
DQ463792		B5	Human		x	x	
AB287321		B5	Human		x	x	x
KC774243		C	Human		x	x	
AB014371		C	Human	x			
AB049610		C	Human	x			
AB074047		C	Human	x			
AB111120		C	Human	x			
AB112063		C	Human	x			
AB113878		C	Human	x			
AB115417		C	Human	x			
AB176642		C	Human	x			
AB195930		C	Human	x			
AB195931		C	Human	x			
AB198079		C	Human	x			
AB300361		C	Human	x			
AB367392		C	Human	x			
AB367420		C	Human	x			
AB670258		C	Human	x			
AB670259		C	Human	x			
AB670263		C	Human	x			

AB670285		C	Human	x			
AB670295		C	Human	x			
AB670298		C	Human	x			
AB697502		C	Human	x			
AB697510		C	Human	x			
AB900109		C	Human	x			
AB900113		C	Human	x			
AB931170		C	Human	x			
AP011099		C	Human	x			
AY167091		C	Human	x			
AY206386		C	Human	x			
AY641559		C	Human	x			
AY641563		C	Human	x			
D23682		C	Human	x			
DQ089768		C	Human	x			
DQ089777		C	Human	x			
DQ089785		C	Human	x			
DQ089788		C	Human	x			
DQ089790		C	Human	x			
DQ089795		C	Human	x			
DQ089802		C	Human	x			
DQ089804		C	Human	x			
DQ890381		C	Human	x			
EU306725		C	Human	x			
EU498227		C	Human	x			
EU522071		C	Human	x			
EU670263		C	Human	x			
EU796069		C	Human	x			
EU939539		C	Human	x			
EU939568		C	Human	x			
EU939597		C	Human	x			
FJ386601		C	Human	x			
FJ386626		C	Human	x			
FJ386644		C	Human	x			
FJ787452		C	Human	x			
FJ899767		C	Human	x			
FJ899783		C	Human	x			
GQ358157		C	Human	x			
GQ358158		C	Human	x			
GQ377586		C	Human	x			
GQ377632		C	Human	x			
GQ475321		C	Human	x			
GQ924642		C	Human	x			

GQ924643		C	Human	x			
HM011479		C	Human	x			
HM011488		C	Human	x			
HM011495		C	Human	x			
HQ700456		C	Human	x			
HQ700506		C	Human	x			
HQ700516		C	Human	x			
HQ700517		C	Human	x			
HQ700522		C	Human	x			
HQ700564		C	Human	x			
HQ700575		C	Human	x			
HQ700576		C	Human	x			
JF828925		C	Human	x			
JN827415		C	Human	x			
JN827421		C	Human	x			
JQ027317		C	Human	x			
JQ027324		C	Human	x			
JQ040132		C	Human	x			
JQ040167		C	Human	x			
JQ429078		C	Human	x			
JQ801498		C	Human	x			
JX507211		C	Human	x			
JX870000		C	Human	x			
KC774180		C	Human	x			
KC774196		C	Human	x			
KC774214		C	Human	x			
KC774226		C	Human	x			
KC774238		C	Human	x			
KC774240		C	Human	x			
KC774244		C	Human	x			
KC774297		C	Human	x			
KC774302		C	Human	x			
KC774351		C	Human	x			
KC774352		C	Human	x			
KC774357		C	Human	x			
KF214670		C	Human	x			
KF214673		C	Human	x			
KF873514		C	Human	x			
KF873526		C	Human	x			
KF873536		C	Human	x			
KF873541		C	Human	x			
KF873544		C	Human	x			
KJ410494		C	Human	x			

KJ410496		C	Human	x			
KJ410515		C	Human	x			
KJ803766		C	Human	x			
KJ803777		C	Human	x			
KJ803779		C	Human	x			
KJ803790		C	Human	x			
KJ803809		C	Human	x			
KJ803818		C	Human	x			
KJ803823		C	Human	x			
KJ803826		C	Human	x			
KR013837		C	Human	x			
KR013859		C	Human	x			
KR013871		C	Human	x			
KT364751		C	Human	x			
KU679937		C	Human	x			
KU679947		C	Human	x			
KU679951		C	Human	x			
KU964045		C	Human	x			
KU964236		C	Human	x			
KU964358		C	Human	x			
KX276836		C	Human	x			
KX276841		C	Human	x			
KX276844		C	Human	x			
KX276846		C	Human	x			
KX276848		C	Human	x			
KX276850		C	Human	x			
KX276853		C	Human	x			
KX276855		C	Human	x			
AB112472		C	Human	x	x	x	x
AB111946		C1	Human		x	x	
AB112066		C1	Human		x	x	
DQ089767		C1	Human		x	x	
X75656		C3	Human		x	x	x
X75665		C3	Human	x	x	x	
AB048705		C4	Human		x	x	
AB048704		C4	Human		x	x	x
AP011100		C5	Human		x	x	
AF241411		C5	Human		x	x	x
AP011103		C6	Human		x	x	
AP011102		C6	Human		x	x	x
AP011106		C8	Human	x	x	x	x
AP011108		C9	Human		x	x	x
AB048702		D	Human		x	x	

AB188243		D	Human		x	x	
AB210818		D	Human		x	x	
AM494716		D	Human		x	x	
AY796031		D	Human		x	x	
AY902768		D	Human		x	x	
DQ315779		D	Human		x	x	
GQ205377		D	Human		x	x	
GQ205378		D	Human		x	x	
GQ205384		D	Human		x	x	
GQ205389		D	Human		x	x	
KC875319		D	Human		x	x	
KP322600		D	Human		x	x	
KP322602		D	Human		x	x	
KP322603		D	Human		x	x	
X80925		D	Human		x	x	
AB119255		D	Human	x			
AB188241		D	Human	x			
AB270541		D	Human	x			
AB330367		D	Human	x			
AB555496		D	Human	x			
AB555500		D	Human	x			
AB674416		D	Human	x			
AB674424		D	Human	x			
AB674425		D	Human	x			
AB674427		D	Human	x			
AB674428		D	Human	x			
AF043594		D	Human	x			
AJ344116		D	Human	x			
AJ627218		D	Human	x			
AJ627220		D	Human	x			
AJ627222		D	Human	x			
AY090452		D	Human	x			
DQ304548		D	Human	x			
DQ315778		D	Human	x			
DQ399006		D	Human	x			
DQ464173		D	Human	x			
DQ464174		D	Human	x			
DQ486025		D	Human	x			
EU155893		D	Human	x			
EU414139		D	Human	x			
EU414140		D	Human	x			
EU414141		D	Human	x			
EU594432		D	Human	x			

EU594434		D	Human	x			
FJ904395		D	Human	x			
FJ904402		D	Human	x			
FJ904422		D	Human	x			
FJ904426		D	Human	x			
FJ904427		D	Human	x			
FJ904433		D	Human	x			
FJ904438		D	Human	x			
FJ904439		D	Human	x			
FJ904445		D	Human	x			
GQ167302		D	Human	x			
GQ184322		D	Human	x			
GQ205380		D	Human	x			
GQ477452		D	Human	x			
GQ477456		D	Human	x			
GQ922002		D	Human	x			
GQ922003		D	Human	x			
GU456637		D	Human	x			
GU456643		D	Human	x			
GU456651		D	Human	x			
GU456654		D	Human	x			
GU456658		D	Human	x			
GU456665		D	Human	x			
GU456669		D	Human	x			
GU456674		D	Human	x			
GU456678		D	Human	x			
GU456679		D	Human	x			
GU456682		D	Human	x			
GU456684		D	Human	x			
HQ700449		D	Human	x			
HQ700458		D	Human	x			
HQ700510		D	Human	x			
HQ700513		D	Human	x			
JF754588		D	Human	x			
JF754592		D	Human	x			
JF754597		D	Human	x			
JF754611		D	Human	x			
JF754612		D	Human	x			
JF754617		D	Human	x			
JF754631		D	Human	x			
JN040762		D	Human	x			
JN040766		D	Human	x			
JN040768		D	Human	x			

JN040769		D	Human	x			
JN040779		D	Human	x			
JN040818		D	Human	x			
JN040822		D	Human	x			
JN257160		D	Human	x			
JN257162		D	Human	x			
JN257165		D	Human	x			
JN257172		D	Human	x			
JN257177		D	Human	x			
JN257190		D	Human	x			
JN257202		D	Human	x			
JN642133		D	Human	x			
JN642135		D	Human	x			
JN642136		D	Human	x			
JN642149		D	Human	x			
JN642159		D	Human	x			
JN664913		D	Human	x			
JN664919		D	Human	x			
JN664920		D	Human	x			
JN664921		D	Human	x			
JN664922		D	Human	x			
JN664931		D	Human	x			
JN664932		D	Human	x			
JN664936		D	Human	x			
JN688683		D	Human	x			
JN688695		D	Human	x			
JN688712		D	Human	x			
JN688713		D	Human	x			
JN792912		D	Human	x			
JQ707529		D	Human	x			
JQ707699		D	Human	x			
JX470760		D	Human	x			
KC774444		D	Human	x			
KC875342		D	Human	x			
KF192832		D	Human	x			
KF679990		D	Human	x			
KJ647353		D	Human	x			
KJ647355		D	Human	x			
KJ843187		D	Human	x			
KM524338		D	Human	x			
KM524358		D	Human	x			
KM577668		D	Human	x			
KP090181		D	Human	x			

KP168419		D	Human	x			
KU668435		D	Human	x			
KU736927		D	Human	x			
KX357622		D	Human	x			
L27106		D	Human	x			
X65258		D	Human	x			
X80926		D	Human	x			
GQ205382		D	Human	x	x	x	
GQ205385		D	Human	x	x	x	x
JN315779	Korean Mummy	D	Human	x	x	x	x
AB048701		D	Human	x	x	x	
AB033558		D	Human		x	x	
AB033559		D	Human		x	x	
FJ899792		D1	Human		x	x	
JN642140		D1	Human		x	x	
GQ477455		D2	Human		x	x	
JN642160		D2	Human		x	x	
GQ477453		D2	Human	x	x	x	
JN642163		D2	Human	x	x	x	x
JN688710		D3	Human	x	x	x	
JN688711		D3	Human	x	x	x	x
HE974378		D4	Human		x	x	
KJ470898		D4	Human		x	x	
KJ470896		D4	Human		x	x	x
GQ922005		D4	Human	x	x	x	
KJ470893		D4	Human	x	x	x	
FJ904436		D7	Human		x	x	
FJ904430		D7	Human		x	x	x
X75664		E	Human		x	x	
X75657		E	Human		x	x	x
AB106564		E	Human	x			
AB219533		E	Human	x			
AB274977		E	Human	x			
EU239220		E	Human	x			
FN545823		E	Human	x			
FN594751		E	Human	x			
FN594760		E	Human	x			
GQ161775		E	Human	x			
HM363586		E	Human	x			
HM363592		E	Human	x			
HM363603		E	Human	x			
HM363611		E	Human	x			
KU736913		E	Human	x			

AB166850		F	Human		x	x	
AB214516		F	Human	x			
AB365453		F	Human	x			
DQ899142		F	Human	x			
DQ899145		F	Human	x			
DQ899148		F	Human	x			
JN688720		F	Human	x			
JN792921		F	Human	x			
JQ272888		F	Human	x			
KF199901		F	Human	x			
KJ638660		F	Human	x			
KJ638662		F	Human	x			
KJ676694		F	Human	x			
X75658		F	Human	x			
AY090458		F1a	Human	x	x	x	x
FJ657525		F1b	Human		x	x	
AB116654		F1b	Human	x	x	x	x
AY311369		F2a	Human		x	x	
AY090455		F2a	Human		x	x	x
DQ899146		F2b	Human		x	x	
DQ899144		F2b	Human		x	x	x
AB116549		F3	Human		x	x	
X75663		F3	Human	x	x	x	
AF223962		F4	Human		x	x	
AB056513		G	Human		x	x	
AF405706		G	Human		x	x	
AB064312		G	Human		x	x	x
HE981175		G	Human	x			
AB375163		H	Human		x	x	
AY090454		H	Human		x	x	
AY090457		H	Human		x	x	
AB059659		H	Human	x			
AB059660		H	Human	x	x	x	x
AB486012		J	Human		x	x	x
AF222323			Chimpanzee		x	x	
FM209516			Gibbon		x	x	
AJ131567			Gorilla		x	x	x
AY781180			Gibbon		x	x	x
EU155824			Orangutan		x	x	x
U46935			Gibbon		x	x	x
AB032432			Chimpanzee	x			
AB823658			Gibbon	x			
AB823659			Gibbon	x			

AB823660			Gibbon	x			
AB823661			Gibbon	x			
AB823662			Gibbon	x			
AF193864			Orangutan	x			
AF222322			Chimpanzee	x			
AF242586			Chimpanzee	x			
AF498266			Chimpanzee	x			
AJ131569			Gibbon	x			
AJ131574			Gibbon	x			
AM117396			Chimpanzee	x			
AY077735			Gibbon	x			
AY077736			Gibbon	x			
AY330912			Gibbon	x			
AY330913			Gibbon	x			
AY330914			Gibbon	x			
AY330915			Gibbon	x			
AY330916			Gibbon	x			
AY330917			Gibbon	x			
AY781182			Gibbon	x			
AY781186			Gibbon	x			
AY781187			Gibbon	x			
EU155821			Orangutan	x			
EU155829			Human	x			
FJ798098			Chimpanzee	x			
JQ664503			Gorilla	x			
AF193863			Orangutan	x	x	x	x
AJ131571			Gibbon	x	x	x	x
AY330911			Chimpanzee	x	x	x	x
ERR2299806	Petersberg		Human	x	x	x	x
ERR2299807	Karsdorf		Human	x	x	x	x
ERR2299808	Sorsum		Human	x	x	x	x
LT992438	DA29		Human	x	x	x	x
LT992439	DA27		Human	x	x	x	x
LT992440	DA119		Human	x	x	x	x
LT992441	DA195		Human	x	x	x	x
LT992442	DA45		Human	x	x	x	x
LT992443	RISE563		Human	x	x	x	x
LT992444	DA51		Human	x	x	x	x
LT992447	RISE387		Human	x	x	x	x
LT992448	RISE386		Human	x	x	x	x
LT992454	DA222		Human	x	x	x	x
LT992455	RISE154		Human	x	x	x	x
LT992459	RISE254		Human	x	x	x	x

MG585269	NASD24SEQ (Italian mummified individual)		Human	x	x	x	x
AB032433			Chimpanzee		x	x	

Table S8: Accession numbers of source samples used for SourceTracker2 analysis.

ENA / SRA Accession ID	Tissue (Source)	Source
ERR3307045-ERR3307054	Modern calculus	Velsko et al, 2019
ERS1052224	Soil sample desert Egypt	10.1371/journal.pone.0024452
SRR061294	Subgingival plaque	HMP
SRR061302	Subgingival plaque	HMP
SRR062298	Subgingival plaque	HMP
SRR062299	Subgingival plaque	HMP
SRR062302	Subgingival plaque	HMP
SRR062303	Subgingival plaque	HMP
SRR062332	Subgingival plaque	HMP
SRR062333	Subgingival plaque	HMP
SRR062339	Subgingival plaque	HMP
SRR062343	Subgingival plaque	HMP
SRR062351	Subgingival plaque	HMP
SRR062421	Subgingival plaque	HMP
SRR062477	Subgingival plaque	HMP
SRR062501	Subgingival plaque	HMP
SRR346694	Subgingival plaque	HMP
SRR513449	Subgingival plaque	HMP
SRR513768	Subgingival plaque	HMP
SRR514329	Subgingival plaque	HMP
SRS013950	Subgingival plaque	HMP
SRS014477	Subgingival plaque	HMP
SRS014691	Subgingival plaque	HMP
SRS015064	Subgingival plaque	HMP
SRS019029	Subgingival plaque	HMP
SRS019129	Subgingival plaque	HMP
SRS063215	Subgingival plaque	HMP
SRS011098	Supragingival plaque	HMP
SRS011255	Supragingival plaque	HMP
SRS013533	Supragingival plaque	HMP
SRS014476	Supragingival plaque	HMP
SRS014894	Supragingival plaque	HMP
SRS015378	Supragingival plaque	HMP
SRS015650	Supragingival plaque	HMP
SRS015989	Supragingival plaque	HMP

Table S9: Bacterial composition on Phylum level (without Clostridia, normalized data counts). The darker the green, the higher the percentage (0-100%).

	Bone					Tooth				
**Phyla**	FIP [%]	PPP [%]	PP [%]	RP [%]	ALL [%]	FIP [%]	PPP [%]	PP [%]	RP [%]	ALL [%]
Firmicutes	-	58.44	48.69	58.66	55.17	-	22.88	30.96	90.47	37.88
Actinobacteria	-	17.38	27.24	27.86	24.31	-	49.3	31.42	5.44	35.1
Proteobacteria	-	18.18	19.19	9.96	15.74	-	17.86	31.06	2.1	19.69
Bacteroidetes	-	2.48	1.81	1.05	1.77	-	3.27	1.63	0.17	2.13
Chloroflexi	-	0.02	0.04	0.03	0.03	-	2.36	0.1	0.02	1.14
Fusobacteria	-	1.77	0.69	1.21	1.21	-	1.83	2.2	1.12	1.83
Spirochaetes	-	0.15	0.38	0.17	0.24	-	0.64	0.35	0.13	0.44
C.Saccharibacteria	-	0	0	0	0	-	0.23	0.02	0	0.12
Tenericutes	-	1.08	1.12	0.41	0.87	-	0.74	0.94	0.26	0.72
Gemmatimonadetes	-	0.04	0.05	0.03	0.04	-	0.03	0.06	0	0.03
Cyanobacteria	-	0.11	0.23	0.19	0.18	-	0.17	0.5	0.04	0.26
Deinococcus-Thermus	-	0.06	0.12	0.04	0.07	-	0.09	0.1	0.01	0.08
Planctomycetes	-	0.05	0.09	0.03	0.06	-	0.07	0.09	0	0.06
Thermotogae	-	0.1	0.14	0.17	0.14	-	0.21	0.29	0.18	0.23
Chlorobi	-	0.01	0.02	0.02	0.02	-	0.02	0.02	0	0.02
Acidobacteria	-	0.01	0.02	0.01	0.01	-	0.02	0.02	0	0.02
Verrucomicrobia	-	0.02	0.04	0.01	0.02	-	0.05	0.05	0	0.04
Synergistetes	-	0	0.01	0.01	0.01	-	0.09	0.01	0.01	0.04
Aquificae	-	0.01	0.03	0.03	0.02	-	0.02	0.05	0	0.03
Nitrospirae	-	0.01	0.01	0.02	0.01	-	0.02	0.02	0	0.02
Thermodesulfobacteria	-	0.01	0.01	0.02	0.01	-	0.01	0.03	0.02	0.02
Deferribacteres	-	0.01	0.01	0.02	0.02	-	0.02	0.02	0.02	0.02
Chlamydiae	-	0.01	0.01	0.01	0.01	-	0.01	0.01	0	0.01

Phyla	Calculus					Soft tissue					HMP
	FIP [%]	PPP [%]	PP [%]	RP [%]	ALL [%]	FIP [%]	PPP [%]	PP [%]	RP [%]	ALL [%]	Modern [%]
Firmicutes	11.81	-	46.05	10.86	16.9	-	50.53	20.81	60.16	30.19	18.41
Actinobacteria	44.06	-	29.77	58.46	48.9	-	2.18	12.19	5.73	9.21	26.6
Proteobacteria	28.67	-	15.34	26.91	25.63	-	12.84	46.41	17.45	36.22	19.35
Bacteroidetes	6.34	-	2.69	1.97	3.57	-	4.44	4.4	1.82	4.34	19.05
Chloroflexi	3.15	-	1.05	0.13	1.3	-	0.12	0.16	0	0.15	0
Fusobacteria	2.36	-	1.66	0.23	1.19	-	17.33	4.72	8.07	8.34	11.94
Spirochaetes	1.71	-	0.89	0.08	0.77	-	1.96	1.28	1.82	1.49	4.3
C.Saccharibacteria	1.37	-	0.58	0.01	0.57	-	0	0	0	0	0.33
Tenericutes	0.17	-	0.95	0.13	0.28	-	4.29	5.86	2.6	5.33	0.01
Gemmatimonadetes	0.01	-	0.04	0.51	0.26	-	0	0.08	0	0.06	0
Cyanobacteria	0.1	-	0.33	0.14	0.16	-	1.12	1.19	0.26	1.15	0
Deinococcus-Thermus	0.02	-	0.07	0.22	0.13	-	0	0.18	0	0.13	0
Planctomycetes	0.05	-	0.16	0.11	0.09	-	0.1	0.39	0	0.3	0
Thermotogae	0.03	-	0.17	0.03	0.05	-	2.41	0.93	1.04	1.35	0
Chlorobi	0.02	-	0.01	0.05	0.03	-	0.17	0.15	0	0.15	0
Acidobacteria	0.02	-	0.02	0.04	0.03	-	0.07	0.11	0	0.1	0
Verrucomicrobia	0.01	-	0.03	0.04	0.03	-	0.02	0.11	0	0.08	0
Synergistetes	0.04	-	0.01	0.01	0.02	-	0.02	0	0	0.01	0
Aquificae	0.01	-	0.04	0.01	0.02	-	0.42	0.15	0.52	0.24	0
Nitrospirae	0.01	-	0.02	0.02	0.02	-	0.1	0.14	0.26	0.13	0
Thermodesulfobacteria	0.01	-	0.03	0.01	0.01	-	0.37	0.09	0	0.17	0
Deferribacteres	0.01	-	0.01	0.01	0.01	-	0.27	0.1	0	0.15	0
Chlamydiae	0	-	0.02	0.01	0.01	-	0.1	0.28	0	0.22	0

Table S10: Mapping result of Abusir1519c to the human mitochondrial genome.

	**Abusir1519c**
# reads after C&M prior mapping	451537642
# mapped reads prior DeDup	4797
# of Duplicates removed	1218
Mapped Reads after DeDup	3579
Endogenous DNA (%)	0.001
Cluster Factor	1.34
Mean Coverage	9.2566
std. dev. Coverage	4.5007
Coverage >= 5X in %	87.94
DMG 1st Base 3'	0.1077
DMG 2nd Base 3'	0.0696
DMG 1st Base 5'	0.1011
DMG 2nd Base 5'	0.0881
Average fragment length	42.91
median fragment length	41
GC content in %	42.08
Haplogroup	N1a1a3
Modern human DNA Contamination	1%
